# EXCAVATOR: detecting copy number variants from whole-exome sequencing data

**DOI:** 10.1186/gb-2013-14-10-r120

**Published:** 2013-10-30

**Authors:** Alberto Magi, Lorenzo Tattini, Ingrid Cifola, Romina D’Aurizio, Matteo Benelli, Eleonora Mangano, Cristina Battaglia, Elena Bonora, Ants Kurg, Marco Seri, Pamela Magini, Betti Giusti, Giovanni Romeo, Tommaso Pippucci, Gianluca De Bellis, Rosanna Abbate, Gian Franco Gensini

**Affiliations:** 1 Department of Clinical and Experimental Medicine, University of Florence, Florence, Italy; 2 Laboratory of Molecular Genetics, G. Gaslini Institute, Genoa, Italy; 3 Institute for Biomedical Technologies, National Research Council, Segrate, Milano, Italy; 4 Laboratory of Integrative Systems Medicine (LISM), Institute of Informatics and Telematics and Institute of Clinical Physiology, National Research Council, Pisa, Italy; 5 Diagnostic Genetic Unit, Careggi Hospital, Florence, Italy; 6 Dipartimento di Biotecnologie Mediche e Medicina Traslazionale (BIOMETRA), University of Milan, Milan, Italy; 7 Medical Genetics Unit, Department of Medical and Surgical Sciences, University of Bologna, Bologna, Italy; 8 Institute of Molecular and Cell Biology, University of Tartu, Tartu, Estonia

## Abstract

We developed a novel software tool, EXCAVATOR, for the detection of copy number variants (CNVs) from whole-exome sequencing data. EXCAVATOR combines a three-step normalization procedure with a novel heterogeneous hidden Markov model algorithm and a calling method that classifies genomic regions into five copy number states. We validate EXCAVATOR on three datasets and compare the results with three other methods. These analyses show that EXCAVATOR outperforms the other methods and is therefore a valuable tool for the investigation of CNVs in largescale projects, as well as in clinical research and diagnostics. EXCAVATOR is freely available at http://sourceforge.net/projects/excavatortool/.

## Background

Copy number variants (CNVs) are operationally defined as 50 bp or larger DNA segments [1] that are present at a variable copy number in comparison with a reference genome. CNVs have been demonstrated to be one of the main sources of genomic variation in humans [2-10] and have been shown to participate in phenotypic variation and adaptation by disrupting genes and altering gene dosage. Some CNVs are found in normal individuals, while others contribute to causing various diseases including cancer, cardiovascular disease, HIV acquisition and progression, autoimmune diseases and Alzheimer’s and Parkinson’s diseases [11,12].

In the last few years, several high-throughput sequencing (HTS) platforms [13-15] have emerged that, by simultaneously sequencing billions of short DNA fragments (reads), can be used to sequence a full human genome per week at a cost 400-fold less than previous methods. The development of these HTS platforms has made large-scale re-sequencing projects possible, such as the 1000 Genomes Project and the Cancer Genome Atlas, but their computational complexity still limits the routine use of whole-genome sequencing to individual smaller projects. Whole-exome sequencing (WES), which is the sequencing of all the coding regions of a genome, is a very effective alternative to whole-genome sequencing and has been successfully used to discover common and rare single nucleotide variants (SNVs), small insertions/deletions (indels) and breakpoints of structural variation [16,17].

Although WES is a powerful tool for investigating the great majority of genomic variants, it is unsuitable for analyzing CNVs: the sparse nature of the target and the non-uniform read-depth among captured regions make WES data unsuitable for read-pair [18,19] or split-read [20,21] algorithms and make the read count (RC) approach particularly challenging [22-24]. At present, there are a few publicly available tools that can identify CNVs from WES data using the RC approach: ExomeCNV [25], CoNIFER [26], XHMM [27] and CONTRA [28].

ExomeCNV was the first tool implemented to detect CNVs from WES data. It uses a two-step normalization procedure to mitigate systematic biases due to GC content and mappability, and it estimates copy number values using an uncalibrated read depth. Depending upon batch effects, this can result in the algorithm reporting a significant fraction of the exome as non-diploid. ExomeCNV uses the circular binary segmentation (CBS) algorithm [29] to detect the boundaries of altered regions. CBS does not take into account the distance between adjacent exons and this can lead to it missing large and small genomic alterations in sparsely targeted regions, when applied to WES data [30]. CoNIFER and XHMM exploit singular value decomposition (SVD) and principal-component analysis (PCA) to identify and remove the principal sources of variation underlying the non-uniform read depth of captured regions. The SVD and PCA normalization procedures require the analysis of many samples at once, thus limiting their application to sequencing projects with a large number of samples.

CONTRA uses a base-level log-ratio strategy to remove GC content bias and correct for the library size effect. Nevertheless, it has been demonstrated that the ratio between the RCs of case and control samples is not able to remove GC content bias completely [31]. Moreover, all of these tools classify each genomic region according to a three-state classification scheme (deletion, normal and amplification), which does not discriminate between two- and single-copy deletions and between three- and multiple-copy amplifications, thus limiting the potential of RC data to predict the exact number of DNA copies.

To overcome the limitations of existing methods in detecting genomic regions involved in CNV using WES data, we developed a novel software package, EXCAVATOR (EXome Copy number Alterations/Variations annotATOR), which uses a RC approach. We studied the systematic biases of sequencing data causing the non-uniform read depth of captured regions and we developed a three-step normalization procedure that mitigates the effects of these biases. To take into account the sparseness of WES data throughout the genome, we developed a novel segmentation algorithm that exploits the distances between consecutive exons to improve the detection of small and large altered regions covered by few exons. Finally, we combined our normalization and segmentation methods with a calling procedure to classify each genomic region as one of five discrete copy number states and we packaged everything into the EXCAVATOR software tool.

We tested the EXCAVATOR pipeline by analyzing three different WES datasets: a population dataset generated by the 1000 Genomes Project Consortium and two datasets generated in our labs comprising melanoma cancer and intellectual disability samples. To evaluate its performance, we compared the results obtained by EXCAVATOR with three other state-of-the-art pipelines. Furthermore, we validated the results obtained by EXCAVATOR using copy number profiles generated by SNP array technology, demonstrating its power and versatility for discovering small and large genomic regions involved in CNVs.

## Results and discussion

### Data biases and correction

To study DNA copy number variations from targeted sequencing data, we consider the mean number of reads aligned to each exon, that is the exon mean read count (EMRC). EMRC is defined as:

(1) $${\text{EMRC}}_{e}=\frac{{\text{RC}}_{e}}{L_{e}}$$

where RC _*e*_ is the number of reads aligned to a target genomic region *e* and *L*_*e*_ is the size of that same genomic region (in base pairs). EMRC is calculated for each targeted region of the genome and gives a measure of the density of reads aligned to that particular region. To study the statistical properties and the sources of bias of EMRC data we exploited the WES data of eight individuals sequenced by the 1000 Genomes Project Consortium (NA10847, NA19131, NA19138, NA19152, NA19153, NA19159, NA19206 and NA19223); see Additional file 1 for more details.

**Figure 1:**
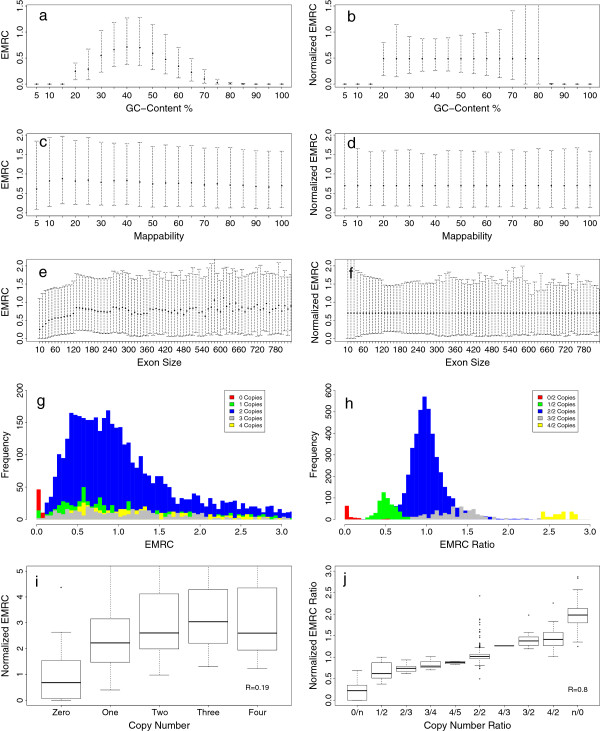
**EMRC data biases, normalization and CNV prediction ability. (a)**, **(c)**, **(e)** Correlation between EMRC data and the three bias types due to GC content percentage **(a)**, genomic mappability **(c)** and exon size **(e)**. **(b)**, **(d)**, **(f)** The effect of the median normalization procedures on the removal of the three bias sources: GC content percentage correction **(b)**, genomic mappability correction **(d)** and exon size correction **(f)**. The upper border of the dashed lines is the 90th percentile of the EMRCs, while the lower border is the 10th percentile. **(g)**, **(h)**, **(i)**, **(j)** Histograms and boxplots summarizing the capability of EMRC data to predict the exact number of DNA copies of a CNV region. **(g)** and **(i)** show the prediction capability for single-sample EMRC data, while **(h)** and **(j)** are the prediction capability for the EMRC ratio. EMRC ratios were calculated by using the NA10847 sample as control. These calculations were performed using several broad genomic regions that were previously reported to have copy numbers equal to 0, 1, 2, 3 and 4 by McCarroll *et al.*[7] in the eight samples from the 1000 Genomes Project. *R* is the Pearson correlation coefficient.CNV, copy number variant; EMRC, exon mean read count.

First, we studied the relation between EMRC and three bias sources: the local GC content percentage, the genomic mappability and the size of the targeted regions (see Materials and methods for more details). The results of these analyses are shown in Figure 1. In agreement with previous reports [31-34], we observed that EMRC is strongly correlated to the local GC content percentage: it is highest for values of GC content between 35% and 60% while it decreases at both extremes (Figure 1a). As previously reported for RC data [31], we found that EMRCs are affected by genomic mappability: the larger the mappability score, the smaller the EMRC distribution variance. Moreover, mappability affects the mean number of aligned reads (Figure 1c). Interestingly, our analysis indicated that the mean number of reads aligned to a targeted region of the genome is correlated to the size of that region. In particular, for exons smaller than 150 bp, we found that the EMRC value grows as a function of targeted region size, while for exons larger than 150 bp, EMRC reaches a plateau and remains constant (Figure 1e). These results show that EMRC data require a normalization step before being used to detect genomic regions involved in CNVs.

To minimize the effect of these sources of variation and make the data within and between samples comparable, we implemented a three-step bias removal procedure based on the median normalization approach introduced in [23] for the removal of the GC content effect and extended in [31] for mitigating mappability bias (see Materials and methods for more details). To evaluate the performance of the median normalization procedures described in the Materials and methods section, we applied them to the WES data of the eight samples generated by the 1000 Genomes Project Consortium. The normalized data show in Figure 1b,d,f demonstrate that median normalization approaches are able to mitigate the effect of all three bias sources, equalizing the mean level of each bin to the same master mean.

Since the first exon of each gene is GC richer than the final and internal exons, this bias can affect the detection of CNVs that include first exons. To investigate the capability of our normalization procedure to mitigate the first exon effect, we compared the distribution of EMRC values for first and all other exons before and after the normalization step. The results of this analysis are reported in Additional file 1: Figure S1. As expected, the mean level of EMRC values for first exons is smaller than EMRC values for internal and final exons. Nevertheless, normalization allows for the removal of this difference, equalizing the mean levels of EMRC values for first exons and all other exons. Next, to understand the capability of EMRC data to predict the exact DNA copy number values of a genomic region, we examined several broad genomic regions that were previously reported to have copy numbers equal to 0, 1, 2, 3 or 4 by McCarroll *et al.*[7] for the eight samples (see Materials and methods). In this analysis we compared the distribution and the CNV prediction capability for both single-sample EMRC data and the ratio between EMRC data from two samples.

The histograms in Figure 1g show that for single-sample data (with the median normalized to copy number two), the EMRC distributions for genomic regions with different DNA copy number states have a significant overlap and completely fail to predict the exact number of copies, as shown in Figure 1i, where the Pearson correlation coefficient calculated between the real and predicted DNA copy number values is *R *= 0.19. On the other hand, the EMRC ratio between two samples allows for a better discrimination of genomic regions with different numbers of DNA copies, as illustrated in Figures 1h and 1j, where the Pearson correlation coefficient between the real and predicted DNA copy number values is *R *= 0.80. Remarkably, as shown in Figure 1j, normalized ERMC ratios can distinguish between even intermediate CN ratios, such as 2/3, 3/4, 4/5 and 3/2, 4/3, 4/2, despite their overlapping distributions. For these reasons, in all the analyses we performed for this work, we decided to use the ratio between EMRC data from test and control samples to identify genomic regions involved in CNVs: in particular, we chose to use the log-transformed ratio (log_2_ ratio) between test and control samples normalized with the LOWESS scatter plot normalization procedure (see Additional file 1 for more details).

### Segmentation and calling algorithms

After EMRC bias correction, we calculated the logarithm of the ratio between test and control samples (log_2 _ratio) and we sorted the data with respect to their genomic position. The obtained signal is mathematically very similar to those generated by RC analysis [31]: deletions (or amplifications) are identified as a signal decrease (or increase) across multiple consecutive targeted regions. For this reason, as in RC data analysis, the log_2 _ratios of EMRC data need to undergo a segmentation step to detect the boundaries of the genomic regions with altered DNA copy number. The only difference between RC and EMRC data is the distance between consecutive genomic regions: RCs are estimated for non-overlapping and contiguous genomic windows with predefined lengths, while EMRCs are calculated for genomic windows (corresponding to targeted regions) with different sizes and variable distance. The distance between consecutive exons within the same gene ranges from few base pairs to 100 kb (with a median value of 1500 bp), while the distance between consecutive genes (calculated as the distance between the final exon of a gene and the first exon of the subsequent gene) ranges from hundreds of base pairs to millions of base pairs (with a median value of 25 kb). For this reason, we can find genomic regions comprising a large number of exons as well as highly isolated genomic regions with few exons using the log_2 _ratio of EMRC profiles.

To take into account this peculiar characteristic of EMRC data, we extended the shifting level model (SLM) segmentation algorithm [22,35] to include the distance between consecutive exons (defined as the distance between the midpoints of consecutive exons). In SLM, sequential observations *x *= (*x*_1_,…,*x*_*i*_,…,*x*_*N*_) are considered to be realizations of the sum of two independent stochastic processes *x*_*i *_= *m*_*i *_+ *ε*_*i*_, where *m*_*i *_is the unobserved mean level and *ε*_*i *_is normally distributed white noise. The mean level *m*_*i *_does not change for long intervals and its duration follows a geometric distribution: the probability that *m*_*i *_takes a new value at any point *i *is regulated by the parameter *η*. We included the distance between consecutive exons (*d*_*i*_) in the SLM by defining the parameter *η *as:

(2) $$\eta (d_{i})∼\text{exp}\left[\frac{1}{d_{i}/d_{\text{Norm}}}\right]$$

where *d*_Norm _is a distance normalization parameter. We thus obtained a heterogeneous shifting level model (HSLM) in which as the genomic distance between consecutive exons increases, so does the probability of jumping from one state to another. This feature allows the HSLM algorithm to detect both highly isolated genomic regions covered by few exons and large genomic regions covered by many exons with a comparable accuracy. A detailed description of the heterogeneous shifting level model and its algorithm is given in Additional file 1.

Once the log_2 _ratios have been segmented with the HSLM algorithm, each segment needs to be classified as a discrete copy number state. As reported in the Background section, all of the recently published tools can classify genomic regions using a three-state classification scheme (deletion, normal and amplification), which limits the potential of RC data to predict two-copy deletions and multiple-copy amplifications. To overcome these limitations, we decided to exploit the FastCall algorithm [36], which we developed to classify array-CGH (comparative genomic hybridization) data, by applying it to WES data. The FastCall algorithm can classify each segmented region using a five-state classification scheme (two-copy deletion, one-copy deletion, normal, one-copy duplication and multiple-copy amplification) and thus we can discriminate double-copy from single-copy deletions and single-copy from multiple-copy duplications (see Materials and methods for more details). All the algorithms and methods described above have been packaged in the EXCAVATOR software (see Materials and methods).

To test the ability of the HSLM algorithm to detect CNVs of different sizes as a function of the distance between consecutive exons, we performed an intensive simulation based on synthetic data. Synthetic chromosomes were generated from the EMRC data of the eight samples described above and previously characterized by [7]: there were seven samples of Yoruba ancestry (NA19131, NA19138, NA19152, NA19153, NA19159, NA19206 and NA19223) and one sample of Caucasian ancestry (NA10847). The EMRC data were first corrected for the three bias sources and then the EMRC log_2 _ratio was calculated using each possible combination with one sample as control and the other seven samples as tests. To reproduce the complex architecture of exome data, we generated synthetic chromosomes using synthetic genes as building blocks. Each synthetic gene, with the exception of *g *genes (the altered genes), has a random number of exons sampled from a uniform distribution *U *(5,100) (that is, the number of exons ranges from 5 to 100). The number of exons in the altered genes is defined by the integer parameter *N *and the total number of exons in each synthetic chromosome is constrained to be 1,000. The distances between adjacent exons that belong to the same gene are sampled from a uniform distribution *U *(10,10000) (ranging from 10 to 10,000 bp), while the distance between adjacent genes is set equal to a predefined distance *D*. The DNA copy number values of each synthetic chromosome were generated by exploiting the results reported in [7]. To simulate normal copy regions, we sampled (1000 − *N*) log2 ratio data from genomic regions previously predicted as two-copy in [7] for both test and control samples and to simulate one-copy (three-copy) regions, we sampled *N *log2 ratio data from regions previously predicted as one-copy (three-copy) for the test sample and two-copy for the control sample.

We performed simulations with *g *= [ 1,2,3,4,5], *N *= [ 2,3,5,10,20,50] and *D *= [10 kb, 50 kb, 100 kb, 500 kb, 1 Mb, 5 Mb] and for all combinations of *g*, *N *and *D *we generated 1,000 synthetic chromosomes: all the synthetic datasets were analyzed using different values of the parameter *D*_Norm _(10^3^, 10^4^, 10^5 ^or 10^6^).

**Figure 2:**
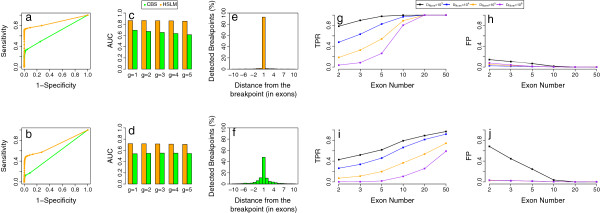
**Performance evaluation of the HSLM algorithm for detecting CNVs in synthetic chromosomes. (a)**, **(b)** ROC curves comparing the sensitivity and specificity of the HSLM and CBS algorithms in the detection of one-copy **(a)** and three-copy CNVs **(b)**. **(c)**, **(d)** Comparisom of the HSLM and CBS algorithms when analyzing synthetic chromosomes with different numbers (*g* = [ 1,2,3,4,5]) of one-copy **(c)** and three-copy **(d)** genes. **(e)**, **(f)** Performance of the HSLM **(e)** and CBS **(f)** algorithms in detecting the correct breakpoint position. The *x* axis is the distance between the predicted and the correct position. The *y* axis is the percentage of breakpoints predicted at a given distance from the correct position. **(g)**, **(h)**, **(i)**, **(j)** TPR and FP plots for different values of the *D*_Norm_ parameter versus exon number in the segmented region. **(g)** and **(h)** show TPR and FP when analyzing one-copy regions. **(i)** and **(j)** are TPR and FP when analyzing three-copy regions. Each curve point was obtained by averaging across 5,000 simulations (1,000 synthetic chromosomes for *g* = [ 1,2,3,4,5]).CBS, circular binary segmentation; CNV, copy number variant; FPR, false positive rate; HSLM, heterogeneous shifting level model; TPR, true positive rate.

To assess the accuracy of HSLM in detecting CNVs at the boundaries (breakpoint detection) we computed the receiver operating characteristic (ROC) curve as in [37] and we compared its performance to that of the circular binary segmentation (CBS) algorithm [29], which has been used in other traditional packages for exome-CNV analysis, such as ExomeCNV [25] and VarScan2 [30]. The results of these analyses are summarized in Figure 2a,b,c,d and Additional file 1: Figures S2 to S49. Overall they show that our segmentation algorithm outperforms the CBS method in both sensitivity and specificity for all the alteration sizes we simulated. Panels c and d of Figure 2 also show that the larger the number of altered regions in a chromosome, the lower the accuracy of the CBS method. On the other hand, increasing the number of altered regions in a chromosome does not affect the global performance of HSLM. Remarkably, synthetic analysis indicates there is a difference in the accuracy of detection of genomic regions with one copy and three copies. Both CBS and HSLM detect one-copy regions with higher sensitivity than three-copy regions and this behavior can be ascribed to two main reasons. The first is numerical: the signal shift for three-copy regions (log2(3/2) = 0.58) is smaller than the signal shift for one-copy regions (log2(1/2) = − 1) and the segmentation algorithms are sensitive to the extent of this shift. The second reason lies in the fact that the variance of RC data is lower for deleted states (zero or one copy) and it proportionally increases with copy number values [23]: the larger the variance, the smaller the sensitivity of segmentation algorithms in detecting signal shifts.

As a further test, to assess the ability of our segmentation algorithm to identify the exact breakpoint of a CNV region correctly, for each synthetic chromosome we calculated the distance (in exons) between the predicted and the correct breakpoint positions and we compared its performance with CBS. The results of these analyses are shown in the histograms of Figure 2c,d, which show that HLSM can correctly detect the exact position of 94% of the breakpoints on synthetic chromosomes, while CBS predicted the exact position only of 50% of the breakpoints. Finally, to evaluate the capability of the HSLM and FastCall procedures in discovering CNVs, we exploited the method reported in [23] and [22]: a detected segment is considered a true positive (TP) if there is at least a 50% overlap between the detected segment and the synthetic altered region, while it is considered a false positive (FP) if there is no overlap with a synthetic altered region. Moreover, to better investigate the FP events detected by HSLM we generated synthetic chromosomes with no altered regions (*g *= 0). The true positive rate (TPR) and false positive (FP) plots reported in Figure 2g,h,i,j and Additional 1: Figures S50 to S56 show that the larger the distance between adjacent genes (*D*) the higher the sensitivity of HSLM in detecting genomic alterations. This feature is a direct consequence of how we modeled the parameter *η *(*d*_*i*_) of the HSLM (the larger the genomic distance *D* the larger the probability of jumping from one mean level *m*_*i *_to another *m*_*i *+ 1_) and this allows our algorithm to detect both highly isolated genomic regions covered by few exons and large genomic regions covered by many exons with a comparable accuracy. For genomic distances *D *smaller than 500 kb, we were able to detect one-copy regions with ten exons (TPR = 0.99) and three-copy regions with 20 exons (TPR = 0.8), while for *D *≥ 1 Mb we detected one-copy regions with three exons (TPR = 0.95) and three-copies regions with ten exons (TPR = 0.8). Finally, the analysis of the synthetic chromosomes demonstrated that the *D*_Norm _parameter is fundamental for modulating the resolution of our algorithm. As expected, the results shown in Figure 2 and Additional file 1: Figures S50 to S55 show that the smaller the value of *D*_Norm _the stronger the ability of HSLM to detect small genomic events. On the other hand, small values of the *D*_Norm _also increase the total number of FP events detected. However, in terms of specificity, our method detected a very small number of FP events, the great majority of them (96%) being events that include less than five exons (see panels h and j of Figure 2 and Additional file 1: Figure S56).

### Population data analysis

To show the potential of our analysis pipeline for population genomics studies, we applied EXCAVATOR on the WES data of 20 healthy individuals (seven Utah residents (CEU) with ancestors from northern and Western European, seven Japanese people (JPT) from Tokyo and six Yoruba people (YRI) from Ibadan) using the WES data of an individual of Yoruba ancestry as control (see Table 1). The table shows the total number of samples used as tests and controls, the enrichment kit used to capture coding sequences, the sequencing platform and the sequencing depth obtained for test and control samples.

**Table 1:** Summary statistics of the three datasets analyzed in this paper

**Cohort**	**Test**	**Control**	**Capture**	**HTS**	**Mean depth**	**Mean depth**
	**samples**	**samples**	**version**	**platform**	**on tests**	**on controls**
1000 Genomes Project	20	1	SureSelect	HiSeq2000	83 ×	107 ×
			All Exon V2			
Melanoma	6	6	SureSelect	GA IIx	45 ×	41 ×
			All Exon 50 Mb			
Intellectual disability	2	1	TruSeq	HiSeq2000	63 ×	65 ×
			Exome enrichment			

According to the Fort Lauderdale principle for the use of unpublished data for method development, we give only the CNV regions detected on chromosome 1 and chromosome 4. Globally we detected 101 CNV events (with a median number of five CNV regions per sample), with a minimum of two regions for the NA12760 sample and a maximum of eight regions for the NA10847 sample. The mean size of these regions was approximately 135 kb, with a minimum size of approximately 5 kb in 11 samples (NA10847, NA11840, NA12717, NA12751, NA12760, NA18959, NA18973, NA19138, NA19159, NA19206 and NA19223) and a maximum size of approximately 900 kb in eight samples (NA10847, NA12249, NA12717, NA12751, NA12761, NA18973, NA18959 and NA18981). The complete list of the CNVs detected on chromosomes 1 and 4 is given in Additional file 2: Table S1.

To evaluate the accuracy of our computational approach, we analyzed the data for the 20 healthy individuals using the other three recently published methods for CNV calling from WES data: ExomeCNV, CoNIFER and XHMM (see Materials and methods for analysis settings). As reported in Background section, the performance of SVD and PCA methods depends on concurrently analyzing many samples, so that systematic noise becomes evident and can subsequently be removed. For this reason, to improve the accuracy of CoNIFER and XHMM, we used these two tools by adding 80 extra samples to the 20 used with EXCAVATOR and ExomeCNV (see Additional file 1 for more details). Globally we observed that the total number of CNV events detected by each of the three tools was very different (Table 2). On chromosomes 1 and 4 of the 20 individuals, CoNIFER detected only 9 CNV regions, XHMM 55 CNVs, while ExomeCNV identified 1,791 events (Table 2). Of the 9 CNV regions detected by CoNIFER, 6 (66%) are present only in one sample (rare variants) while 3 (33%) are shared by more than one sample (common variants). Similarly, XHMM detected 12 rare CNVs (21.8%, 12/55) and 43 common variants (78.2%). On the other hand, the great majority of the CNV events detected by EXCAVATOR and ExomeCNV are common variants: EXCAVATOR detected 10% of rare variants (10/101) and ExomeCNV 5% (99/1,791). The large proportion of rare events detected by CoNIFER and XHMM could be related to the normalization methods that are the basis of these two computational pipelines: singular value decomposition (SVD) for CoNIFER and principal component analysis (PCA) for XHMM. PCA and SVD are eigenvalue methods used to reduce a high-dimensional dataset into fewer dimensions while retaining important information. CoNIFER and XHMM use them to determine and filter out the principal components of systematic noise. This filtering strategy can lead to the removal of common CNV signals thus explaining the preferential detection of rare events by these methods. Conversely, ExomeCNV and EXCAVATOR analyze and normalize one sample at a time and do not suffer from this bias.

**Table 2:** **Summary of the CNV events detected by the four tools in the population data analysis**^**a**^

**Sample**	**EXCAVATOR**	**XHMM**	**CoNIFER**	**ExomeCNV**
NA10847	8 (6-2)	3 (3-0)	0 (0-0)	125 (122-3)
NA11840	3 (3-0)	2 (2-0)	0 (0-0)	124 (122-2)
NA12249	3 (3-0)	0 (0-0)	0 (0-0)	128 (128-0)
NA12717	6 (6-0)	4 (4-0)	0 (0-0)	113 (113-0)
NA12751	7 (5-2)	2 (2-0)	0 (0-0)	119 (118-1)
NA12760	2 (2-0)	2 (2-0)	0 (0-0)	126 (126-0)
NA12761	4 (2-2)	4 (3-1)	0 (0-0)	206 (173-33)
NA18959	6 (6-0)	2 (1-1)	0 (0-0)	149 (134-15)
NA18966	3 (3-0)	5 (4-1)	0 (0-0)	39 (35-4)
NA18967	5 (5-0)	2 (1-1)	0 (0-0)	21 (21-0)
NA18970	4 (4-0)	3 (3-0)	0 (0-0)	24 (24-0)
NA18973	7 (7-0)	0 (0-0)	0 (0-0)	91 (91-0)
NA18981	5 (4-1)	3 (3-0)	0 (0-0)	100 (99-1)
NA18999	3 (3-0)	2 (2-0)	0 (0-0)	229 (196-33)
NA19131	8 (6-2)	3 (3-0)	2 (1-1)	30 (30-0)
NA19138	5 (5-0)	3 (2-1)	1 (1-0)	48 (46-2)
NA19153	5 (5-0)	3 (1-2)	1 (0-1)	26 (25-1)
NA19159	4 (4-0)	5 (2-3)	2 (0-2)	28 (27-1)
NA19206	6 (5-1)	3 (2-1)	1 (0-1)	35 (33-2)
NA19223	7 (7-0)	4 (3-1)	2 (1-1)	30 (29-1)
Total	101 (91-10)	55 (43-12)	9 (6-3)	1,791 (1,692-99)

^a^For each sample, columns show the number of all CNV events (common-rare) identified by each tool.

To validate the results obtained by the four methods, we calculated the overlap between the four sets of genomic events and the known CNVs annotated in the database of genomic variants (DGV) and in the NCBI dbVar. For each of the four algorithms, the overlap analysis took into account all the discovered CNVs and rare and common variants separately. The comparison of the four algorithms and the CNVs in DGV and dbVar was performed using two different overlap criteria: a region was considered validated if there was any overlap greater than 10% (criterion A) or 50% (criterion B).

**Figure 3:**
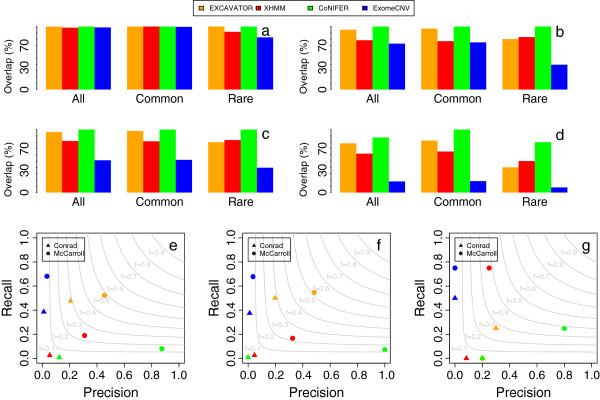
**Summary of the results obtained by EXCAVATOR on the 1000 Genomes Project samples. (a)**, **(b)**, **(c)**, **(d)** Overlap between the set of CNVs detected by the four methods and the CNVs annotated in the DGV **(a, b)** and in the NCBI dbVar **(c, d)** with the two overlapping criteria: 10% **(a, c)** and 50% **(b, d)**. **(e)**, **(f)**, **(g)** Precision-recall plots of the comparison between the CNV events detected by the four methods included in this comparison and the CNVs previously reported by McCarroll *et al.*[7] and Conrad *et al.*[5]. Light grey curves represent *F*-measure levels (harmonic mean of precision and recall). **(e)** Results for all variants. **(f)** Results for common CNVs. **(g)** Results for rare CNVs.

The results of these analyses are summarized in Figure 3a,b,c,d. For the all CNV and common CNV analyses, the best results for the validation rate for the DGV and dbVar databases for both overlap criteria were obtained by EXCAVATOR and CoNIFER, followed by XHMM and ExomeCNV. For the rare CNV analysis, CoNIFER obtained the best validation rates, followed by EXCAVATOR, XHMM and ExomeCNV. As a further step, to evaluate the sensitivity and the specificity of the four methods, we compared the four sets of calls with the CNVs previously reported by McCarroll *et al.*[7] and Conrad *et al.*[5] in the 20 samples included in our study. Also in this case, all the comparison analyses took into account all the discovered CNVs and rare and common variants separately. Using microarray techniques, McCarroll *et al.*[7] detected 100 CNV events (96 common CNVs and 4 rare CNVs) overlapping coding regions (with at least three exons) on chromosomes 1 and 4 of these 20 samples, while Conrad *et al.*[5] detected 120 events (116 common and 4 rare). Of the CNV regions reported by McCarroll *et al.*, 12 out of 100, and 76 out of the 120 reported by Conrad *et al.*, were not found by EXCAVATOR and ExomeCNV, since the test and control samples had the same DNA copy number values for those traits. For this reason, we used the whole reference set of CNVs used by McCarroll *et al. *and Conrad *et al. *to validate the CoNIFER and XHMM results, while EXCAVATOR and ExomeCNV were validated using a reduced dataset with variants having the same copy number status in the test and control samples filtered out. The two reference sets allowed us to evaluate the precision (*P*) and recall (*R*) obtained by the four tools. For each reference set, the precision was calculated as the ratio between the number of correctly detected events (the intersection between the tool calls and the validation set calls) and the total number of events detected by a tool. The recall was calculated as the ratio between the number of correctly detected events and the total number of events in the validation set.

The results obtained by the four methods for the all variants (Figure 3e) and common variants (Figure 3f) validations are very similar. In the McCarroll dataset, CoNIFER obtained excellent results for precision followed by EXCAVATOR, XHMM and ExomeCNV. ExomeCNV was the best for recall, followed by EXCAVATOR, XHMM and CoNIFER. The high recall rate obtained by ExomeCNV is due to the large number of CNV events (see Table 2) detected by this tool. However, the precision for this method is very low since only a very small fraction of the 1,791 events overlap with the McCarroll dataset. In the Conrad dataset, all the methods gave poor results with the exception of our computational pipeline: EXCAVATOR outperformed the other three software packages for both precision and recall.

For the rare variants analysis, we observed that the PCA-based approach performs well with the McCarroll dataset (Figure 3g). CoNIFER obtained high precision and moderate recall, while XHMM obtained high recall and moderate precision. On the other hand, EXCAVATOR gave very poor results: it was not able to identify any of the rare events of the McCarroll dataset, and only two out of the ten rare events detected by our method overlap with the McCarroll dataset. Conversely, for the Conrad dataset, our pipeline achieved the best trade-off between precision and recall while the other three methods completely failed the validation analysis. Taken as a whole, these results highlight that EXCAVATOR outperforms the other state-of-the-art methods considered in this comparison.

### Melanoma data analysis

To evaluate the power of our computational approach for cancer genomics studies, we used EXCAVATOR to analyze six metastatic melanoma cell lines derived from metastasis tumor biopsies of stage IV melanoma patients and six blood samples from healthy donors were used as controls (Table 1). Here, we aimed to test our pipeline with respect to some typical major challenges of cancer genomics analyses, such as the ability to analyze widely rearranged karyotypes, with many different copy number alterations (CNAs) that often result in significant sample diversity. Given these issues, the detection of CNAs in tumor samples and the correct quantification of their DNA copies can be particularly challenging.

**Figure 4:**
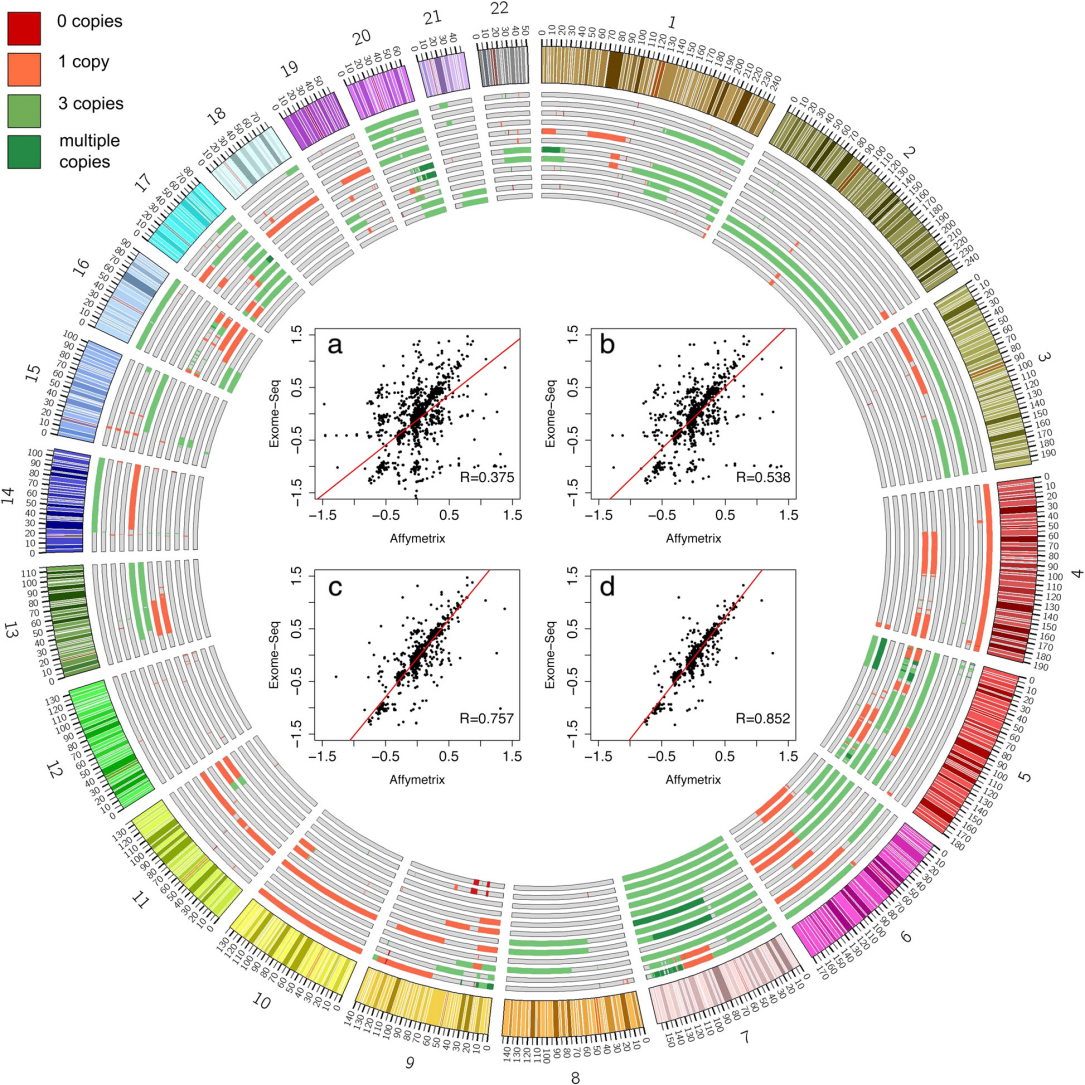
**Summary of the results obtained by EXCAVATOR on the melanoma dataset.** The Circos plot summarizes all the CNV regions detected in each of the six samples by both exome-seq and SNP array analysis. On each chromosome, melanoma samples are vertically ordered (Me01, Me02, Me04, Me05, Me08, Me12), with two tracks (WES and SNP array) for each. Central panels show the global correlation calculated between the log2 ratio median values obtained from the two technologies, when considering all the segmented regions **(a)** or segmented regions larger than 100 kb **(b)**, 500 kb **(c)** or 1 Mb **(d)**. CNV regions are distinguished by color as two-copy deletions (red), one-copy deletions (orange), one-copy amplifications (light green) and multiple-copy amplifications (dark green).CNV, copy number variant.

To evaluate the accuracy and resolution of WES data in discovering CNAs of different kinds and sizes, we also performed genomic profiling of the same 12 samples using the Affymetrix 250K SNP Array platform. For each segmented region, we compared the log_2 _ratio median values obtained from WES and the SNP array and calculated their global correlation over the whole dataset. This calculation was performed considering all the segmented regions or progressively filtering out regions smaller than a threshold (which we set at 100 kb, 500 kb or 1 Mb). The results of this correlation analysis are shown in the central panels of Figure 4. A strong correlation between the SNP array and WES results (*R *= 0.85) was observed for segmented regions larger than 1 Mb. Conversely, considering progressively smaller genomic regions, the correlation between the two platforms drastically decreased mainly due to the different distributions of the SNP probes and exons throughout the genome. This was confirmed by comparing the number of Affymetrix SNP probes and the number of exons that cover each segmented region (Additional file 1: Figure S57): segmented regions larger than 1 Mb comprise a comparable number of SNP probes and exons (*R *= 0.8), while segmented regions smaller than 100 kb do not (*R *= − 0.02).

Another important feature emerging from this correlation analysis is the larger dynamic range provided by WES data: for genomic regions larger than 100 kb we found that the slope of the regression line was greater than 1 and it had a maximum value of 1.5 for regions larger than 1 Mb, thus indicating that over the whole dataset WES data can detect and quantify a wider range of copy number values with respect to SNP array data. The higher dynamic range of WES data is a documented advantage of this technology, which improves the ability of segmentation algorithms to detect signal shifts and the ability of calling algorithms to quantify the correct number of DNA copies. This feature is particularly relevant in cancer genomics analysis, where sample heterogeneity often hampers the detection of CNAs and the correct quantification of their DNA copy number. This is evident also in the melanoma dataset: the Circos plot (Figure 4) shows all the CNAs called by WES and SNP array, for each tumor sample (for complete lists see Additional file 3: Table S2 for WES and Additional file 4: Table S3 for SNP array results). Although the genomic aberrations here found were globally consistent with the typical well-known melanoma signature, it is straightforward to note that on some chromosomes WES and SNP array data returned different results.

All these results are directly related to the different dynamic range and sensitivity peculiar to these two technologies. For many chromosomes across the six tumor samples, WES data called one-copy deletions or one-copy amplifications where SNP array data returned a normal copy number state. In these cases, as shown for chromosomes 4, 7, 10 and 17 in Additional file 1: Figures S57 to S60, the copy number data derived from both technologies showed a shift from the normal diploidy baseline. However, the WES data resulted in a greater shift than SNP array, thus allowing the classification of a region as CNA by the calling algorithm. The same phenomenon explains why, in cases where both technologies detected exactly the same CNA in terms of boundaries, the WES data was able to call multiple-copy amplifications whereas SNP array data called only one-copy gains, as seen on chromosomes 1, 5, 7 and 9 in the Circos plot (Figure 4). Overall, these data demonstrated that, particularly when dealing with cancer samples, the wider dynamic range provided by WES data can be used to obtain a greater sensitivity and, consequently, a better discrimination and quantification of CNAs. Considering these properties, the combination of WES data with the EXCAVATOR pipeline improves the detection of CNAs and, consequently, the identification of potentially interesting genes affected by genomic imbalances that may deserve further investigations as candidate cancer genes. Indeed, as a proof of principle confirming the potential of our method, we observed that on chromosome 7, in three samples (Me04, Me08 and Me12), both WES and SNP array data detected the one-copy gain of a q arm typical of a melanoma signature and encompassing the *BRAF *locus on 7q34 (chr7:140433813-140624564), already known to be affected by genomic amplifications in melanoma cell lines [38]. In addition, EXCAVATOR called such a one-copy gain also in Me02 (whereas SNP array data called a normal diploidy over the whole chromosome), and a multiple-copy amplification in Me01 and Me05, where SNP array data showed only a one-copy gain. Moreover, as examples of known melanoma genes typically affected by deletions, our computational pipeline applied on WES data identified a one-copy loss in two samples (Me01 and Me04) covering the whole chromosome 10 and including the *PTEN *locus on 10q23.31 (chr10:89623195-89728532), which SNP array data completely missed. Similarly, on chromosome 17p, while for Me08 both WES and SNP array data detected a one-copy loss spanning over the *TP53 *locus on 17p13.1 (chr17:7571720-7590868), WES data were able to identify such a deletion also in Me02, whereas SNP array data returned a diploid state. These two genes are well-known tumor suppressor genes and are frequently affected by one-copy deletions in up to 40% of melanoma cell lines [38]. Such situations are visually noticeable in the Circos plot of Figure 4 and are reported in detail in Additional file 1: Figures S58 to S61.

As a final step, since ExomeCNV was purposely developed and calibrated on cancer data, we compared its performance with that of EXCAVATOR in the analysis of the six metastatic melanoma cell lines using the six blood samples from healthy donors as controls (see Materials and methods for analysis settings). The results produced by ExomeCNV clearly indicate an overestimation of CNV events: for almost all melanoma samples, the algorithm detected more than 2 Gb of altered regions (1,950 Mb for Me01, 2,302 Mb for Me02, 2,318 for Me04, 2,168 Mb for Me05, 2,265 Mb for Me08 and 2,168 Mb for Me12). This overestimation of non-diploid regions distributed over most of the exome is due to the fact that ExomeCNV estimates DNA copy number values using an uncalibrated read depth. Overall, these results strongly suggest that EXCAVATOR gives novel and potentially useful improvements and opportunities for cancer genomics.

### Intellectual disability data analysis

To demonstrate the ability of our computational pipeline to detect genomic alterations involved in mental retardation, we performed whole-exome sequencing of two siblings with an intellectual disability (ID1 and ID2); see Table 1. To show the flexibility of our computational pipeline in combining and analyzing data generated by different laboratories, we used, as control, the WES data of a healthy individual of European descent sequenced by [39] (see Materials and methods and Additional file 1 for more details). The data were analyzed using EXCAVATOR with default parameters and the results of this analysis are shown in Additional file 5: Table S4 and summarized in Additional file 1: Figure S62.

For autosomal chromosomes, EXCAVATOR detected 29 CNV regions in the ID1 sample and 24 CNV regions in the ID2 sample, ranging from 1 Mb to 3 kb in size. To distinguish putative pathogenic CNVs from normal copy number polymorphisms, we assessed the overlap between our calls and the known CNVs annotated in the database of genomic variants (DGV) by using a 50% overlap criterion. We found that 22 out of 29 and 17 out of 24 regions overlap with DGV for the ID1 and ID2 samples, respectively. The CNV regions that do not overlap with DGV range from 1 Mb to 26 kb in size. In this set of CNVs, we found a large deletion on chromosome 2q11.1-2q11.2 (chr2:96780257-97833468), which is shared by the two siblings and which was confirmed by using the Affymetrix GeneChip SNP6.0 Array for both the siblings (Additional file 1: Figure S63). By interrogating the ISCA database [40], we found recurrent rearrangements involving this region and indicated as pathogenic in cases with developmental delay. Seven ISCA deletions had a 87% to 100% overlap with those found by EXCAVATOR and six of them were reported to be associated with ID, autism or general developmental delay, with both a *de novo *origin and parental inheritance and different pathogenetic roles (Additional file 6: Table S5). Interestingly, the same genomic region (chr2:96726273-97676273) was found at a very low frequency in cases affected by developmental delay (2/15,767), while it never occurred in controls (0/8,329) [41].

Within this deleted region, 21 NCBI RefSeq genes (*ADRA2B*, *ANKRD23*, *ANKRD36*, *ANKRD39*, *ARID5A*, *ASTL*, *WDR39*, *CNNM4*, *CNNM3*, *DUSP2*, *FAHD2B*, *FAM178B*, *FER1L5*, *ITPRIPL1*, *KANSL*, *LMAN2L*, *NCAPH*, *SEMA4C*, *SNRNP200*, *STARD7* and *TMEM127*) have been mapped. Moreover, 13 genes are recorded in the On-line Mendelian Inheritance in Man (OMIM) [42] catalog, some of which are associated with congenital disorders distinct from ID. Other genes are putative candidates to be defective in ID or neurodevelopmental delay: *ADRA2B* (alpha-2B-adrenergic receptor, MIM 104260) is one of the three highly homologous alpha-2-adrenergic receptors having a critical role in regulating neurotransmitter release from sympathetic nerves and from adrenergic neurons in the central nervous system and *ARID5A* (AT-rich interaction domain-containing protein 5A, MIM 61153) is a member of the ARID protein family, which might play important roles in development.

Overall, the detection of a recurrent 2q11.1-2q11.2 deletion in the two siblings affected by ID, demonstrated that EXCAVATOR is a suitable tool for widely screening the exomes of ID patients even for low-frequency CNVs. It has added a piece of information that possibly implicates this genomic region in producing susceptibility to neurocognitive defects.

Finally, we used the two ID samples to compare the performance of our pipeline with that of the methods mentioned in the Background section (see Materials and methods for analysis settings). Tests are described in the Population data analysis section. CoNIFER and XHMM were not able to identify any genomic regions involved in CNVs, thus confirming their limitations in analyzing small datasets comprising few samples. On the other hand, ExomeCNV detected 200 Mb (269 CNVs ranging from 36 Mb to 1 kb) and 342 Mb (245 CNVs ranging from 40 Mb to 1 kb) of genomic regions involved in CNV for the ID1 and ID2 samples, respectively. As discussed above, these results can be ascribed to the discrepancy in the total sequence read count between the case and control samples. Taken as a whole, these results show the uniqueness of our tool in the analysis of WES data for diagnosis.

### Effect of mapping algorithms and read length on EXCAVATOR performance

To investigate the effects of alignment tools and read lengths on the global performance of our computational pipeline, we analyzed the WES data for four individuals (NA10847, NA19131, NA19152 and NA19153) generated by the 1000 Genomes Project Consortium. To study the dependence of the outcome from EXCAVATOR on different short read aligners, we mapped reads using three of the most popular and commonly used algorithms (BWA [39], Bowtie2 [43] and SOAP2 [44]), while to evaluate the effect of read length we cut the original 100-nucleotide-long paired-end reads of the four samples into 75-nucleotide-long and 50-nucleotide-long reads and compared the outputs (see Materials and methods for more details). Raw sequencing data were aligned to the human reference genome (hg19) and then subjected to a post-processing pipeline including Picard [45], SAMtools [46] and the Genome Analysis ToolKit [47] (see Materials and methods for more details). After the mapping pipeline, for each aligner and read length, we applied EXCAVATOR to the three samples, NA19131, NA19152 and NA19153, using NA10847 as control. First, we compared raw read count values for different aligners and read lengths. The comparison was performed by calculating the Pearson correlation coefficient between the read count values of each combination of aligner and read length. The results of these analyses are reported in Additional file 1: Figure S64 and show that using different aligners with different read lengths slightly affects the total number of reads mapped at each exon of the genome. For all read lengths investigated, Bowtie2 and BWA obtained a correlation coefficient greater than 0.99. SOAP2aligner had a smaller correlation coefficient than the other two algorithms, nevertheless it was larger than 0.98 for all examined cases. To evaluate the effect of read length and mapping algorithm on the ability of EMRC data to predict the exact DNA copy number values of a genomic region, we examined several broad genomic regions previously reported to have copy numbers equal to 0, 1, 2, 3, 4, 5 or 6 by McCarroll *et al.*[7]. We calculated the correlation between the EMRC ratio and the absolute DNA copies predicted by McCarroll *et al.*[7]. The results of these analyses are reported in Additional file 1: Figure S65 and show that the prediction of the absolute number of DNA copies is independent of the read length and mapping algorithm: in all analyses we obtained a Pearson correlation coefficient between 0.77 and 0.79.

## Conclusions

In this work we present a novel computational method based on the RC approach to detect CNV regions starting from whole-exome sequencing data. We studied the statistical properties and systematic biases of RC targeted sequencing data and introduced a novel normalization procedure to mitigate the effects of these biases. We also demonstrated the capability of such normalized WES data to predict the exact number of DNA copies for CNV regions.

Furthermore, we developed a novel heterogeneous hidden markov model based algorithm (HSLM), which exploits the sparseness of coding regions throughout the genome to detect both small isolated events and large alterations. Testing HSLM on synthetic data showed that it was able to detect, with a comparable accuracy, large genomic regions covered by many exons as well as small genomic regions covered by few exons. Moreover, synthetic simulations were also exploited to compare the performance of HSLM to the CBS algorithm. Our results show that HSLM outperforms CBS in both sensitivity and specificity, thus improving our ability to identify small and highly isolated CNV regions covered by few exons. Also, we extended a method previously developed for array-CGH analysis to classify genomic regions obtained from HSLM segmentation into discrete copy number states. Finally, we packaged all these algorithms into a novel software tool named EXCAVATOR.

To demonstrate the usefulness and versatility of our tool in analyzing different experimental designs, we applied our computational pipeline to three WES datasets generated using different exome capture and sequencing technologies and we compared its performance with three recently published methods for CNV calling from WES data (ExomeCNV, CoNIFER and XHMM).

To show the potential of EXCAVATOR in population genetics studies, we analyzed 20 healthy individuals sequenced by the 1000 Genomes Project Consortium and previously genotyped with microarray technologies. Our method detected both rare and common variants and the comparison with known CNVs from microarray studies show that EXCAVATOR outperforms the other three pipelines in both precision and recall.

We tested our tool to see if it applicable to cancer genomics studies by using it to identify genomic alterations in six metastatic melanoma cell lines. The results were compared with those obtained by SNP array analysis. We found considerable concordance between WES and SNP array results, which show that WES data have much greater sensitivity and a wider dynamic range than SNP array data for detecting deletions and amplifications. A comparison with a tool developed and calibrated for cancer data analysis (ExomeCNV), demonstrated that EXCAVATOR had better performance for both sensitivity and specificity.

Finally, we studied genomic alterations in two siblings affected by intellectual disability. Our tool detected a large deletion on chromosome 2, which was confirmed by SNP array analysis for both samples and suggested that there is potential pathogenic interest for this disease. None of the other methods performed as well as EXCAVATOR.

All of the comparative analyses we performed highlighted the versatility of our software and its ability to overcome the limitations and drawbacks of currently available state-of-the-art tools. Importantly, while the other software packages are limited to three classification states, EXCAVATOR can quantify and discriminate five copy number states, thus allowing it to distinguish one-copy from two-copy deletions and one-copy duplications from multiple-copy amplifications. Moreover, we found that ExomeCNV generates a huge number of false positive events while CoNIFER and XHMM produce a significant number of false negatives. These results are mainly ascribed to the different normalization procedures implemented in the three software packages: ExomeCNV does not take into account the discrepancy in the total sequence read count between the case and control samples, while CoNIFER and XHMM analyze many samples simultaneously to remove systematic noise. The computational pipeline we presented in this paper can be run on single samples and the results are not affected by dataset size, thus making EXCAVATOR a suitable tool for the investigation of CNVs in large-scale projects (such as the 1000 Genomes Project and the Cancer Genome Atlas) as well as in clinical research and diagnosis.

## Materials and methods

### GC content and mappability

To calculate the GC content percentage for each exon we used the gc5Base tracks downloaded from the UCSC website [48]. gc5Base tracks give the percentage of G (guanine) and C (cytosine) bases in five-base windows. Mappability bias is due to the fact that the genome contains many repetitive elements and aligning reads to these positions leads to ambiguous mapping. We used the uniqueome data in [49] to calculate a mappability score for each exon. In this paper, the authors introduced a genomic resource to understand the uniquely mappable proportion of genomic sequences. We evaluated the uniqueness of genomic sequences using an all-against-all alignment for different word sizes. Alignments were performed with the Imagenix Sequence Alignment System (ISAS) [50]. The all-against-all alignments were performed independently for tag lengths between 25 and 90 nucleotides with varying numbers of mismatches, in both nucleotide space and color space. The results of these analysis were formatted as bigBED and bigWig files and can be downloaded from [51]. The bigWig files contain coverage values expressed as rounded integer percentiles of full coverage (for example, a value of 100 indicates that 100% of overlapping N-mers are unique and contribute to coverage of that coordinate; similarly a value of 50 indicates that 50% of overlapping N-mers are unique). A mappability score for each exon was obtained by averaging the coverage values of the nucleotides belonging to the selected exon.

### Exon mean read count data normalization

To minimize the effect of the three sources of variation, we used a three-step bias removal procedure based on the median normalization approach introduced in [23] and in [31]. In practice, for all of the GC percentages (0,1,2,…,100*%*), all of the bin of mappability scores (0,0.1,0.2,…,1) and all of the bin of exon sizes (10 bp, 20 bp, 30 bp, …) we calculated the deviation of EMRC from the exome average and then corrected each EMRC according to:

(3) $$\bar{{\text{EMRC}}_{i}}={\text{EMRC}}_{i}\cdot \frac{m}{m_{X}},$$

where EMRC_*i *_is the exon mean read count of the *i*th exon, *m*_X _is the median EMRC of all the exons that have the same X value (where X = [GC content, mappability score, exon size]) as the *i*th exon, and *m *is the overall median of all the exons. At the end of this procedure, the EMRC for each exon has been corrected for the three sources of bias.

### Copy number estimation

To measure the ability of EMRC data to predict the exact DNA copy number of a genomic region, we examined several broad genomic regions that were previously reported to have copy numbers equal to 0, 1, 2, 3 or 4 by McCarroll *et al.*[7] for the eight samples (NA10847, NA19131, NA19138, NA19152, NA19153, NA19159, NA19206 and NA19223) generated by the 1000 Genomes Project Consortium. McCarroll *et al.*[7] designed a hybrid genotyping array (Affymetrix SNP 6.0) to measure 906,600 SNPs and copy numbers at 1.8 million genomic locations simultaneously. They used this array to develop a high-resolution map of copy number variation for 270 HapMap samples. Their goal was to construct a map that was precise and accurate for the boundaries of the genomic regions affected by CNV and to determine an accurate integer copy number level for each segment in each individual. The boundaries of each CNV were determined using a hidden Markov model and the integer copy number level was estimated using quantitative PCR. For samples NA19152, NA19159, NA19131, NA19153, NA19138, NA19223, NA19206 and NA10847 they detected 191, 193, 183, 173, 172, 202, 185 and 148 CNV regions, respectively, with copy numbers equal to 0, 1, 3 or 4. The table of DNA copy numbers estimated in [7] were downloaded from the Nature Genetics website. The results shown in Figure 1i,g were obtained using the EMRC data median normalized to copy number 2 of the seven samples of Yoruba ancestry for genomic regions, while the results reported in Figure 1h,j were obtained using the EMRC ratio between the seven samples of Yoruba ancestry and the NA10847 sample for these genomic regions. To evaluate the linear relation between RC and CNV regions we calculated the Pearson correlation coefficient.

### Calling algorithm

To classify each segmented region as one of five discrete copy number states (two-copy deletion, one-copy deletion, normal, one-copy duplication or multiple-copy amplification) we used the FastCall algorithm [36], which we developed to classify array-CGH data. The FastCall calling procedure is a mixture model based algorithm, which can be used to classify each segmented region as one of five predefined copy number states: double loss, loss, neutral, gain or multiple gain. Our calling procedure models the mean of each segment as a mixture of five truncated normal distributions and can also take into account sample heterogeneity using a cellularity parameter *c* (see Additional file 1 for more details). The algorithm takes as input the mean level of each segment *m *= (*m*_1_,*m*_2_,…,*m*_*i*_,…,*m*_*N*_), identified by the HSLM algorithm and gives as output the probability that a segment (mean) belongs to a particular state.

### EXCAVATOR tool

**Figure 5:**
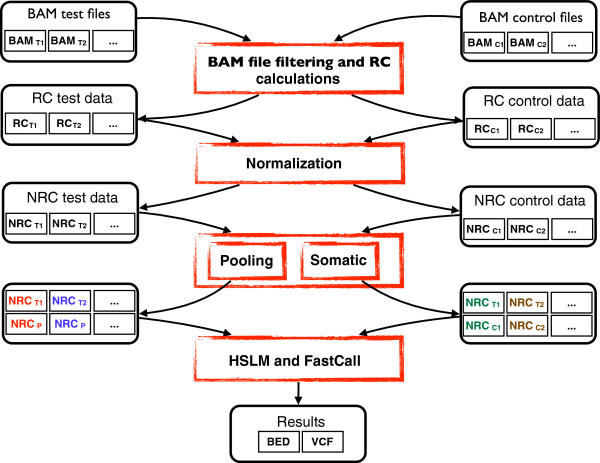
**EXCAVATOR workflow.** BAM files of both test and control samples are processed by means of SAMtools and R scripts for EMRC calculations. After EMRC calculation, EXCAVATOR corrects the data for GC-content, mappability and exon size. After normalization, normalized read count (NRC) for each sample are organized according to the analysis mode (pooling or somatic) selected by the user: pooling mode to compare one sample to a pool of normal controls, somatic mode to compare one sample to its corresponding normal control. Finally, HLSM and FastCall are applied to normalized data and results are provided as tab-delimited text files (variant call format, VCF and BED format). HSLM, heterogeneous shifting level model; RC, read count.

All the algorithms and methods here described have been packaged in the EXCAVATOR software. EXCAVATOR is a collection of Perl, Bash, R and Fortran codes. Figure 5 is a schematic representation of EXCAVATOR’s workflow steps. It takes as input BAM files and gives as output figures for raw and normalized data, plots of segmentation and calling results and a list of detected CNVs as tab-delimited text files. The package can analyze samples with two different experimental designs: ‘pooling’ and ‘somatic’. In the pooling scheme, each test sample is compared with a pooled reference obtained by summing the total number of reads for each exon across all the control samples. In the somatic scheme, each test sample is compared with its matched control. The EXCAVATOR tool can run on any UNIX system (desktops and workstations). On a desktop computer with a 2.5-GHz CPU and 8 GB of RAM, it takes four hours to analyze ten WES samples sequenced at 60 ×. The EXCAVATOR tool is freely available from [52].

### Population dataset

The genomes of all 27 individuals were sequenced by the 1000 Genomes Project Consortium and data were downloaded from [53] as BAM files. The data were first filtered and normalized as reported in Additional file 1 and then analyzed using HSLM followed by the FastCall algorithm with default parameters (see Additional file 1 for more details).

### Melanoma dataset

For the melanoma dataset, all tumor and normal samples were captured using the same target enrichment kit (Agilent SureSelect Human All Exon 50 Mb kit) and sequenced, one sample per lane, in a 76-bp paired-end GAIIx run, thus obtaining a mean depth on the target of 43 × (range 32 × to 54 ×) (see Table 1 and Additional file 1: Table S3). Exome sequencing data are available at the Sequence Read Archive under accession ERP001844. WES reads of the 12 samples were aligned against the human reference genome hg19 by means of the BWA aligner, then filtered, normalized and analyzed by the HSLM and FastCall algorithms with default parameters (see Additional file  1). Since we did not have autologous normal samples for matched controls, WES reads from the six normal blood samples were pooled and used as a common reference baseline (see Additional file 1).

The same 12 samples were profiled using the Affymetrix 250K SNP Array platform and signal intensities were acquired by the GCOS software and normalized with the CNAG software. Melanoma cell line data were compared to the common reference pool composed of the six normal blood samples. The normalized log2 ratio SNP copy number values generated for each tumor sample were segmented using the SLM segmentation algorithm and the FastCall calling procedure was used to classify all the segmented genomic regions into defined copy number states (see Additional file 1).

### Intellectual disability dataset

The two ID samples were captured using the same Illumina Truseq Target Enrichment kit and sequenced as 100-bp paired-end reads with a mean base coverage of 63 × using the Illumina HiSeq2000 platform (see Table 1 and Additional file 1: Table S4). Exome sequencing data are available at the Sequence Read Archive under accession ERP001831. The WES data of the healthy individual of European descent sequenced by [39] were generated by the same exome-capture and sequencing platform used for the two ID samples (Illumina Truseq Target Enrichment kit and the Illumina HiSeq2000 platform). Reads from the three samples were aligned against the human reference genome hg19 by the BWA aligner, then filtered, normalized and analyzed by the HSLM and FastCall algorithms with default parameters (see Additional file 1).

### Algorithm comparison

We compared our algorithm to three previously published software packages: ExomeCNV [25], CoNIFER [26] and XHMM [27]. We downloaded the ExomeCNV R package version 1.4 from [54]. We used ExomeCNV with default parameters: sensitivity and specificity were set at 0.9999 for exons (maximizing specificity) and 0.99 for calls (‘auc’ option), and the admixture rate was set at a value of 0.5 (although all the samples used in this work had no biological admixture, we found that this setting reduced the number of false positive calls). We downloaded CoNIFER 0.2.2 from [55]. After running the analysis with the − − *plot_screen *option, we examined the components plot and we decided to run the final CoNIFER analyses with the setting to remove two singular value decomposition components (− − *svd *2). XHMM was downloaded from [56]. The XHMM tool was applied to the three datasets using the default parameter setting and following the instructions on [57].

### Alignment algorithms and read trimming

Raw reads in fastq format were downloaded from [58] for each of the four samples (NA10847, NA19131, NA19152 and NA19153). As a first step, the original 100-nucleotide reads were trimmed to 75 nucleotides and 50 nucleotides using the fastx-trimmer of the FASTX Toolkit 0.0.13.1 [59], then, raw reads were aligned to the human reference genome (hg19) using BWA, Bowtie2 and SOAP2 with default parameter settings. We downloaded BWA version 0.6.1-r104 from [60], Bowtie2 version 2.1.0 from [61] and SOAPaligner version 2.21 from [62]. The output from SOAP2aligner was converted into sequence alignment map (SAM) format exploiting the Perl soap2sam.pl script (available from [62]). SAM files were processed using Picard [45], SAMtools [63] and the Genome Analysis ToolKit (GATK) (3,4) release 2.5-2 [64]. In brief, SAM files were binary compressed, sorted and indexed by SAMtools (samtools view, sort and index tools), duplicated reads were removed by Picard (with MarkDuplicates) and base quality score recalibration and local realignment around indels followed the recommended workflow of the GATK toolkit (RealignerTargetCreator, IndelRealigner, BaseRecalibrator and PrintReads).

## Abbreviations

BP: Base pair; CBS: Circular binary segmentation; CGH: Comparative genomic hybridization; CNA: Copy number alteration; CNV: Copy number variant; DGV: Database of genomic variants; EMRC: Exon mean read count; FP: False positive; FPR: False positive rate; Gb: Gigabase; HSLM: Heterogeneous shifting level model; HTS: High-throughput sequencing; ID: Intellectual disability; Kb: Kilobase; Mb: Megabase; PCA: Principal-component analysis; PCR: Polymerase chain reaction; RC: Read count; ROC: Receiver operating characteristic; SAM: Sequence alignment map; SLM: Shifting level model; SNV: Single nucleotide variant; SVD: Singular value decomposition; TP: True positive; TPR: True positive rate; WES: Whole-exome sequencing.

## Competing interests

The authors declare that they have no competing interests.

## Authors’ contributions

AM conceived and designed the basic algorithm for EXCAVATOR. LT implemented and optimized the package. IC, CB and EM conducted the melanoma dataset experiments. PM, EB and TP ran the intellectual disability experiments. AM, LT and RD carried out the comparison of the different tools. AK, BG, GDB, RA, GFG, GR and MS supervised the project and gave advice. AM, LT, IC and TP wrote the manuscript. GR, GDB, BG and MB revised the manuscript. All authors read and approved the final manuscript.

## Supplementary Material

Additional file 1 **Supplemental methods.** Supplemental methods for EXCAVATOR: detecting copy number variants from whole-exome sequencing data. Click here for file

Additional file 2: Table S1 The complete list of CNVs detected by EXCAVATOR on chromosomes 1 and 4 of the population dataset. Click here for file

Additional file 3: Table S2 The complete list of CNAs detected by EXCAVATOR on the WES data of the melanoma dataset. Click here for file

Additional file 4: Table S3 The complete list of CNAs detected by SLM segmentation algorithm on the SNP array data of the melanoma dataset. Click here for file

Additional file 5: Table S4 Complete list of CNVs detected by EXCAVATOR on the WES data of the ID dataset. Click here for file

Additional file 6: Table S5 List of the seven ISCA deletions that had a 87% to 100% overlap with the large deletion that we found in our ID samples. Click here for file
