# Supplementary material for: EXCAVATOR: detecting copy number variants from whole-exome sequencing data
Source: Genome Biol. 2013 Oct 30;14(10):R120. doi: 10.1186/gb-2013-14-10-r120 (PMC4053953; doi:10.1186/gb-2013-14-10-r120)
Supplement: Additional file 1 — Supplemental methods. Supplemental methods for EXCAVATOR: detecting copy number variants from whole-exome sequencing data. [file gb-2013-14-10-r120-S1.pdf]

Supplemental Methods to "EXCAVATOR: detecting copy number variants from whole-exome sequencing data".

Alberto Magi[1,*,†], Lorenzo Tattini[2,†], Ingrid Cifola[3], Romina D'Aurizio[4], Matteo Benelli[6], Eleonora Mangano[3], Cristina Battaglia[3,7], Elena Bonora[5], Ants Kurg[8], Marco Seri[5], Pamela Magini[5], Betti Giusti[1], Giovanni Romeo[5], Tommaso Pippucci[5], Gianluca De Bellis[3], Rosanna Abbate[1] and Gian Franco Gensini [1].

[1]Department of Clinical and Experimental Medicine, University of Florence, Florence, Italy, [(]2)Laboratory of Molecular Genetics, G. Gaslini Institute, Genova, Italy [3]Institute for Biomedical Technologies, National Research Council, Segrate, Milano, Italy. [4]Laboratory of Integrative Systems Medicine (LISM), Institute of Informatics and Telematics and Institute of Clinical Physiology, National Research Council, Pisa, Italy , [5]Medical Genetics Unit, Department of Medical and Surgical Sciences, University of Bologna, Bologna, Italy [6]Diagnostic Genetic Unit, Careggi Hospital, Florence, Italy, [7]Dipartimento di Biotecnologie Mediche e Medicina Traslazionale (BIOMETRA), University of Milan, Milan, Italy, [8]Institute of Molecular and Cell Biology, University of Tartu, Tartu, Estonia. [†]These authors contributed equally to this work.

Email:

*Corresponding author

Supplemental Methods

Sequencing data alignment and filtering

To align all the sequencing data used in this study, with the exception of the 1000 Genomes Project samples, we used the BWA short read aligner 0.6.2 (Li *et al.*, 2010). BWA is a very fast tool that aligns relatively short sequences to a sequence database, such as the human reference genome. The BWA tool implements an alignment algorithm based on the Burrows-Wheeler Transform (BWT) that allows for gapped global alignment and supports paired-end reads. To align the reads against the human reference genome (hg19) we used the *aln* command, while to generate alignments in the SAM format given paired-end reads we used the *sampe* command.

To mitigate the effects of PCR amplification bias introduced during library preparation, we removed duplicated reads in all the samples we analyzed. Duplicate removal was accomplished by the Picard package ($http://picard.sourceforge.net/$) that comprises Java-based command-line utilities that manipulate SAM files, and a Java API (SAM-JDK) for creating new programs that read and write SAM files. The MarkDuplicates command of Picard allows to remove potential PCR duplicates: if multiple read pairs have identical external coordinates, it retains only the pair with the highest mapping quality.

The BWA aligner, like the great majority of aligner tools, such as MAQ (Li *et al.*, 2008), SSAHA (Ning *et al.*, 2001), BOWTIE (Langmead *et al.*, 2009) and BFAST (Homer *et al.*, 2009) allows to produce a mapping quality (MQ) score for each read aligned to the reference genome. The mapping quality of a read alignment Q is related to the probability P that the alignment is wrong according to the following equation:

$$Q = -10 \cdot \log_{10} P. \tag{1}$$

The calculation of mapping qualities takes into account the repeat structure of the reference genome, the base quality of each read and the sensitivity of the alignment algorithm. In this way, reads falling in repetitive regions of the reference genome or with low base quality usually get very low MQ. When a read has $MQ = 0$, it means that there are at least two regions of the genomes that perfectly match that read. Conversely, when a read has $MQ = 30$, it means that the best alignment has few mismatches and that the read has few or just one "good" hit on the reference. For all the alignments data we used in

this work, reads with mapping quality $MQ \leq 10$ were removed by using the SAMtools package (Li *et al.*, 2009).

Read Count on targeted regions

Read Count (RC) approach (Magi *et al.*, 2012) is based on the assumption that if the sequencing process is uniform then the number of reads mapping to each genomic region is expected to be proportional to the number of times the region appears in the DNA sample. Following this assumption, the copy number of any genomic region can be estimated by counting the number of reads aligned to non-overlapping and contiguous genomic windows of predefined size L. This approach is unsuitable for non-contiguous targeted sequencing data, such as WES data, since predefined genomic windows can not be defined. In order to study DNA copy number variations from targeted sequencing data, we choose to use the mean number of reads aligned to each exon (exon mean read count, EMRC). EMRC is defined in the following:

$$EMRC_e = \frac{RC_e}{L_e} \tag{2}$$

where RC_e is the number of reads aligned to a targeted genomic region e and L_e is the size of that same genomic region (in bp). EMRC is calculated for each targeted region of the genome and gives a measure of the density of reads aligned to that particular region.

Data Biases and correction

RCs data have been demonstrated to be affected by two main sources of bias, namely the local GC content and the genomic mappability (Magi *et al.*, 2012). The correlation between RC (or depth of coverage) and DNA GC content has been reported in several papers: Harismendy *et al.* (2009) analysed 260 kb of targeted regions sequenced by three different HTS platforms (Roche 454, Illumina GA and Life Technologies SOLiD) concluding that read depth of coverage decreases with increasing AT content, while in a recent paper we observed that RC is maximum for GC content values ranging between 35% and 60%, while it decreases at both extremes (Magi *et al.*, 2012). Mappability bias (Miller *et al.*, 2011) is due to the fact that human genome contains many repetitive elements which may cause ambiguous mapping when aligning reads to these positions. In Magi *et al.* (2012) we found a strong correlation between RC data and genome mappability and we showed that for high mappability scores, RC distribution was closer to Poissonian than genomic regions with low mappability (these showing large RC overdispersion). In this work we studied the correlation between EMRC data and three sources of bias: the local GC content, the genomic mappability and the exon size. Moreover, in order to minimize the effect of these sources of variation and make data comparable within and between samples, we implemented a three-step bias removal procedure based on the median normalization approach introduced by Yoon *et al.* (2009) for the removal of the GC-content effect and then extended by us (Magi *et al.*, 2012) for removing mappability bias. In practice, for all the GC percentages (0, 1, 2,..,100%), all the bin of mappability score (0, 0.1, 0.2,...,1) and all the bin of exon size (10 bp, 20 bp, 30 bp,...), we calculated the deviation of EMRC from the exome average and then corrected each EMRC according to the following formula:

$$\overline{EMRC_e} = EMRC_e \cdot \frac{m}{m_{\mathrm{X}}}, \tag{3}$$

where $EMRC_e$ are the exon mean read counts of the e-th exons, m_{X} is the median $EMRC$ of all the exons that have the same X value (where X=(GC content, mappability score, exon size)) as the i-th exon, and m is the overall median of all the exons. At the end of this procedure, the EMRC for each exon results corrected for the three aforementioned sources of bias.

Heterogeneous Shifting Level Model

In classical Shifting Level Model (SLM) (Magi *et al.*, 2010), sequential observations $x = (x_1, .., x_i, .., x_N)$ are considered to be realizations of the sum of two independent stochastic processes:

$$x_i = m_i + \epsilon_i, \tag{4}$$

$$m_i = (1 - z_{i-1}) \cdot m_{i-1} + z_{i-1} \cdot (\mu + \delta_i). \tag{5}$$

where m_i is the unobserved mean level that follows a normal distribution with mean μ and variance σ^2_μ ($m_i \sim N(\mu, \sigma^2_\mu)$) and ϵ_i is a normally distributed white noise with variance σ^2_ϵ ($\epsilon_i \sim N(0, \sigma^2_\epsilon)$).
The process m_i changes its value independently of m_{i-1} and is controlled by the process z_i : when $z_{i-1} = 0$, m_i is the same as m_{i-1} and when $z_{i-1} = 1$, m_i is incremented by the normal random variable δ_i ($\delta_i \sim N(0, \sigma^2_\mu)$). $z_1, z_2, ...$ are independent and identically distributed random variables taking the values 0,1 with probabilities $\eta = Pr(z_i = 1)$, $1 - \eta = Pr(z_i = 0)$. As reported in the main manuscript, in order to take into account genomic distance between adjacent coding regions of the genome we incorporated the genomic distance in the transition matrix of the SLM by defining the probability $Pr(z_i = 1)$ in the following:

$$Pr(z_i = 1) = \eta(d_i) = \frac{1}{2} \cdot \theta + ((\frac{1}{2} - \theta) \cdot \exp\left[\frac{log(\theta)}{\frac{d_i}{d_{Norm}}}\right]) \tag{6}$$

where $\eta(d_i)$ is the probability of random variables z_i to be equal to 1, θ is a constant parameter , d_i is is the distance between the $i - th$ and $i - 1 - th$ targeted region and d_{Norm} is the distance normalization parameter. Equation 6 defines the dependence between the probability $Pr(z_i = 1)$ and the genomic distance between adjacent exons d_i: the larger is the genomic distance and the larger is $Pr(z_i = 1)$ and consequently the larger is the probability to jump between two mean levels m_i. The constant parameter θ can be seen as the baseline probability of random variables z_i to take value 1 while the d_{Norm} parameter modulates the genomic distance at which the probability $Pr(z_i = 1)$ begins to grow: for distances smaller than d_{Norm} the probability $Pr(z_i = 1) = (\theta - \theta^2) \sim \theta$, while when d_i is larger than d_{Norm} the probability $Pr(z_i = 1)$ grows until reaching the value 1.

The expected value of x_i is equal to μ and, since the two stochastic processes are independent, the variance of x_i is the sum of the variances of the two processes:

$$E[x_i] = \mu, \tag{7}$$

$$var[x_i] = \sigma^2_\mu + \sigma^2_\epsilon. \tag{8}$$

In this way, SLM allows one to break up the total variance of the genomic profile in two parts: the biological variance (σ^2_μ) and the experimental variance (σ^2_ϵ). Using (8) we can introduce a different parametrization of the SLM by defining the parameter $\omega = \sigma^2_\mu/\sigma^2$ (with $\sigma^2 = var[x_i]$) such that σ^2_μ by $\omega \cdot \sigma^2$ and σ^2_ϵ by $(1 - \omega)\sigma^2$.
With this new parametrization and by using equation (3), the joint probability distribution of the observations and latent variables $p(x, m, z|\Theta)$, given the parameters, can be explicated in the following:

$$\begin{aligned} &p(x, m, z|\theta) = p(x|m, \sigma^2, \omega) \cdot p(m|z, \mu, \sigma^2, \omega) \cdot p(z|\eta(d_i)) = \\ &= \textstyle\prod_{i=1}^N p(x_i|m_i, \sigma^2, \omega) \cdot p(m_0) \times \prod_{i=0}^N p(m_{i+1}|m_i, z_i, \mu, \sigma^2, \omega) \cdot p(z_i|\eta(d_i)), \end{aligned} \tag{9}$$

Equation 9 defines an Heterogeneous Hidden Markov Model (HHMM) of order one, in which a single state variable, $q_i = (m_i, z_i)$, summarizes all the relevant past information of the underlying process.
In the model defined by (9) the elements of the HHMM are the following:

- the state transition probability distribution is:
  $p(q_{i+1}|q_i, \theta) = p(m_{i+1}|m_i, z_i, \mu, \sigma^2, \omega) \cdot p(z_i|\eta(d_i))$
- the emission probability distribution is:
  $p(x_i|q_i, \theta) = p(x_i|m_i, \sigma^2, \omega)$
- the initial state probability distribution is:
  $p(q_0|\theta) = p(m_0|\mu, \sigma^2, \omega)$

Heterogeneous Shifting Level Model Algorithm

The fact that SLM is an HMM allows us to make use of the several algorithms developed for these kinds of models. To handle the infinite dimensionality of SLM, we use the same approach we previously developed

in (Magi *et al.*, 2010).
We introduce a Markovian stochastic process $s_1, s_2, ..., s_k$ taking values in $S = \{1, 2, ..., K\}$. From equation 9, we know that the probability distribution of x_i, given the parameters, has the following form:

$$p(x_i|m_i, \sigma^2, \omega) = N(x_i|m_i, (1-\omega)\cdot\sigma^2), \tag{10}$$

hence we assume that the conditional probability of x_i, given $s_i = k$, is $N(\mu_k, \sigma_\epsilon^2)$. The parameter $\mu_k (k = 1, 2, ..., K)$ is associated to each state of the Markovian stochastic process and represents an approximation of the m_i latent variables of the SLM.
The emission function of the HMM is defined as follows:

$$f_k(x) = \frac{1}{\sqrt{2\pi}\sigma_\epsilon}\exp\left[-\frac{1}{2}\left(\frac{x-\mu_k}{\sigma_\epsilon}\right)^2\right]. \tag{11}$$

To complete the description of the model, it remains to specify the state transition matrix P. From (9) the state transition probability has the following form:

$$\begin{aligned} p(m_{i+1}|m_i, z_i, \mu, \sigma^2, \omega)\cdot p(z_i|\eta(d_i)) \quad = \quad & [(1-z_i)\cdot\delta(m_{i+1}-m_i) + z_i\cdot N(m_{i+1}|\mu, \omega\cdot\sigma^2)]\cdot \\ & \cdot[\eta(d_i)\cdot\delta(z_i-1) + (1-\eta(d_i))\cdot\delta(z_i)], \end{aligned} \tag{12}$$

hence the state transition matrix is:

$$P_{jk} = \begin{cases} (1-\eta(d_i)) + \eta(d_i)\cdot g_{jk} & j = k \\ \eta(d_i)\cdot g_{jk} & j \neq k \end{cases} \tag{13}$$

where

$$\begin{aligned} g_{jk} &= c_j \cdot e^{-\frac{(\mu_k-\mu)^2}{2\sigma_\mu^2}}, \\ c_j &= \left(\sum_{k=1}^{K} e^{-\frac{(\mu_k-\mu)^2}{2\sigma_\mu^2}}\right)^{-1}. \end{aligned} \tag{14}$$

To estimate the parameters of the Heterogeneous Shifting Level Model (HSLM), we develop a two-step algorithm that follows our previous idea in Magi *et al.* (2010). Since the log2-EMRC-Ratio can take values in a well-defined range, we used a large number of states K0 and we choose μ_k in order to densely and homogeneously cover the range [-h,h] instead of estimating the μ_k parameters by using the Baum and Welch algorithm (Magi *et al.*, 2010).

This simple solution drastically improves the computational performance of our algorithm without affecting its accuracy in the detection of signal shifts.
In the first step of the algorithm we estimate the mean μ and the variances σ^2, σ_μ^2 and σ_ϵ^2 with the following formulas:

$$\begin{aligned} &\mu = \frac{\sum_{i=1}^{N} x_i}{N}, \\ &\sigma = \sqrt{\frac{\sum_{i=1}^{N}(x_i-\mu)^2}{(N-1)}}, \\ &\sigma_\mu^2 = \omega\cdot\sigma^2, \\ &\sigma_\epsilon^2 = (1-\omega)\cdot\sigma^2. \end{aligned} \tag{15}$$

In the second step we apply the Viterbi algorithm to find the best state sequence $s^{(j)}$ and estimate the points of mean shift z_i. After Viterbi algorithm we calculate the median of the data that belong to each segment. The inputs to the algorithm are the sequence $x = (x_1, .., x_i, .., x_N)$ to be segmented, the distance between adjacent exons $d = (d_1, ..., d_i, ..., d_N)$, the number of states $K^{(0)}$, the parameter ω, the parameter θ, and the distance normalization parameter d_{Norm}.
The meaning of the parameters θ and ω is the same we previously stated in Magi *et al.* (2011). The parameter ω modulates the proportionality between the white noise stochastic process (σ_ϵ^2) and the means jump stochastic process (σ_μ^2). For large values of ω (small σ_ϵ and large σ_μ) the algorithm sees the signal to have small white noise and takes as level shift also slight variations of the genomic profile. On the

contrary when ω is small a large fraction of the total variance σ^2 is assigned to the σ_ϵ (σ_ϵ is large and σ_μ is small) and only sizeable variations of the signal are taken as level shift. The parameter θ corresponds to the baseline probability that a transition to a new mean level occurs at any position i of the sequential process and is able to control only specificity and has weak effect on sensitivity. The parameter $K^{(0)}$ regulates the density of the μ_k in the range [-h,h]: the larger is $K^{(0)}$ and the larger is the μ_k density in the interval [-h,h]. The use of predefined μ_k values in the range [-h,h] does not affect the results of the segmentation procedure. This is due to the fact that the Viterbi algorithm is able to correctly identify all the mean shifts (z_i) also when the μ_k value does not perfectly match with the real value of the segments. In fact, when a state m_i does not have its associated μ_k, the Viterbi algorithm associates m_i to the most lilkely μ_k, allowing for the identification of all the mean shifts z_i. Finally, the distance normalization parameter d_{Norm} modulates the ability of HSLM to detect both small and highly isolated coding regions of the genome and large and highly exons-covered genomic alterations.

FastCall Model

The FastCall calling procedure is a mixture model based algorithm that allows for the classification of each segmented region into five predefined copy number states: double loss, loss, neutral, gain and multiple gain. The algorithm takes as input the mean level of each segment $m = (m_1, m_2, ..., m_i, ..., m_N)$, identified by the HSLM algorithm and gives as output the probability that a segment (mean) belongs to a particular state. FastCall models the mean level of a segment as a mixture of five truncated normal distributions. The expression of a truncated Gaussian density g with lower bound l and upper bound u can be easily derived from the density of a non-truncated Gaussian:

$$g_l^u(m_i;\theta) = \frac{f(m_i;\theta)}{F(u;\theta) - F(l;\theta)} I_l^u(m_i), \tag{16}$$

where $f(\bullet;\theta)$ and $F(\bullet;\theta)$ represent the density and cumulative distribution functions of a non-truncated Gaussian of parameter $\theta = (\mu, \sigma^2)$ and $I_l^u(m_i) = 1$ if m_i belongs to the interval $[l, u]$ and $I_l^u(m_i) = 0$ otherwise. In this way the five state model has the following formula:

$$g_l^u(m_i;p,\theta) = \sum_{k=1}^{5} p_k g_l^u(m_i;\theta_k), \tag{17}$$

where p_k are the proportion of the k-th component in the mixture with $\sum_{k=1}^{5} p_k = 1$ and $\theta_k = (\mu_1, \mu_2, \mu_3, \mu_4, \mu_5, \sigma_1, \sigma_2, \sigma_3, \sigma_4, \sigma_5)$ are the means and the variances of the five gaussians.

To give biological meaning to the five normal distributions (double loss, loss, neutral, gain and multiple gain), we enforce the truncation intervals to follow the constraints reported in table 1. In particular, we impose the neutral state to belong to the interval $[-\epsilon_d, +\epsilon_u]$, the gain state to belong to the interval $[+\epsilon_u, \mu_4 + 3 \cdot \sigma_4]$ and the loss state to belong to the interval $[\mu_2 - 3 \cdot \sigma_2, -\epsilon_d]$. In this way we impose that the truncation bounds are not overlapped. The value of the truncation bound ϵ_d and ϵ_u can be set up in the main R function of FastCall. In all the analyses we performed in this paper we set ϵ_d to be equal to 0.5 and ϵ_u to be equal to 0.35. Finally, in order to obtain a higher variability of the mean level for aberrated states we impose that $\sigma_1 \geq \sigma_2 \geq \sigma_3$ and $\sigma_5 \geq \sigma_4 \geq \sigma_3$.

FastCall Algorithm

To estimate the parameters of the gaussian mixture model (17), we make use of the classical Expectation Maximization (EM) algorithm (Dempster *et al*, 1977). Denoting with Z_{ki} the hidden state for segment k (Z_{ki} is a random variable equal to 1 if segment i belongs to state k and 0 otherwise), we can define the conditional probabilities $\tau_{ki} = Pr(Z_{ki} = 1|m_i)$. The basic ingredient of the EM family of algorithms is the iterative application of an expectation step followed by a likelihood maximization step. EM starts with initial values ($p_k^{(0)}$, $\mu_k^{(0)}$, $\sigma_k^{(0)}$) for the parameters, and iteratively performs the two steps until convergence. In the E-step the conditional probabilities τ_{ki} are computed. Given the parameters estimated at h-th iteration, $p_k^{(h)}$ and $\theta_k^{(h)} = (\mu_k^{(h)}, \sigma_k^{(h)})$, the conditional probabilities τ_{ki}^{h+1} are obtained with the following formula:

$$\tau_{ki}^{h+1} = \frac{p_k^{(h)} g_l^u(m_i; \theta_k^h)}{\sum_{i=1}^N p_k g_l^u(m_i; \theta_l)}. \quad (18)$$

In the M-step, the proportions of the components in the mixture and the empirical estimators of the mean and the variance are computed. In particular at the iteration $(h + 1)$ of the M-step we compute $\mu_k^{(h+1)}$, $\sigma_k^{(h+1)}$ and $p_k^{(h+1)}$ with the following formulas:

$$\mu_k^{(h+1)} = \frac{\sum_{i=1}^N \tau_{ki}^{h+1} m_i}{\sum_{i=1}^N \tau_{ki}^{h+1}}, \quad (19)$$

$$\sigma_k^{(h+1)} = \frac{\sum_{i=1}^N \tau_{ki}^{h+1} (m_i - \mu_k^{(h+1)})^2}{\sum_{i=1}^N \tau_{ki}^{h+1}}, \quad (20)$$

$$p_k^{(h+1)} = \frac{\sum_{i=1}^N \tau_{ki}^{h+1}}{N}. \quad (21)$$

Classification Rules

In order to classify a segment k into defined copy number state, we use a posterior probability P_{ki}, $i = 1, ..., 5$, with the following rules:

- *Double Loss (0 Copies)* if $P_{k1} \geq 0.5$, marked by -2.
- *Loss (1 Copy)* if $P_{k2} \geq 0.5$, marked by -1.
- *Normal (2 Copies)* if $P_{k3} \geq 0.5$, marked by 0.
- *Duplication (3 Copies)* if $P_{k4} \geq 0.5$, marked by 1.
- *Multiple Copies (more than 3 Copies)* if $P_{k5} \geq 0.5$, marked by 2.

FastCall and sample heterogeneity

When analyzing cancer samples, it is well-known that tumor purity and sample heterogeneity could affect the mean CN values of altered DNA segments, thus hampering their correct detection and identification, while this problem does not exist when micro-dissection techniques are used to guarantee tumor purity of bioptic tissue samples. In order to take into account the fact that different proportion of tumor cells could impact the results of our genotyping algorithm we used the approach previously introduced by van de Wiel *et al* (2007). According to van de Wiel *et al* (2007), the signal S_N to be analyzed by FastCall is:

$$S_N = \log_2(\frac{R}{c} - \frac{1-c}{c}) \quad (22)$$

where R is the measured EMRC ratio between test and control samples and c is the cellularity parameter (fraction of tumor cells).

1000 Genomes Project Data

The 1000 Genomes Project (Durbin *et al.*, 2010) is the first project to sequence the genomes of a large number of people, to provide a comprehensive resource on human genetic variation. The plan for the full project is to sequence about 2,500 samples at 4X coverage. The goal of the 1000 Genomes Project is to find most genetic variants that have frequencies of at least 1% in the populations studied. An exome run is part of the whole-exome sequencing project which is targeting the CCDS gene set in 2500 individuals. The target of the whole-exome sequencing project can be downloaded at `ftp://ftp.1000genomes.ebi.ac.uk/vol1/ftp/technical/reference/exome_pull_down_targets/`. In this paper we used a total of 100 whole-exome samples sequenced by the 1000 Genomes Project Consortium. To study the properties of EMRC data distributions we used 8 samples (see Supplemental Table 2 for more details), including 7 samples of Yoruba ancestry (NA19138, NA19153, NA19206, NA19131, NA19223, NA19159, NA19152) and

one sample of Caucasian ancestry (NA10847). For the population genomic analysis we used 20 samples (Supplemental Table 2 for more details), comprising 7 CEU, Utah residents with ancestry from Northern and Western European (NA10847, NA11840, NA12249, NA12717, NA12751, NA12760, NA12761), 7 JPT, Japanese in Tokio (NA18966, NA18970, NA18967, NA18959, NA18973, NA18981, NA18999) and 6 YRI, Yoruba in Ibadan (NA19138, NA19153, NA19206, NA19131, NA19223, NA19159). As reported in the main text, in order to improve the performance of the normalization procedure of CoNIFER and XHMM, we used these two tools by adding 80 additional samples to the 20 used with EXCAVATOR and ExomeCNV. To this end we used: 20 CHS (Han Chinese South, China) HG00403, HG00404, HG00406, HG00407, HG00418, HG00419, HG00421, HG00422, HG00427, HG00428, HG00436, HG00437, HG00442, HG00443, HG00445, HG00446, HG00448, HG00449, HG00451, HG00452, 20 TSI, Toscani in Italia, (NA20766, NA20768, NA20769, NA20770, NA20771, NA20772, NA20773, NA20774, NA20775, NA20778, NA20783, NA20785, NA20786, NA20787, NA20790, NA20792, NA20795, NA20807, NA20808, NA20811), 12 YRI, Yoruba in Ibadan, (NA18504, NA18517, NA18519, NA18861, NA18871, NA18874, NA19093, NA19102, NA19137, NA19152, NA19197, NA19248) 16 CEU, Utah residents with Northern and Western European ancestry, (NA06984, NA10847, NA11843, NA11930, NA12045, NA12272, NA12273, NA12275, NA12340, NA12341, NA12342, NA12718, NA12748, NA12830, NA12842, NA12843) and 12 JPT, Japanese in Tokyo, (NA18986, NA18999, NA19065, NA19075, NA19076, NA19077, NA19079, NA19080, NA19082, NA19083, NA19085, NA19088). For all the 100 samples whole-exome capture was performed by using the Agilent SureSelect_All_Exon_V2 kit and sequencing was made by using the Illumina HiSeq2000 platform. All the reads of the 100 samples were aligned against the Human reference genome hg19 by means of the BWA aligner. The whole-exome data were obtained for all the samples in the form of .bam alignment files from `ftp://ftp-trace.ncbi.nih.gov/1000genomes/`.

1000 Genomes Project samples for EMRC data distribution analysis

To study the properties of EMRC data distributions we used 8 samples (see Supplemental Table 2 for more details): 7 samples of Yoruba ancestry (NA19138, NA19153, NA19206, NA19131, NA19223, NA19159, NA19152) and one sample of Caucasian ancestry (NA10847). The BAM files of the 8 samples were processed, sorted and filtered (discarding $MQ \leq 10$) with SAMtools and PCR duplicates were removed with Picard MarkDuplicates (`http://picard.sourceforge.net`). After duplicate removal we performed local realignment around indels using GATK (DePristo *et al*, 2007).

1000 Genomes Project samples for Population data analysis

Population data analysis was performed on the WES data of twenty healthy individuals (7 CEU, Utah residents with ancestry from Northern and Western European, 7 JPT, Japanese in Tokio and 6 YRI, Yoruba in Ibadan) using the WES data of an individual of Yoruba ancestry (NA19152) as control. The BAM file of the 21 samples were processed, sorted and filtered (discarding $MQ \leq 10$) with SAMtools and PCR duplicates were removed with Picard MarkDuplicates (`http://picard.sourceforge.net`). After duplicate removal we performed local realignment around indels using GATK (DePristo *et al*, 2007). After reads filtering, for each sample, we calculated EMRC for each exon and we corrected them for GC-content, exon length and mappability biases using the median-normalization approach described in method section. The normalized EMRC data of the 7 CEU, 7 JPT and 6 YRI individuals were compared to the normalized EMRC data of the NA19152 sample. The ratio between EMRC data from test and control was log2-transformed and then normalized by means of the lowess-scatter plot normalization procedure. Lowess-scatter plot normalization was used to remove coverage-dependent bias in the log2-ratio data and to normalize differences in coverage between test and control samples. The log2-EMRC ratio was analyzed by the HSLM (with $\omega = 0.1$, $\theta = 10^{-4}$ and $d_{Norm} = 10^5$) followed by the FastCall algorithm (setting cellularity parameter $c = 1$)).

Melanoma Dataset

We tested our computational pipeline on this dataset including six metastatic melanoma cell lines as tumor samples and six blood samples from healthy donors adopted as reference baseline. Metastatic melanoma cell lines were derived from metastasis tumor biopsies of as many stage IV melanoma patients and were kindly provided by Dr. Russo (Cancer Gene Therapy Unit, San Raffaele Scientific Institute,

Milan, Italy). Whole-exome-sequencing and Affymetrix SNP array experiments were conducted at Dr. De Bellis lab, at Institute for Biomedical Technologies of National Research Council (ITB-CNR), Milan, Italy.

Whole-exome capture and sequencing of tumor and healthy samples

Whole-exome capture was individually performed on tumor and reference samples using the Agilent SureSelect Human All Exon 50Mb kit (Agilent Technologies, Santa Clara, CA, USA), according to the SureSelectXT Target Enrichment System for Illumina Paired-End Sequencing Library protocol (version 1.1.1; Agilent Technologies). Briefly, 3 ug of genomic DNA per sample were sheared using a Covaris S2 AFA instrument (Covaris, Woburn, MA, USA) to a target peak size of 150-200 bp. After fragmentation size check by Agilent 2100 BioAnalyzer microcapillary electrophoresis on a DNA1000 Chip (Agilent Technologies), DNA fragments were end-repaired, adenylated at the 3-ends, ligated to paired-end specific adapters and then amplified by PCR (6 cycles) with Herculase II polymerase. Once purified by Agencourt AMPure XP beads (Beckman Coulter, Brea, CA, USA), pre-capture libraries were assessed for size and quantity using the Agilent 2100 BioAnalyzer instrument, on a DNA1000 Chip. Then, 500 ng of each library were individually incubated with the Agilent SureSelect biotinylated RNA capture baits at 65C for 24 hours, and then enriched for hybridized fragments by using streptavidin-coated magnetic Dynabeads on a Dynal separator (Invitrogen Life Technologies, Carlsbad, CA, USA). After washing and elution, post-capture libraries were amplified by PCR (12 cycles) and then assessed for size and quantity by Agilent 2100 BioAnalyzer instrument, on a DNA High Sensitivity Chip. Libraries were loaded, one sample per lane, onto the Illumina cBot Cluster Generation System (Illumina, San Diego, CA, USA) at a 8 pM final concentration, and then sequenced by Illumina Genome Analyzer IIx instrument, in a paired-end 76-cycle run. For raw data processing, we used the Illumina Sequencing Control Software (SCS) and the modules RTA (Real-Time Analysis) to convert raw image data into intensity scores, and OLB (Off-Line Basecaller) to convert .bcl files (intensity files) into qseq files, and then the Illumina CASAVA software to generate final fastq files (reads in fasta format). A summary of the total number of raw reads, the total number of reads aligned to the reference genome and the mean coverage generated for each sample is reported in Supplemental Table 3.

Melanoma sequencing data filtering and normalization

Raw reads in FASTQ format from each of the twelve samples were aligned to the human reference genome (hg19) with BWA using default parameters. Aligned reads were processed, sorted and filtered (discarding $MQ \leq 10$) with SAMtools and PCR duplicates were removed with Picard MarkDuplicates (`http://picard.sourceforge.net`). After duplicate removal we performed local realignment around indels using GATK (DePristo *et al*, 2007). We then calculated the EMRC for each targeted exon and we corrected for GC-content, exon size and mappability biases with the median-normalization approach described in method section. Since we do not have autologous normal samples to be used as matched control, we pooled exome-seq data from the six normal blood samples and used them as common reference baseline to study the genomic alterations in the six tumor samples. Data pooling was performed by summing the total number of reads on each exon across all the six samples. Then we calculated the EMRC for each exon of the seven samples (that is, six tumor samples and one control pool sample) and we corrected them for GC-content, exon size and mappability biases using the median-normalization approach described in Methods section. Finally we calculated the log_2-ratio between EMRC data from tumor sample and pool control and we normalized them by using the lowess-scatter plot normalization procedure. Lowess-scatter plot normalization was used to remove coverage-dependent bias in the log_2-ratio data and to normalize differences in coverage between test and control samples. The log2-EMRC ratio profiles were then analyzed by the HSLM (with $\omega = 0.1$, $\theta = 10^{-4}$ and $d_{Norm} = 10^5$) followed by the FastCallSeq algorithm (setting cellularity parameter $c = 0.7$)).

Affymetrix GeneChip 250K SNP Array preparation and analysis

Starting from 250 ng, DNA samples were prepared for whole-genome SNP profiling using the GeneChip Human Mapping 250K Nsp Assay kit (Affymetrix, Santa Clara, CA, USA), according to manufacturer's instructions, and hybridized onto GeneChip Human Mapping 250K Nsp SNP Arrays. Chips were washed

and stained on the Fluidics Station FS-450 (Affymetrix) and scanned by the GeneChip Scanner 3000 7G (Affymetrix). SNP signal intensities were acquired by GCOS software (Affymetrix) to generate raw intensity (.CEL) files, and SNP allelic calls were assigned using the BRLMM algorithm in GTYPE v4.1 software (Affymetrix), to generate CHP files. The Copy Number Analyzer for GeneChip software (CNAG, v3.0) was used to normalize the data in order to obtain the final log2ratio values for tumor samples (Yamamoto *et al*, 2007). Starting from .CEL files and brlmm.CHP files, CNAG was used to perform a non-self unpaired analysis by comparing each melanoma cell line to the common reference pool composed by the six normal blood samples and to convert probeset signal intensities into SNP CN values. Using default parameters, CNAG averaged these copy number values over windows of 10 contiguous SNPs after having first reduced noise by discarding data for the two SNPs with the highest and lowest copy number values in each window. Thus, we obtained a raw data matrix including the log2ratio values of single SNP for each melanoma sample. The normalized log_2-ratio values for each sample were analyzed by using the SLM segmentation algorithm (Magi *et al.*, 2010) (with $\omega = 0.1$, $\eta = 10^{-4}$) and each segment was classified by using the FastCall calling procedure (setting cellularity parameter $c = 0.7$)).

Intellectual disability data analysis

The analyses were conducted on two individuals with intellectual disabilities (ID1 and ID2) using a healthy individual of European descent as control. The ID1 sample was a 25 year old male, born at 42th week of gestation form healthy parents (second cousins). He showed a developmental delay with moderate mental retardation (QI about 50). During his first year of life, he presented recurrent episodes of hypoglicemia. He had progressive kyphoscoliosis and shows several dysmorphisms, including long face, long nose, short philtrum, high arched palate, large mouth and thick lips, with a prominent lower lip, and pes cavus. Conventional karyotype (500 bands) and fragile X test were normal, as all the metabolic examinations performed (including plasmatic and urinary amino acids, urinary organic acids, VLFA, oligosaccharides, isoelectrofocusing of sialotranferrin, mucopolysaccharide, thyroid function), and the following examinations: ophtalmologic evaluation, ABR, abdominal ultasound, muscle biopsy, brain MRI and cranial computerized tomography and EEG. The cardiological evaluation identified a sinusal bradychardia. His brother (ID2), a 20 year old male, born at term, showed developmental delay and mental retardation, gastro-esophageal reflux that required a surgical correction, and recurrent macroematuria/microematuria (not glomerular) in childhood. He shows several dysmorphisms including long face, high palate, large mouth and thick lips with a prominent lower lip and malocclusion. Echocardiogram revealed an ASD (atrial septal defects) type OS (ostium secundum). Both sibs were affected by recurrent diarrhoea.

Control Sample

As a control we used the WES data of a healthy individual of European descent sequenced by Clark *et al* (2011). As reported by the authors, exome enrichment was performed with the Illumina TruSeq Exome Enrichment Kit and the enriched libraries were subjected to Illumina paired-end sequencing with HiSeq 2000 platform in a 2×101 bp run. Exome sequencing data were downloaded at the Sequence Read Archive website under accession SRA040093. A summary of the total number of raw reads, the total number of reads aligned to the reference genome and the mean coverage generated for each sample is reported in Supplemental Table 4.

Whole-Exome Capture and sequencing of ID individuals

Using the Illumina TruSeq Exome Enrichment Kit and an HiSeq2000 system we captured and sequenced the coding sequences of the two siblings ID1 and ID2. We obtained more than 165M 100bp paired-end reads in each of the siblings. We aligned more than 98% reads on the hg19 reference genome with BWA (Li *et al.*, 2010), and more than the 97% were properly paired.

Intellectual disability WES data analysis

The reads of each of the three samples (ID1, ID2 and control) were aligned to the human reference genome (hg19) by using the BWA tool with default settings. Aligned reads were processed, sorted and filtered (discarding $MQ \leq 10$) with SAMtools and PCR duplicates were removed with Picard MarkDuplicates

(http://picard.sourceforge.net). After duplicate removal we performed local realignment around indels using GATK DePristo *et al* (2007). We then calculated the EMRC for each targeted exon and we corrected for GC-content, exon size and mappability biases with the median-normalization approach described in method section. Finally we calculated the log2-ratio between the ID samples and the healthy control. EMRC log2-ratio data were normalized by using the lowess-scatter plot normalization procedure and then analyzed by usingthe HSLM (with $\omega = 0.1$, $\theta = 10^{-4}$ and $d_{Norm} = 10^5$) followed by the FastCall algorithm (setting cellularity parameter $c = 1$).

Affymetrix GeneChip SNP6.0 Array preparation and analysis

Genomic DNA from peripheral blood was extracted using the QIAGEN blood Midi Kit according to manufactures instructions. For each sample, two separate restriction digestion reactions were carried out with NspI or StyI enzymes (New England Biolabs) using 250 ng genomic DNA each, in order to generate the targets according to the Genome-Wide Human SNP Array 6.0 protocol (Affymetrix). Targets were hybridized according to manufacturer's recommendations onto the SNP Array 6.0 chips, that contains 906,000 SNP probes and 946,000 probes for CNV detection. Raw intensity signals data in form of cel files were normalized by using the Partek Genomic Suite for Genome-Wide Human SNP Array 6.0 (Partek Inc., St Louis, MO, USA). The normalized $log_2 - ratio$ values for each sample were analyzed by using the SLM segmentation algorithm (Magi *et al.*, 2010) (with $\omega = 0.1$, $\eta = 10^{-4}$) and each segment was classified by using the FastCall calling procedure (setting cellularity parameter $c = 1$).

References

Clark MJ, Chen R, Lam HYK, Karczewski KJ, Chen R, Euskirchen G, Butte AJ and Snyder M. (2011) Performance comparison of exome DNA sequencing technologies. *Nature Biotechnology*, Sep 25;29(10):908-914.

Conrad,D.F., Pinto,D., Redon,R., Feuk,L., Gokcumen,O., Zhang,Y., Aerts,J., Andrews,T.D., Barnes,C., Campbell,P. *et al.* (2010) Origins and functional impact of copy number variation in the human genome. *Nature*, **464**, 704712.

Dempster, A. P., Laird, N. M. and Rubin, D. B. (1977) Maximum Likelihood from Incomplete Data via the EM Algorithm. *Journal of the Royal Statistical Society.* **39-1**, 1-38.

DePristo MA, Banks E, Poplin R, Garimella KV, Maguire JR, Hartl C, Philippakis AA, del Angel G, Rivas MA, Hanna M, McKenna A, Fennell TJ, Kernytsky AM, Sivachenko AY, Cibulskis K, Gabriel SB, Altshuler D, Daly MJ. (2011) A framework for variation discovery and genotyping using next-generation DNA sequencing data. *Nat Genet.* **43**:491-498.

1000 Genomes Project Consortium, Durbin,R.M., Abecasis,G.R., Altshuler,D.L., Auton,A., Brooks,L.D., Gibbs,R.A., Hurles,M.E., McVean,G.A. (2010) A map of human genome variation from population-scale sequencing. *Nature*, **467**, 1061-1073.

Fromer,M., Mora,J.L., Chambert,K., Banks,E., Bergen,S.E., Ruderfer,D.M., Handsaker,R.E., McCarroll,S.A., O'Donovan,M.C., Owen,M.J., Kirov,G., Sullivan,P.F., Hultman,C.M., Sklar,P., Purcell,S.M. (2012) Discovery and statistical genotyping of copy-number variation from whole-exome sequencing depth.*Am J Hum Genet.* **91**:597-607.

Harismendy,O., Ng,P.C., Strausberg,R.L., Wang,X., Stockwell,T.B., Beeson,K.Y., Schork,N.J., Murray,S.S., Topol,E.J., Levy,S., Frazer,K.A. (2009) Evaluation of next generation sequencing platforms for population targeted sequencing studies. *Genome Biol.*, **10**, R32.

Homer,N., Merriman,B., Nelson,S.F. (2009) BFAST: an alignment tool for large scale genome resequencing. *PLoS One*, **11**, e7767.

Krumm,N., Sudmant,P.H., Ko,A., O'Roak,B.J., Malig,M., Coe,B.P.; NHLBI Exome Sequencing Project, Quinlan,A.R., Nickerson,D.A., Eichler,E.E. (2012) Copy number variation detection and genotyping from exome sequence data. *Genome Res.* **22**:1525-1532.

Lai,W.R.R., Johnson,M.D.D., Kucherlapati,R. and Park,P.J.J. (2005) Comparative analysis of algorithms for identifying amplifications and deletions in array-CGH data. *Bioinformatics*, **21**, 3763-3770.

Langmead,B., Trapnell,C., Pop,M., Salzberg,S.L. (2009) Ultrafast and memory-efficient alignment of short DNA sequences to the human genome. *Genome Biol*, **10**, R25.

Li,H., Ruan,J., Durbin,R. (2008) Mapping short DNA sequencing reads and calling variants using mapping quality scores. *Genome Res*, **18**, 1851-1058.

Li,H., Handsaker,B., Wysoker,A., Fennell,T., Ruan,J., Homer,N., Marth,G., Abecasis,G., Durbin,R. and 1000 Genome Project Data Processing Subgroup (2009) The Sequence alignment/map (SAM) format and SAMtools. *Bioinformatics*, **25**, 2078-2079.

Li,H., Durbin,R. (2010) Fast and accurate long-read alignment with Burrows-Wheeler transform. *Bioinformatics*, **26**, 589-595.

Magi,A., Benelli,M., Marseglia,G., Nannetti,G., Scordo,M.R., Torricelli,F. (2010) A shifting level model algorithm that identifies aberrations in array-CGH data. *Biostatistics*, **11**, 265-280.

Magi,A., Benelli,M., Yoon,S., Roviello,F., Torricelli,F. (2011) Detecting common copy number variants in high-throughput sequencing data by using JointSLM algorithm. *Nucleic Acids Res.*, Feb 14. [Epub ahead of print].

Magi A, Tattini L, Pippucci T, Torricelli F, Benelli M. Read count approach for DNA copy number variants detection. Bioinformatics. 2012 Feb 15;28(4):470-8.

McCarroll,S., Kuruvilla,F., Korn,J., Cawley,S., Nemesh,J., Wysoker,A., Shapero,M., de Bakker,P., Maller,J., Kirby,A. *et al.* (2008) Integrated detection and population-genetic analysis of SNPs and copy number variation. *Nat Genet*, **40**, 1166-1174.

Miller,C.A., Hampton,O., Coarfa,C., Milosavljevic,A. (2011) ReadDepth: a parallel R package for detecting copy number alterations from short sequencing reads. *PLoS One*, **6**, e16327.

Ning,Z., Cox,A.J., Mullikin,J.C. (2001) SSAHA: a fast search method for large DNA databases. *Genome Res.*, **11**, 1725-1729.

Olshen, A.B., Venkatraman, E.S., Lucito, R. and Wigler, M. (2005) Circular binary segmentation for the analysis of array-based DNA copy number data. *Biostatistics*, **5**, 557-572.

Sathirapongsasuti, J. F., Lee, H., Horst, B. A. J., Brunner, G., Cochran, A. J., Binder, S., Quackenbush, J., et al. (2011). Exome sequencing-based copy-number variation and loss of heterozygosity detection: ExomeCNV. *Bioinformatics*, **27**, 2648 2654.

van de Wiel, M.A, Kim, K.I., Vosse, S.J., van Wieringen, W.N., Wilting, S.M., Ylstra, B. (2007) CGHcall: calling aberrations for array CGH tumor profiles. *Bioinformatics*,**23**, 892-894.

Yamamoto,G., Nannya,Y., Kato,M., Sanada,M., Levine,R.L., Kawamata,N., Hangaishi,A., Kurokawa,M., Chiba,S., Gilliland,D.G., Koeffler,H.P., Ogawa,S. (2007) Highly sensitive method for genomewide detection of allelic composition in nonpaired, primary tumor specimens by use of affymetrix single-nucleotide-polymorphism genotyping microarrays. *Am J Hum Genet.*, **81(1)**, 114-126.

Yoon, S., Xuan, Z., Makarov, V., Ye, K and Sebat, J. (2009) Sensitive and accurate detection of copy number variants using read depth of coverage. *Genome Res.*, **19**, 1586-1592.

Table 1: Lower and upper bounds for the five truncated gaussians.

Bound	Double Loss	Loss	Neutral	Gain	Multiple Gain
Lower	$-\infty$	$\mu_2 - 3 \cdot \sigma_2$	$-\epsilon_d$	$+\epsilon_u$	$\mu_4 + 3 \cdot \sigma_4$
Upper	$\mu_2 - 3 \cdot \sigma_2$	$-\epsilon_d$	$+\epsilon_u$	$\mu_4 + 3 \cdot \sigma_4$	$+\infty$

Table 2: Summary statistics for the WES data generated by the 1000 Genomes Project consortium.

Sample	Ethnic group	Coverage on-target
NA10847	CEU	65.51x
NA11840	CEU	65.97x
NA12249	CEU	61.78x
NA12717	CEU	62.87x
NA12751	CEU	62.84x
NA12760	CEU	68.39
NA12761	CEU	52.85x
NA19138	YRI	96.76x
NA19153	YRI	105.02x
NA19206	YRI	103.12x
NA19131	YRI	50.29x
NA19223	YRI	104.23x
NA19159	YRI	97.06x
NA19152	YRI	106.99x
NA18966	JPT	94.30x
NA18970	JPT	103.02x
NA18967	JPT	102.67x
NA18959	JPT	119.48x
NA18973	JPT	60.67x
NA18981	JPT	64.60x
NA18999	JPT	128.25x

CEU= European ancestry. YRI=Yoruba Nigerian ethnicity. JPT= Japanese in Tokio.

Table 3: Summary statistics for the WES data of the melanoma dataset.

Sample	Raw Reads	Mapped Reads	Unambiguous Mapped Reads	Coverage on-target
Me01	69.72	61.11 (87.66%)	48.57 (69.67%)	44.54x
Me02	91.07	80.61 (88.51%)	60.81 (66.78%)	54.37x
Me04	84.45	74.49 (88.20%)	62.24 (73.70%)	50.78x
Me05	50.67	44.05 (86.94%)	39.29 (77.54%)	32.71x
Me08	74.52	65.20 (87.50%)	54.82 (73.57%)	42.78x
Me12	83.75	73.41 (87.65%)	60.54 (72.28%)	46.38x
Sample01	81.87	74.30 (90.75%)	67.28 (82.18%)	50.19x
Sample06	62.75	55.29 (88.11%)	49.13 (78.29%)	39.25x
Sample18	68.38	62.42 (91.28%)	50.76 (74.23%)	40.29x
Sample26	52.75	47.22 (89.52%)	41.70 (79.95%)	33.23x
Sample31	65.58	59.76 (91.12%)	52.64 (80.26%)	42.06x
Sample37	80.60	72.84 (90.37%)	66.24 (82.18%)	47.52x

The number of reads is reported in millions. The Unambiguous Mapped Reads are calculated after duplicate removal with Picard MarkDuplicates.

Table 4: Summary statistics for the WES data of the ID dataset.

Sample	Raw Reads	Mapped Reads	Unambiguous Mapped Reads	Coverage on-target
ID1	116.87	97.39 (83.33%)	86.95 (74.40%)	63.20x
ID2	114.35	96.91 (84.74%)	85.75 (74.99%)	63.65x
HC	112.88	110.90 (90.35%)	88.76 (78.62%)	65.1x

The number of reads is reported in millions.The Unambiguous Mapped Reads are calculated after duplicate removal with Picard MarkDuplicates.

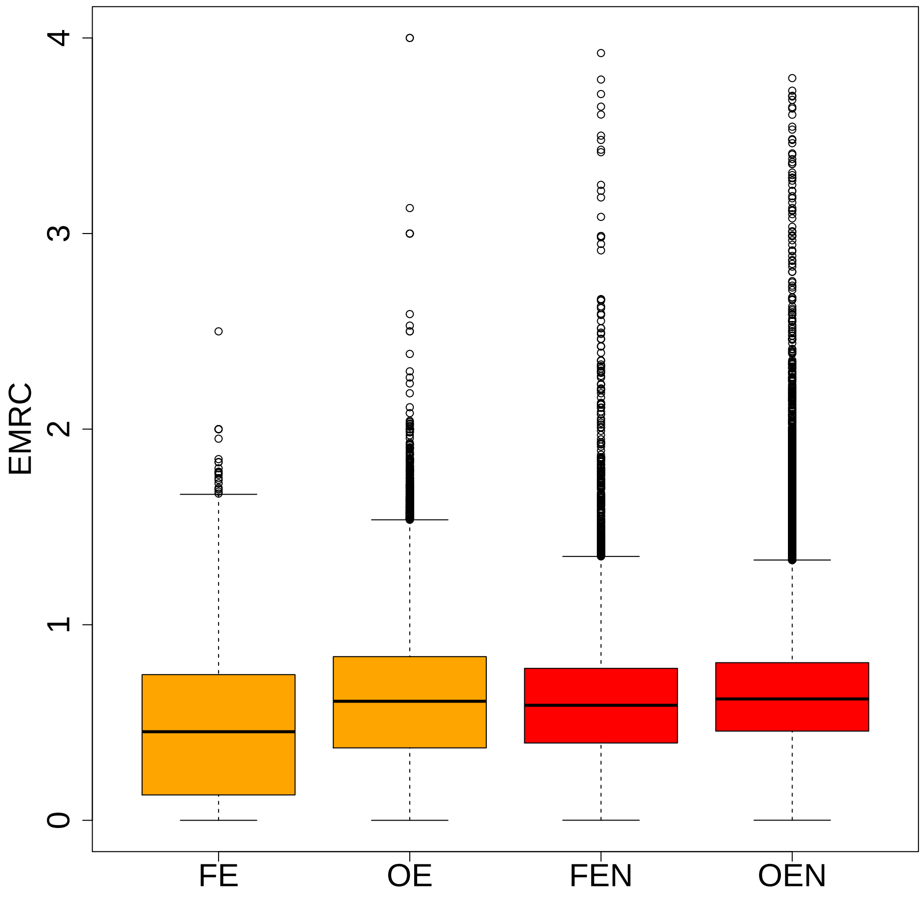

Supplemental Figure 1: Evaluation of the effect of normalization on the EMRC data of first and all the other exons. The four boxplots report the distribution of the raw EMRC data for the first exons (FE) and other exons (OE) and the distribution of the normalized EMRC data for the first exons (FEN) and other exons (OEN).

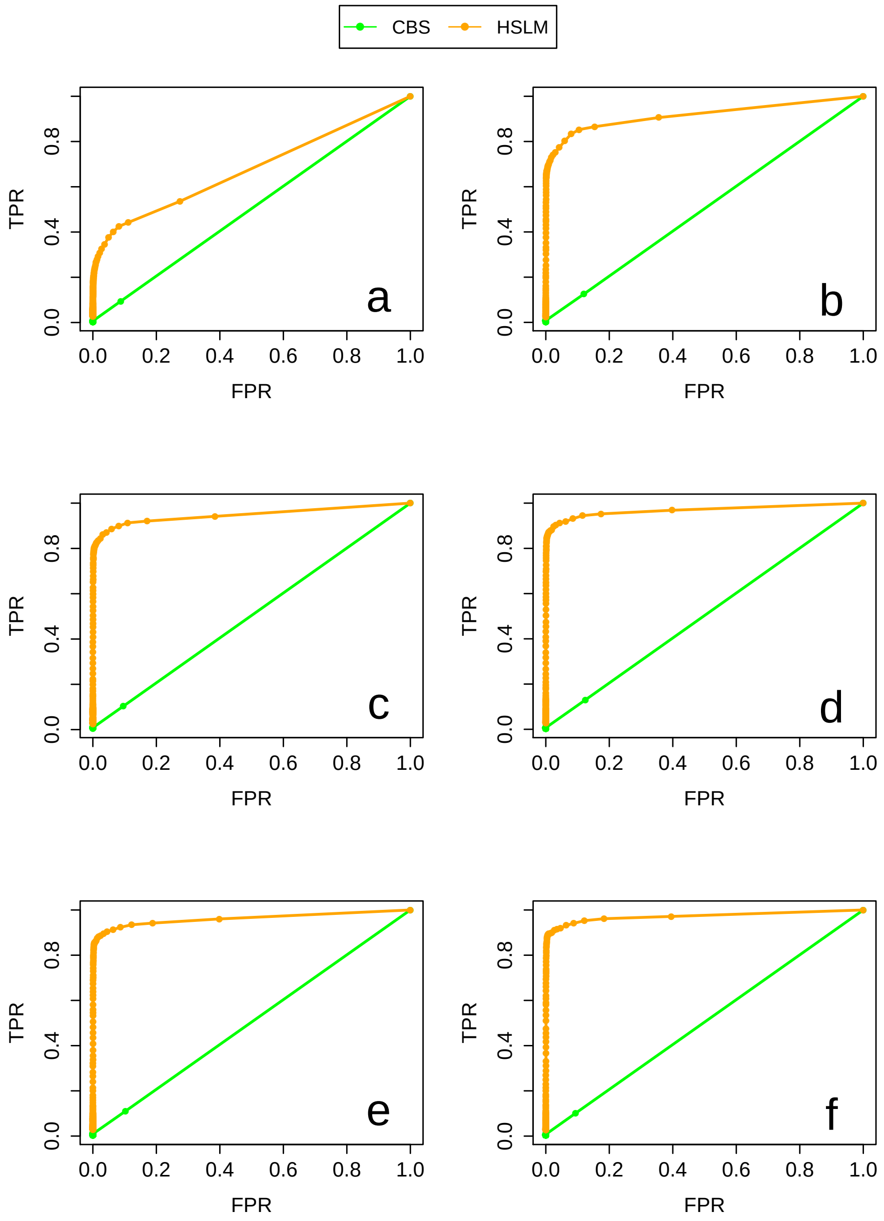

Supplemental Figure 2: Comparison between CBS and HSLM algorithms in the detection of 1-copy alterations made of $N = 2$ exons. The comparison analysis was performed for different values of the distance between adjacent genes ($D = 10Kb$, $D = 50Kb$, $D = 100Kb$, $D = 500Kb$, $D = 1Mb$, $D = 5Mb$). For the HSLM algorith the D_{Norm} was set equal to 10^3. The receiver operating characteristic (ROC) curve was calculated as in Lai *et al.* (2010) and for each of the two algorithms the ROC curve is averaged across 5000 simulations (1000 synthetic chromosome for each g=[1,2,3,4,5]). (a) $D = 10Kb$. (b) $D = 50Kb$. (c) $D = 100Kb$. (d) $D = 500Kb$. (e) $D = 1Mb$. (f) $D = 5Mb$.

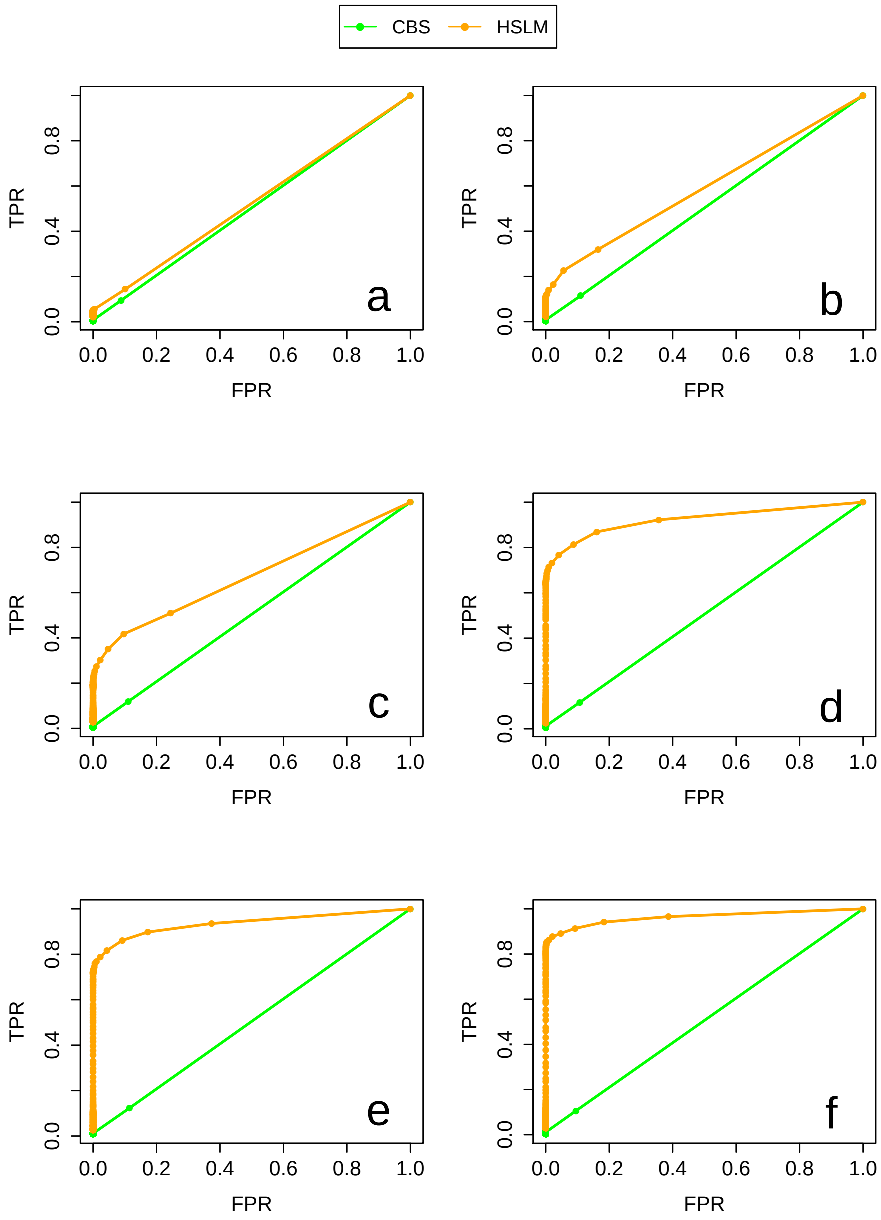

Supplemental Figure 3: Comparison between CBS and HSLM algorithms in the detection of 1-copy alterations made of $N = 2$ exons. The comparison analysis was performed for different values of the distance between adjacent genes ($D = 10Kb$, $D = 50Kb$, $D = 100Kb$, $D = 500Kb$, $D = 1Mb$, $D = 5Mb$). For the HSLM algorith the D_{Norm} was set equal to 10^4. The receiver operating characteristic (ROC) curve was calculated as in Lai *et al.* (2010) and for each of the two algorithms the ROC curve is averaged across 5000 simulations (1000 synthetic chromosome for each g=[1,2,3,4,5]). (a) $D = 10Kb$. (b) $D = 50Kb$. (c) $D = 100Kb$. (d) $D = 500Kb$. (e) $D = 1Mb$. (f) $D = 5Mb$.

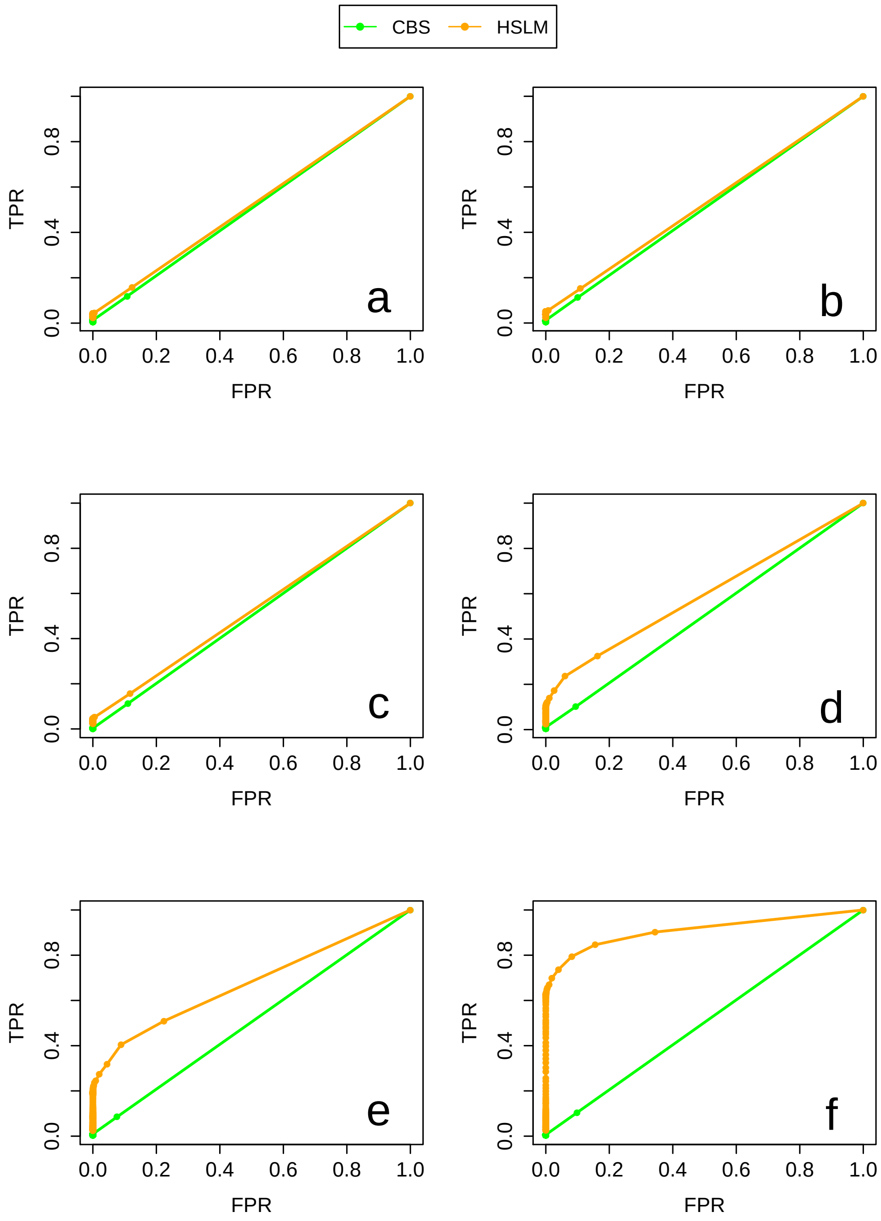

Supplemental Figure 4: Comparison between CBS and HSLM algorithms in the detection of 1-copy alterations made of $N = 2$ exons. The comparison analysis was performed for different values of the distance between adjacent genes ($D = 10Kb$, $D = 50Kb$, $D = 100Kb$, $D = 500Kb$, $D = 1Mb$, $D = 5Mb$). For the HSLM algorith the D_{Norm} was set equal to 10^5. The receiver operating characteristic (ROC) curve was calculated as in Lai *et al.* (2010) and for each of the two algorithms the ROC curve is averaged across 5000 simulations (1000 synthetic chromosome for each g=[1,2,3,4,5]). (a) $D = 10Kb$. (b) $D = 50Kb$. (c) $D = 100Kb$. (d) $D = 500Kb$. (e) $D = 1Mb$. (f) $D = 5Mb$.

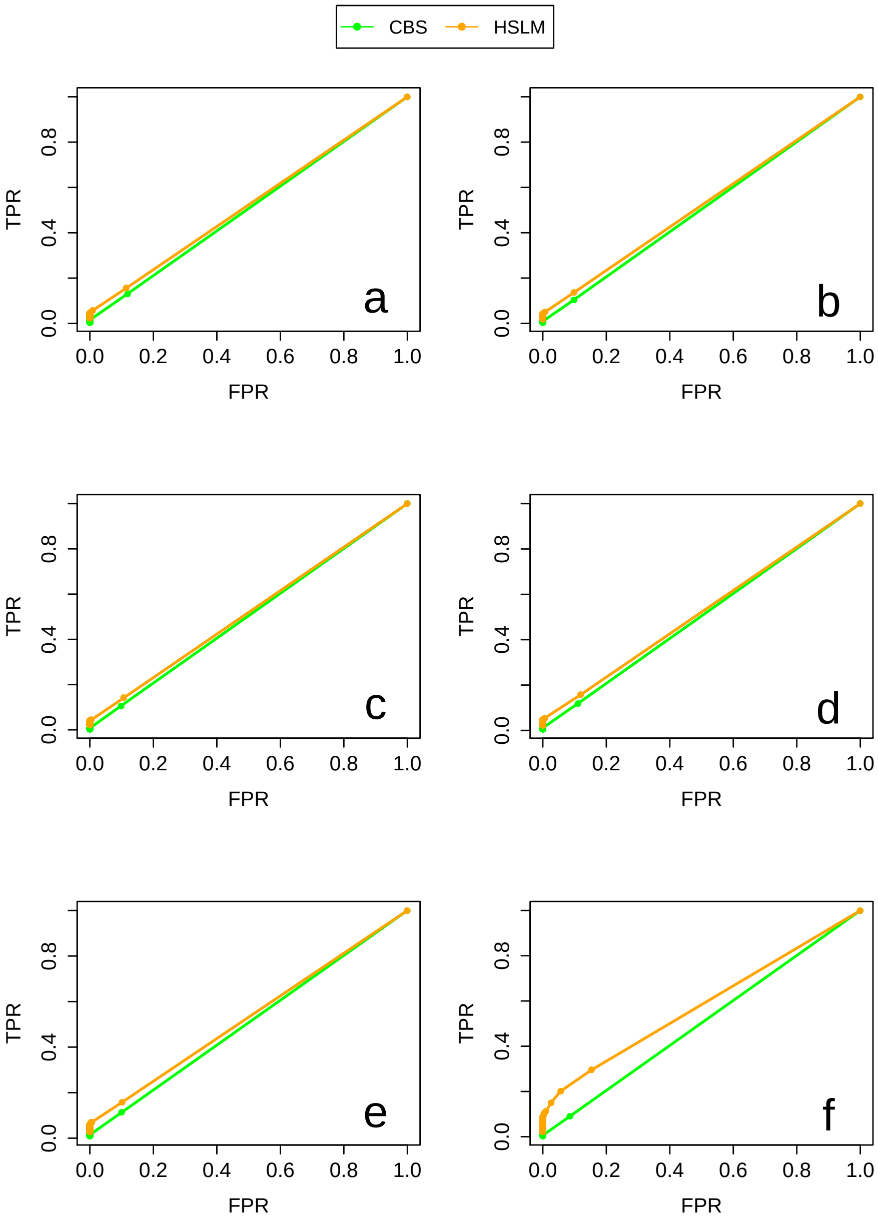

Supplemental Figure 5: Comparison between CBS and HSLM algorithms in the detection of 1-copy alterations made of $N = 2$ exons. The comparison analysis was performed for different values of the distance between adjacent genes ($D = 10Kb$, $D = 50Kb$, $D = 100Kb$, $D = 500Kb$, $D = 1Mb$, $D = 5Mb$). For the HSLM algorith the D_{Norm} was set equal to 10^6. The receiver operating characteristic (ROC) curve was calculated as in Lai *et al.* (2010) and for each of the two algorithms the ROC curve is averaged across 5000 simulations (1000 synthetic chromosome for each g=[1,2,3,4,5]). (a) $D = 10Kb$. (b) $D = 50Kb$. (c) $D = 100Kb$. (d) $D = 500Kb$. (e) $D = 1Mb$. (f) $D = 5Mb$.

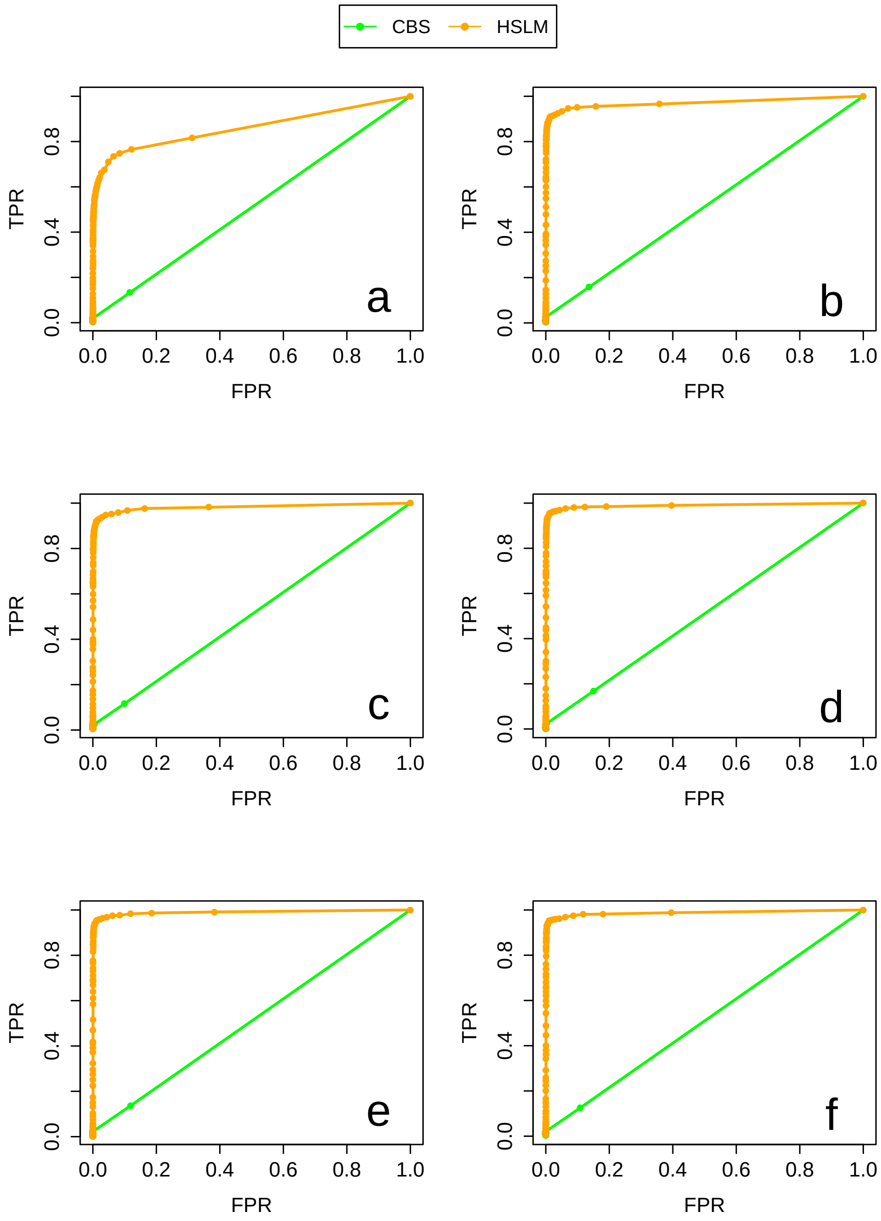

Supplemental Figure 6: Comparison between CBS and HSLM algorithms in the detection of 1-copy alterations made of $N = 3$ exons. The comparison analysis was performed for different values of the distance between adjacent genes ($D = 10Kb$, $D = 50Kb$, $D = 100Kb$, $D = 500Kb$, $D = 1Mb$, $D = 5Mb$). For the HSLM algorith the D_{Norm} was set equal to 10^3. The receiver operating characteristic (ROC) curve was calculated as in Lai *et al.* (2010) and for each of the two algorithms the ROC curve is averaged across 5000 simulations (1000 synthetic chromosome for each g=[1,2,3,4,5]). (a) $D = 10Kb$. (b) $D = 50Kb$. (c) $D = 100Kb$. (d) $D = 500Kb$. (e) $D = 1Mb$. (f) $D = 5Mb$.

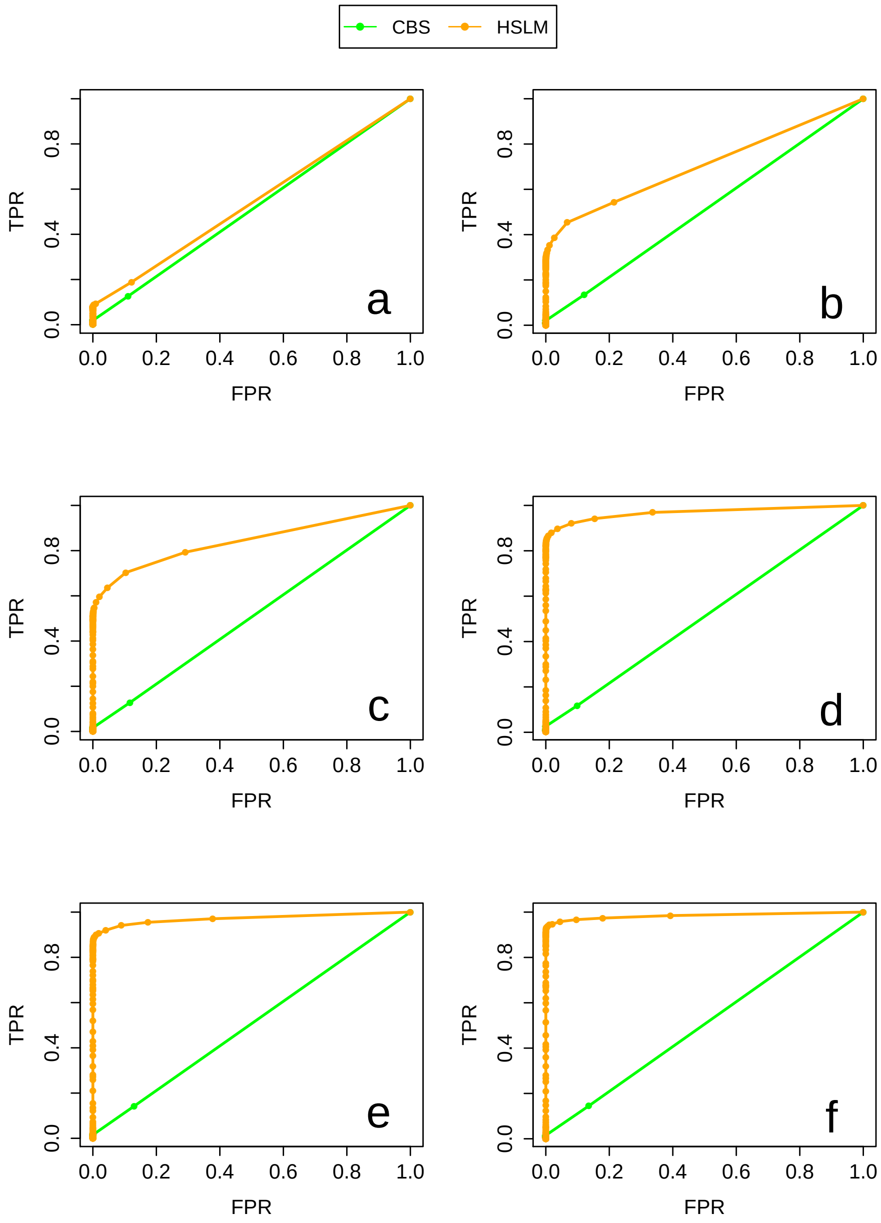

Supplemental Figure 7: Comparison between CBS and HSLM algorithms in the detection of 1-copy alterations made of $N = 3$ exons. The comparison analysis was performed for different values of the distance between adjacent genes ($D = 10Kb$, $D = 50Kb$, $D = 100Kb$, $D = 500Kb$, $D = 1Mb$, $D = 5Mb$). For the HSLM algorith the D_{Norm} was set equal to 10^4. The receiver operating characteristic (ROC) curve was calculated as in Lai *et al.* (2010) and for each of the two algorithms the ROC curve is averaged across 5000 simulations (1000 synthetic chromosome for each g=[1,2,3,4,5]). (a) $D = 10Kb$. (b) $D = 50Kb$. (c) $D = 100Kb$. (d) $D = 500Kb$. (e) $D = 1Mb$. (f) $D = 5Mb$.

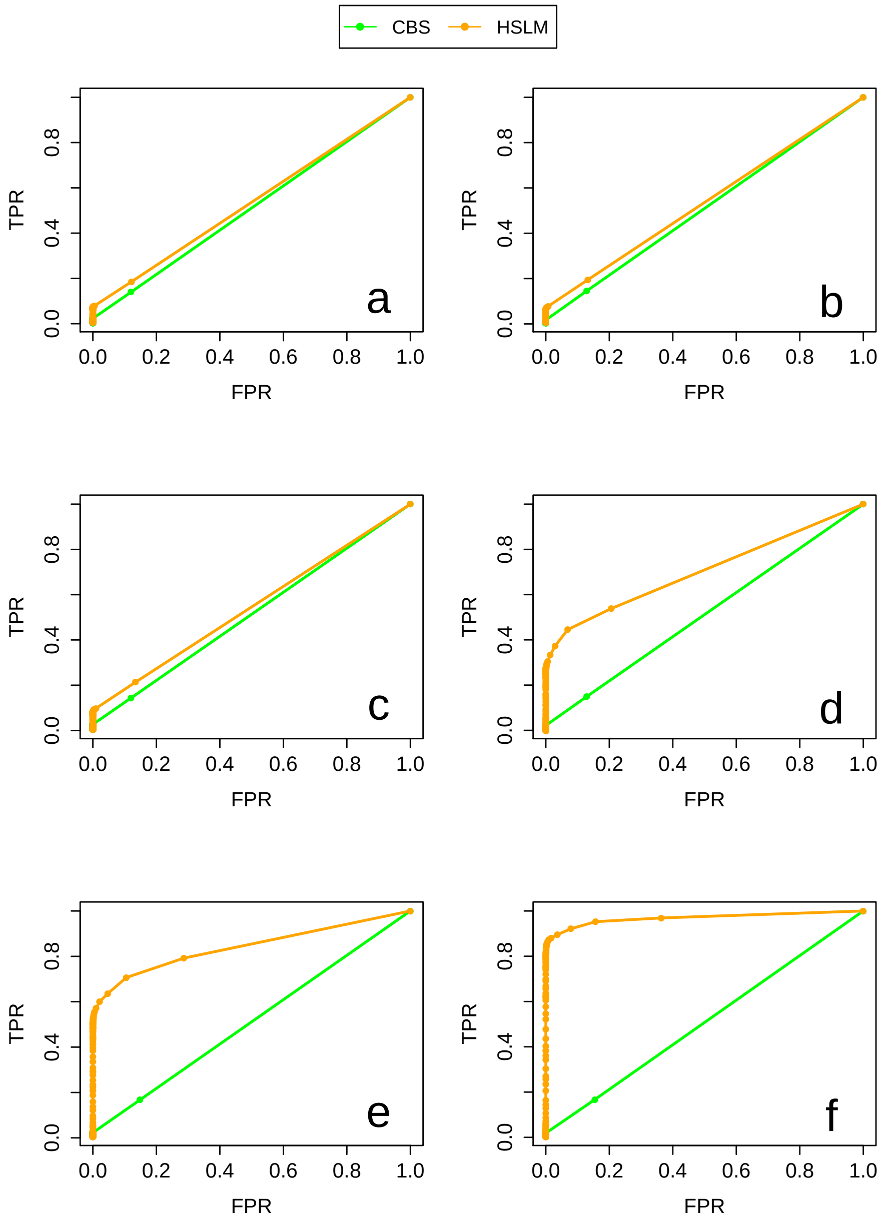

Supplemental Figure 8: Comparison between CBS and HSLM algorithms in the detection of 1-copy alterations made of $N = 3$ exons. The comparison analysis was performed for different values of the distance between adjacent genes ($D = 10Kb$, $D = 50Kb$, $D = 100Kb$, $D = 500Kb$, $D = 1Mb$, $D = 5Mb$). For the HSLM algorith the D_{Norm} was set equal to 10^5. The receiver operating characteristic (ROC) curve was calculated as in Lai *et al.* (2010) and for each of the two algorithms the ROC curve is averaged across 5000 simulations (1000 synthetic chromosome for each g=[1,2,3,4,5]). (a) $D = 10Kb$. (b) $D = 50Kb$. (c) $D = 100Kb$. (d) $D = 500Kb$. (e) $D = 1Mb$. (f) $D = 5Mb$.

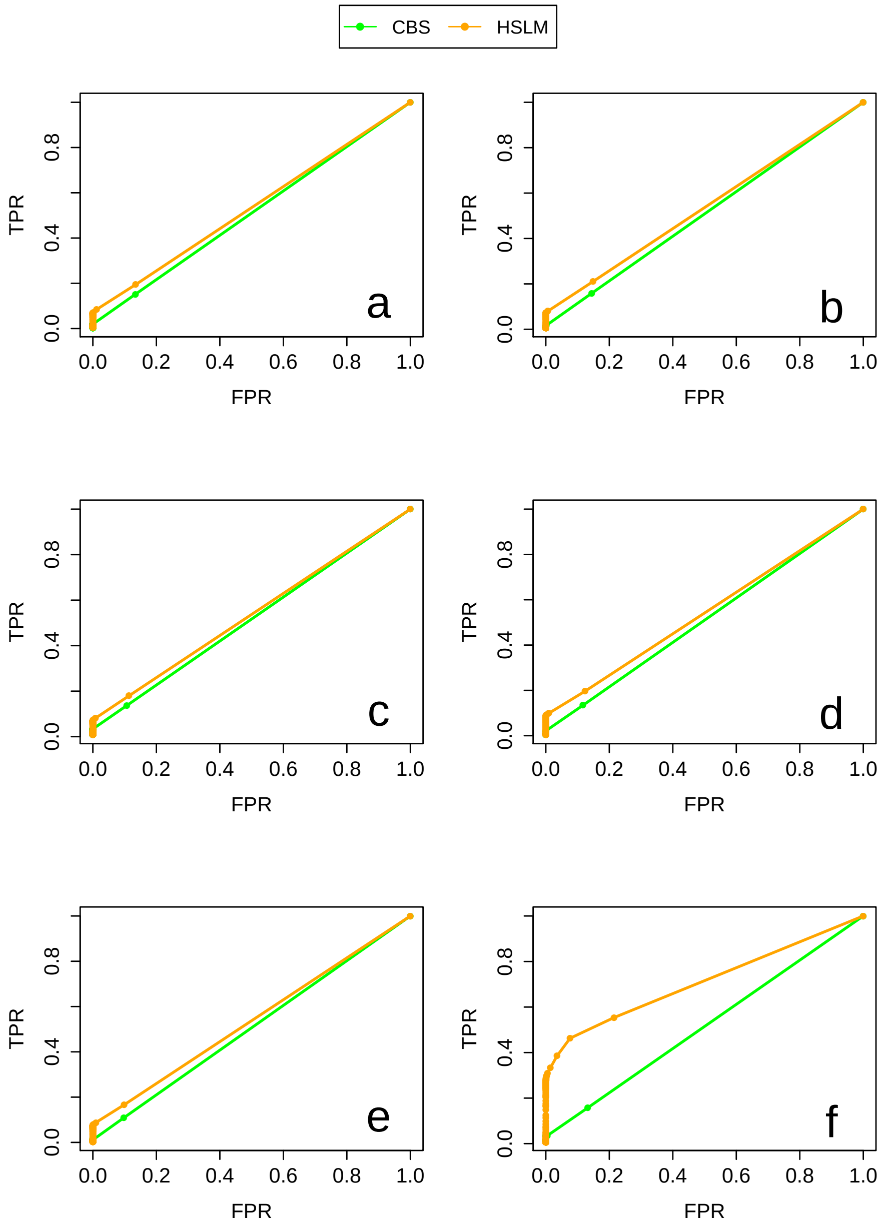

Supplemental Figure 9: Comparison between CBS and HSLM algorithms in the detection of 1-copy alterations made of $N = 3$ exons. The comparison analysis was performed for different values of the distance between adjacent genes ($D = 10Kb$, $D = 50Kb$, $D = 100Kb$, $D = 500Kb$, $D = 1Mb$, $D = 5Mb$). For the HSLM algorith the D_{Norm} was set equal to 10^6. The receiver operating characteristic (ROC) curve was calculated as in Lai *et al.* (2010) and for each of the two algorithms the ROC curve is averaged across 5000 simulations (1000 synthetic chromosome for each g=[1,2,3,4,5]). (a) $D = 10Kb$. (b) $D = 50Kb$. (c) $D = 100Kb$. (d) $D = 500Kb$. (e) $D = 1Mb$. (f) $D = 5Mb$.

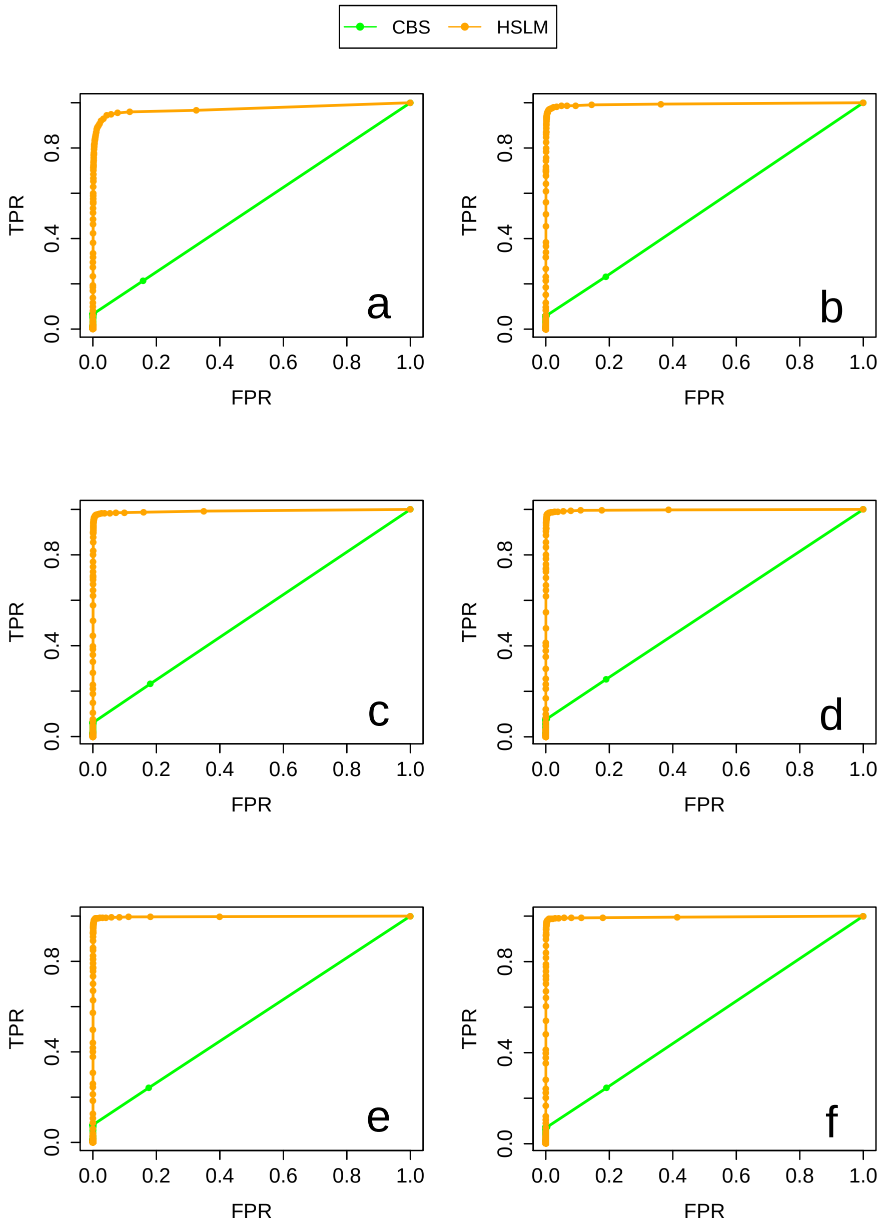

Supplemental Figure 10: Comparison between CBS and HSLM algorithms in the detection of 1-copy alterations made of $N = 5$ exons. The comparison analysis was performed for different values of the distance between adjacent genes ($D = 10Kb$, $D = 50Kb$, $D = 100Kb$, $D = 500Kb$, $D = 1Mb$, $D = 5Mb$). For the HSLM algorith the D_{Norm} was set equal to 10^3. The receiver operating characteristic (ROC) curve was calculated as in Lai *et al.* (2010) and for each of the two algorithms the ROC curve is averaged across 5000 simulations (1000 synthetic chromosome for each g=[1,2,3,4,5]). (a) $D = 10Kb$. (b) $D = 50Kb$. (c) $D = 100Kb$. (d) $D = 500Kb$. (e) $D = 1Mb$. (f) $D = 5Mb$.

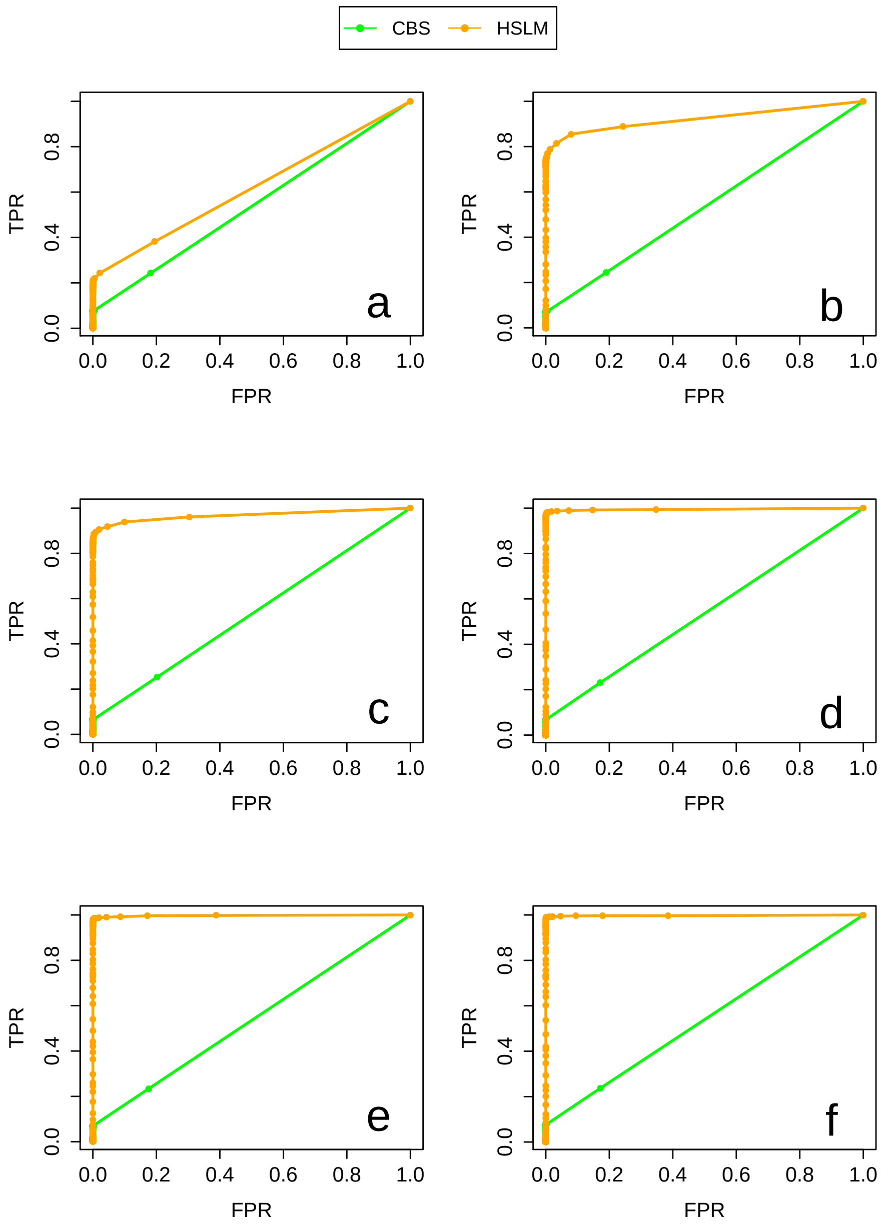

Supplemental Figure 11: Comparison between CBS and HSLM algorithms in the detection of 1-copy alterations made of $N = 5$ exons. The comparison analysis was performed for different values of the distance between adjacent genes ($D = 10Kb$, $D = 50Kb$, $D = 100Kb$, $D = 500Kb$, $D = 1Mb$, $D = 5Mb$). For the HSLM algorith the D_{Norm} was set equal to 10^4. The receiver operating characteristic (ROC) curve was calculated as in Lai *et al.* (2010) and for each of the two algorithms the ROC curve is averaged across 5000 simulations (1000 synthetic chromosome for each g=[1,2,3,4,5]). (a) $D = 10Kb$. (b) $D = 50Kb$. (c) $D = 100Kb$. (d) $D = 500Kb$. (e) $D = 1Mb$. (f) $D = 5Mb$.

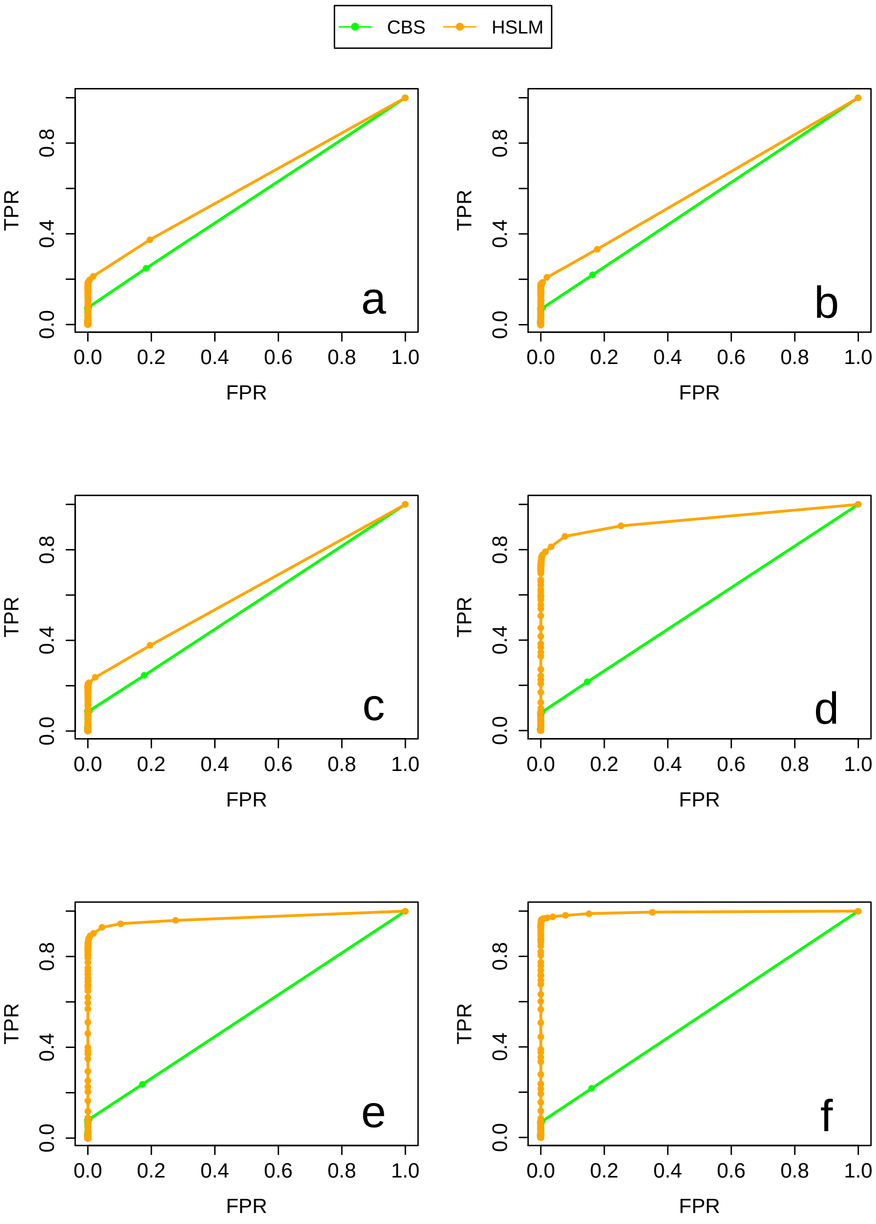

Supplemental Figure 12: Comparison between CBS and HSLM algorithms in the detection of 1-copy alterations made of $N = 5$ exons. The comparison analysis was performed for different values of the distance between adjacent genes ($D = 10Kb$, $D = 50Kb$, $D = 100Kb$, $D = 500Kb$, $D = 1Mb$, $D = 5Mb$). For the HSLM algorith the D_{Norm} was set equal to 10^5. The receiver operating characteristic (ROC) curve was calculated as in Lai *et al.* (2010) and for each of the two algorithms the ROC curve is averaged across 5000 simulations (1000 synthetic chromosome for each g=[1,2,3,4,5]). (a) $D = 10Kb$. (b) $D = 50Kb$. (c) $D = 100Kb$. (d) $D = 500Kb$. (e) $D = 1Mb$. (f) $D = 5Mb$.

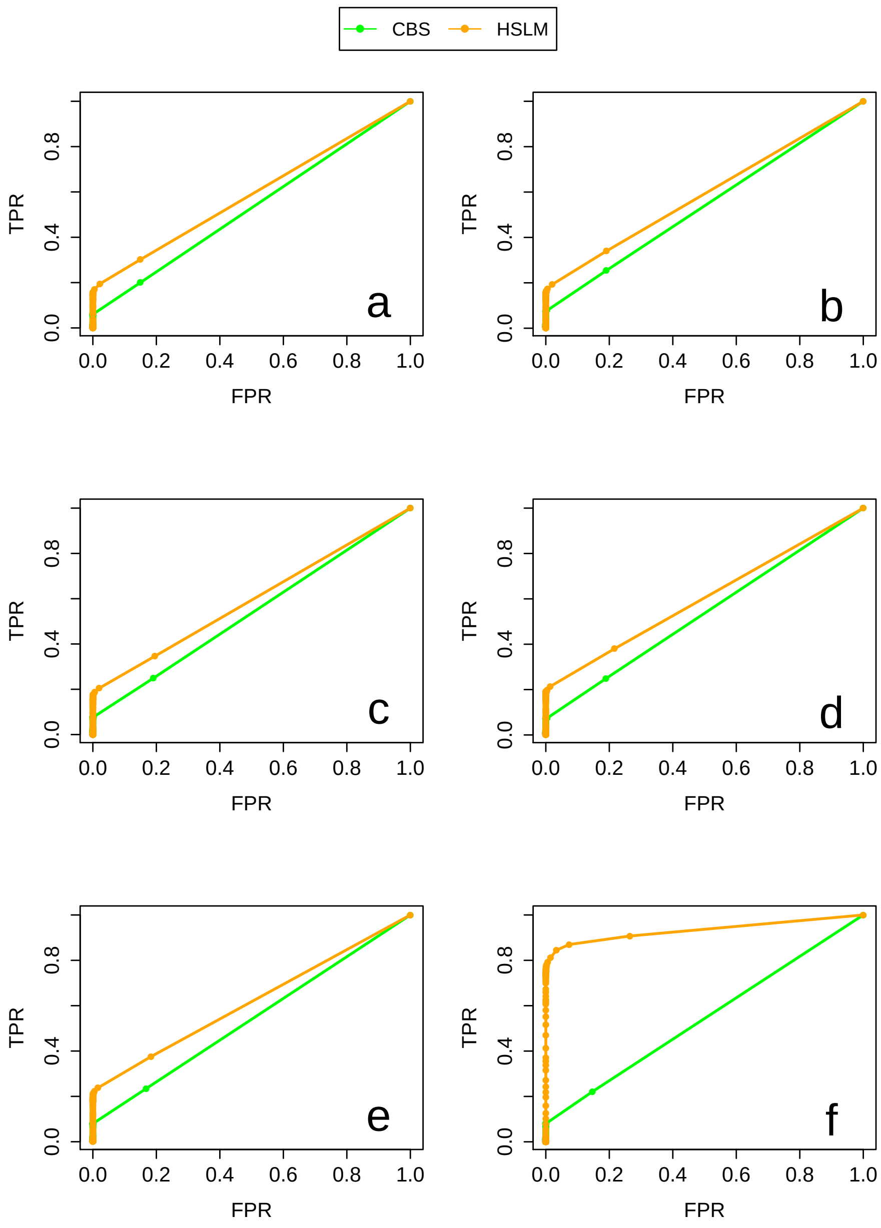

Supplemental Figure 13: Comparison between CBS and HSLM algorithms in the detection of 1-copy alterations made of $N = 5$ exons. The comparison analysis was performed for different values of the distance between adjacent genes ($D = 10Kb$, $D = 50Kb$, $D = 100Kb$, $D = 500Kb$, $D = 1Mb$, $D = 5Mb$). For the HSLM algorith the D_{Norm} was set equal to 10^6. The receiver operating characteristic (ROC) curve was calculated as in Lai *et al.* (2010) and for each of the two algorithms the ROC curve is averaged across 5000 simulations (1000 synthetic chromosome for each g=[1,2,3,4,5]). (a) $D = 10Kb$. (b) $D = 50Kb$. (c) $D = 100Kb$. (d) $D = 500Kb$. (e) $D = 1Mb$. (f) $D = 5Mb$.

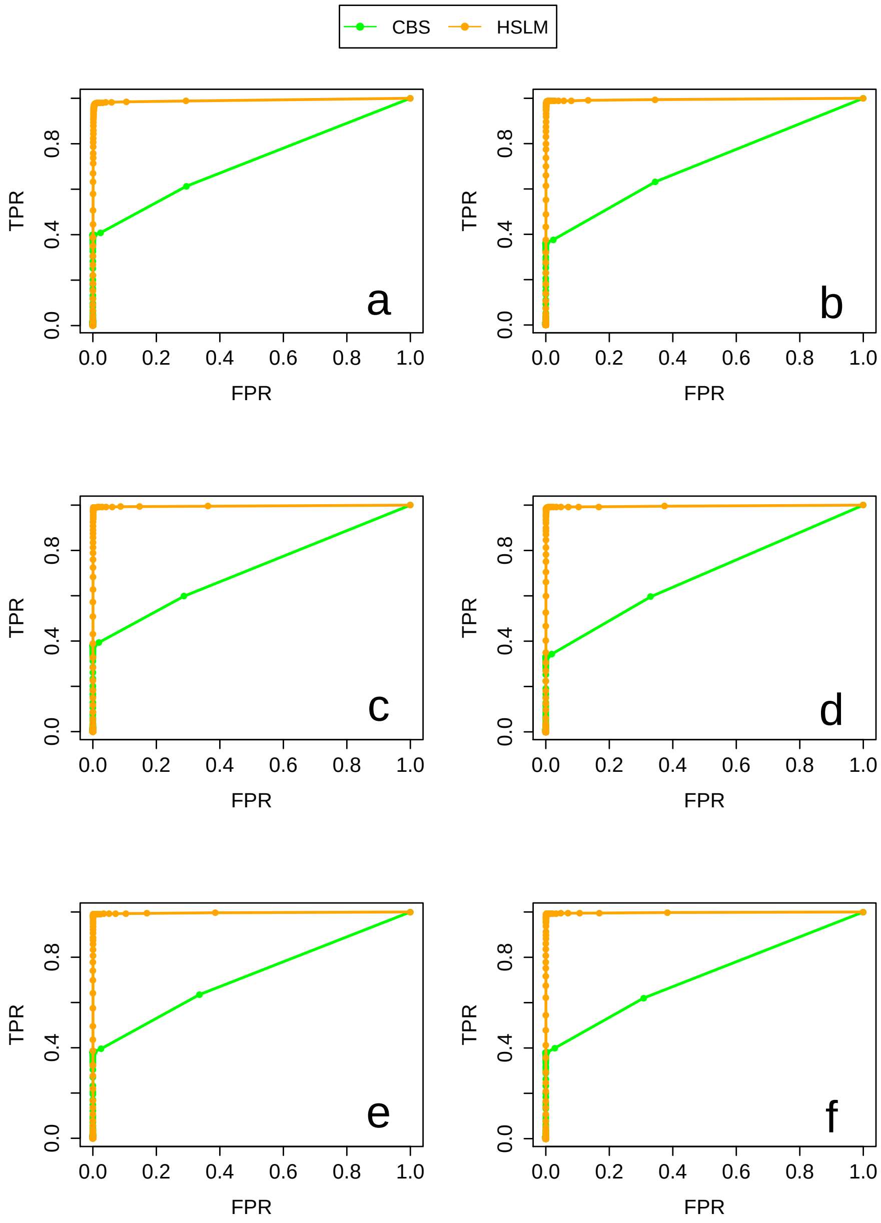

Supplemental Figure 14: Comparison between CBS and HSLM algorithms in the detection of 1-copy alterations made of $N = 10$ exons. The comparison analysis was performed for different values of the distance between adjacent genes ($D = 10Kb$, $D = 50Kb$, $D = 100Kb$, $D = 500Kb$, $D = 1Mb$, $D = 5Mb$). For the HSLM algorith the D_{Norm} was set equal to 10^3. The receiver operating characteristic (ROC) curve was calculated as in Lai *et al.* (2010) and for each of the two algorithms the ROC curve is averaged across 5000 simulations (1000 synthetic chromosome for each g=[1,2,3,4,5]). (a) $D = 10Kb$. (b) $D = 50Kb$. (c) $D = 100Kb$. (d) $D = 500Kb$. (e) $D = 1Mb$. (f) $D = 5Mb$.

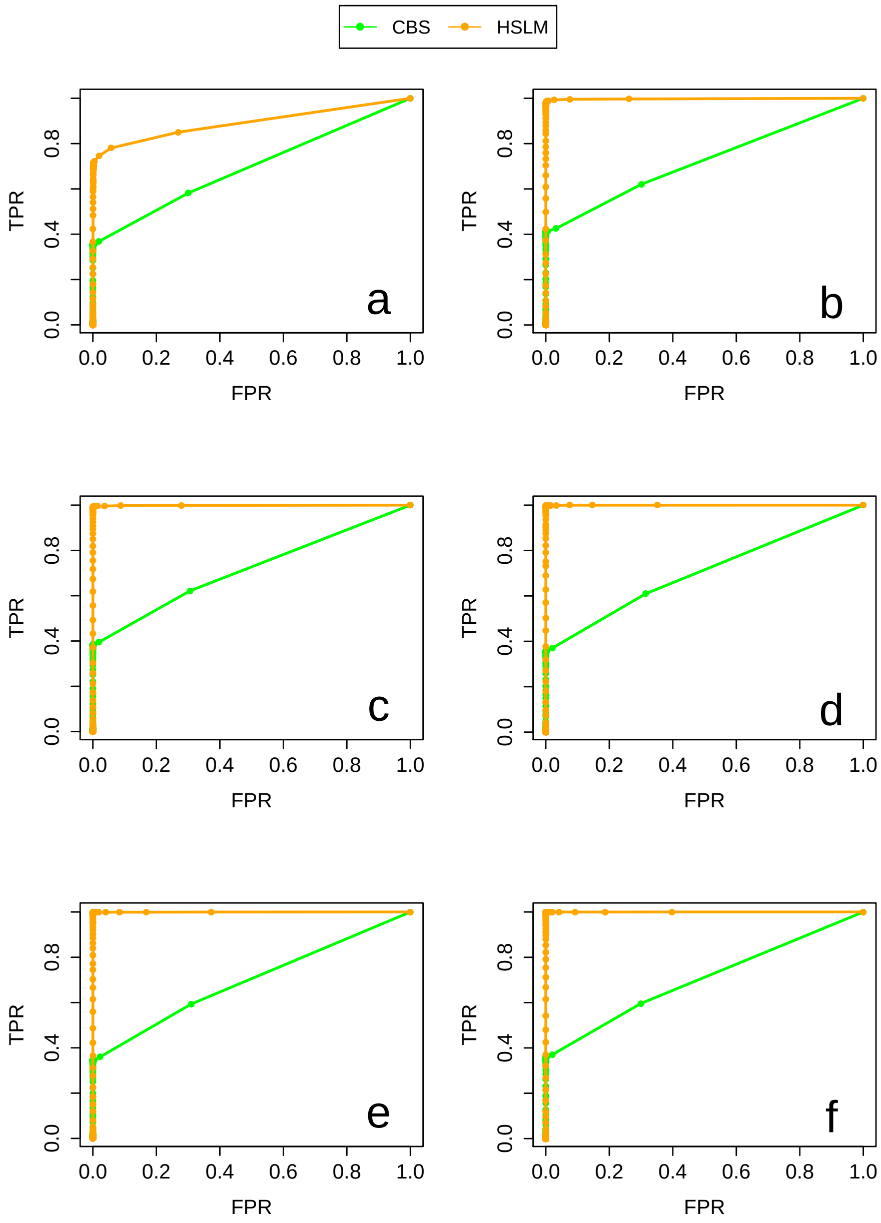

Supplemental Figure 15: Comparison between CBS and HSLM algorithms in the detection of 1-copy alterations made of $N = 10$ exons. The comparison analysis was performed for different values of the distance between adjacent genes ($D = 10Kb$, $D = 50Kb$, $D = 100Kb$, $D = 500Kb$, $D = 1Mb$, $D = 5Mb$). For the HSLM algorith the D_{Norm} was set equal to 10^4. The receiver operating characteristic (ROC) curve was calculated as in Lai *et al.* (2010) and for each of the two algorithms the ROC curve is averaged across 5000 simulations (1000 synthetic chromosome for each g=[1,2,3,4,5]). (a) $D = 10Kb$. (b) $D = 50Kb$. (c) $D = 100Kb$. (d) $D = 500Kb$. (e) $D = 1Mb$. (f) $D = 5Mb$.

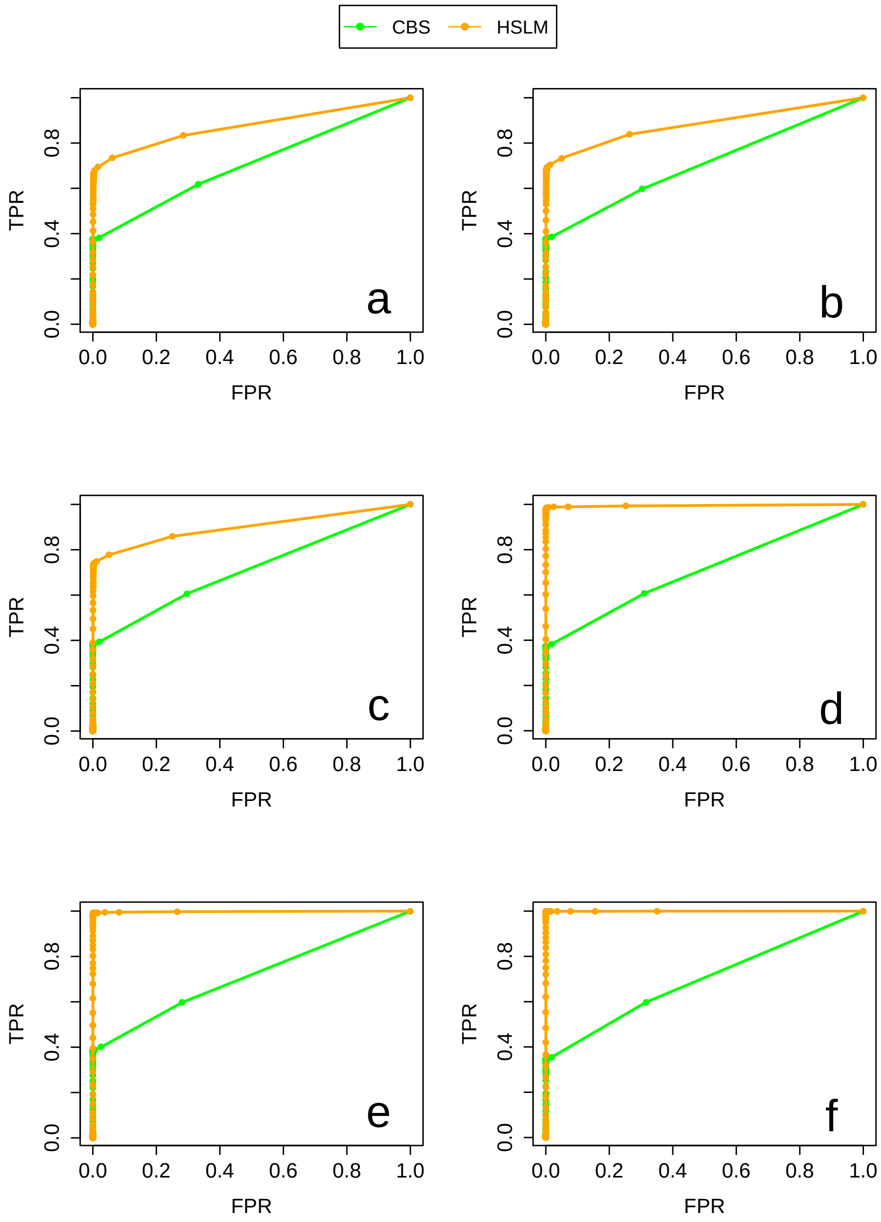

Supplemental Figure 16: Comparison between CBS and HSLM algorithms in the detection of 1-copy alterations made of $N = 10$ exons. The comparison analysis was performed for different values of the distance between adjacent genes ($D = 10Kb$, $D = 50Kb$, $D = 100Kb$, $D = 500Kb$, $D = 1Mb$, $D = 5Mb$). For the HSLM algorith the D_{Norm} was set equal to 10^5. The receiver operating characteristic (ROC) curve was calculated as in Lai *et al.* (2010) and for each of the two algorithms the ROC curve is averaged across 5000 simulations (1000 synthetic chromosome for each g=[1,2,3,4,5]). (a) $D = 10Kb$. (b) $D = 50Kb$. (c) $D = 100Kb$. (d) $D = 500Kb$. (e) $D = 1Mb$. (f) $D = 5Mb$.

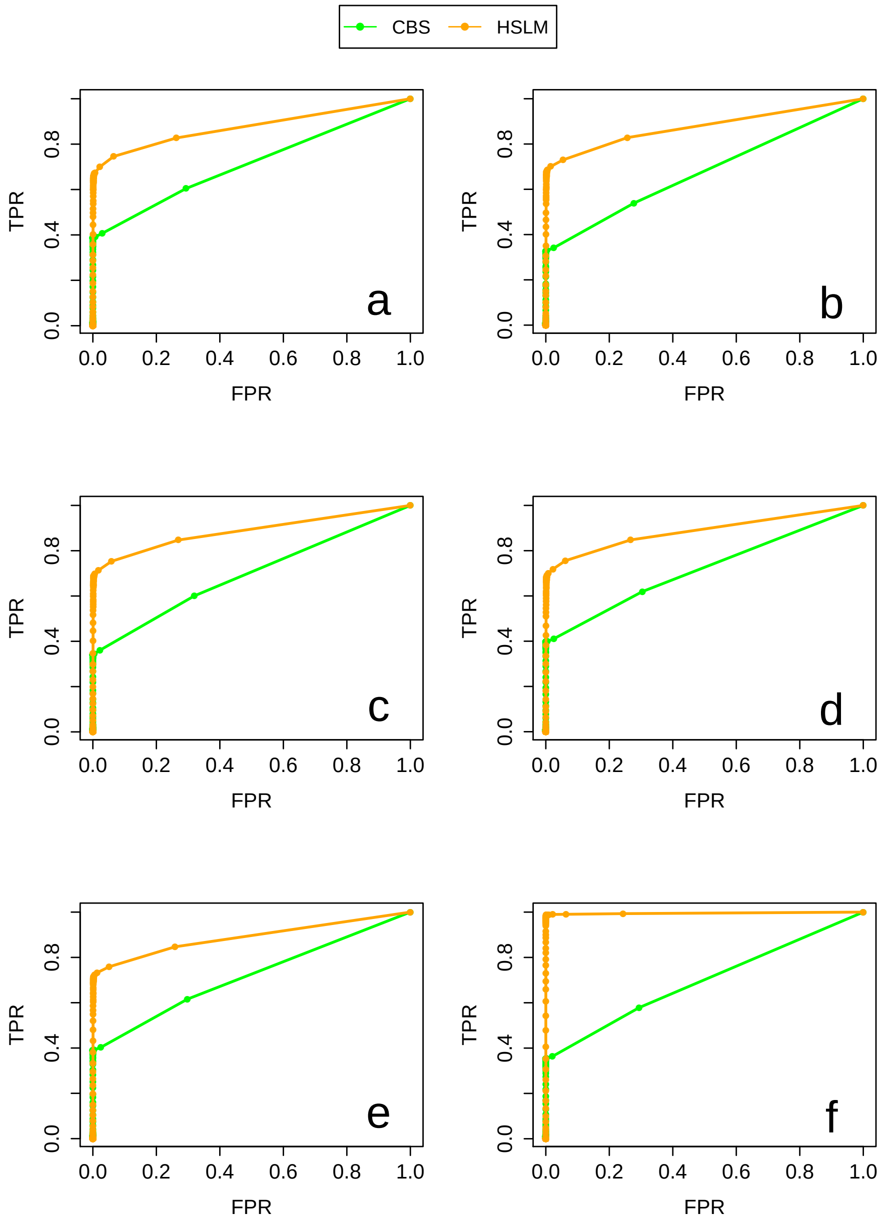

Supplemental Figure 17: Comparison between CBS and HSLM algorithms in the detection of 1-copy alterations made of $N = 10$ exons. The comparison analysis was performed for different values of the distance between adjacent genes ($D = 10Kb$, $D = 50Kb$, $D = 100Kb$, $D = 500Kb$, $D = 1Mb$, $D = 5Mb$). For the HSLM algorith the D_{Norm} was set equal to 10^6. The receiver operating characteristic (ROC) curve was calculated as in Lai *et al.* (2010) and for each of the two algorithms the ROC curve is averaged across 5000 simulations (1000 synthetic chromosome for each g=[1,2,3,4,5]). (a) $D = 10Kb$. (b) $D = 50Kb$. (c) $D = 100Kb$. (d) $D = 500Kb$. (e) $D = 1Mb$. (f) $D = 5Mb$.

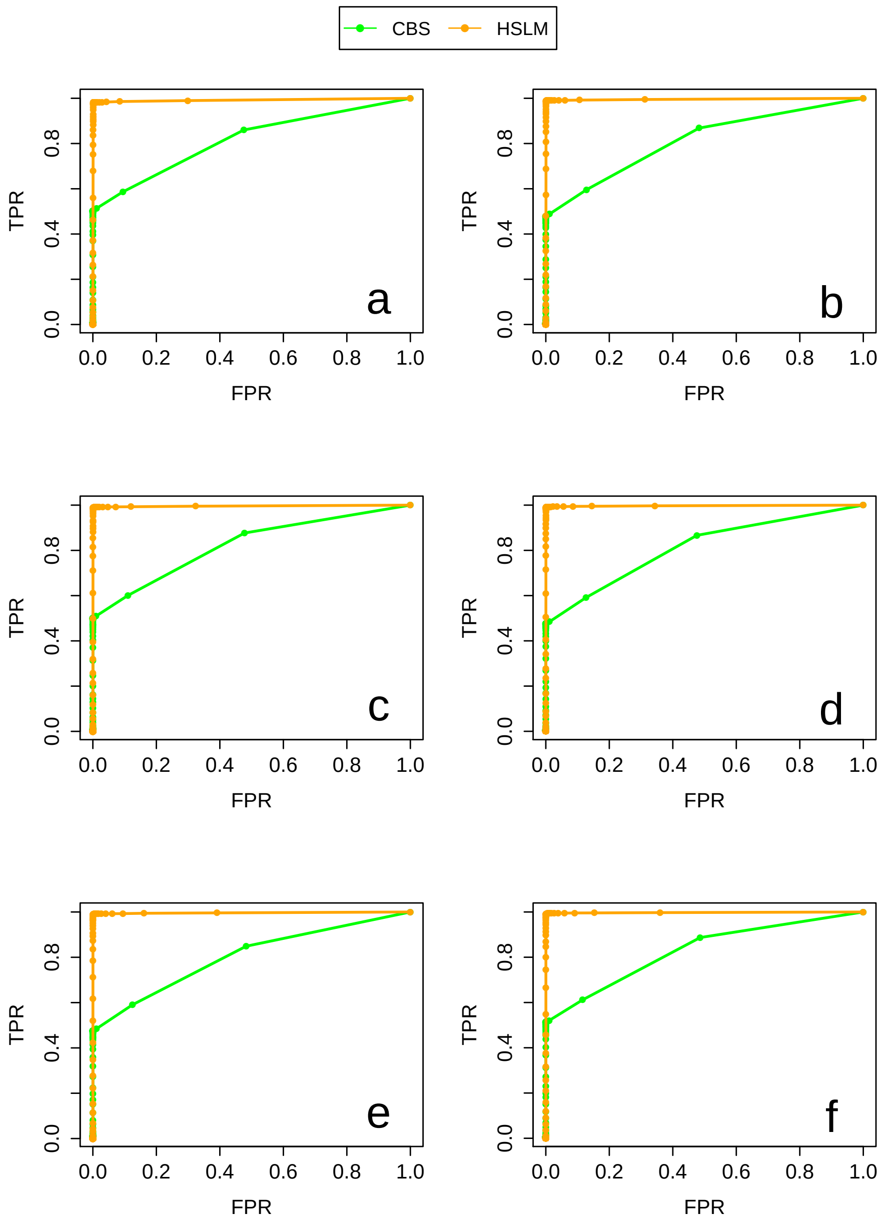

Supplemental Figure 18: Comparison between CBS and HSLM algorithms in the detection of 1-copy alterations made of $N = 20$ exons. The comparison analysis was performed for different values of the distance between adjacent genes ($D = 10Kb$, $D = 50Kb$, $D = 100Kb$, $D = 500Kb$, $D = 1Mb$, $D = 5Mb$). For the HSLM algorith the D_{Norm} was set equal to 10^3. The receiver operating characteristic (ROC) curve was calculated as in Lai *et al.* (2010) and for each of the two algorithms the ROC curve is averaged across 5000 simulations (1000 synthetic chromosome for each g=[1,2,3,4,5]). (a) $D = 10Kb$. (b) $D = 50Kb$. (c) $D = 100Kb$. (d) $D = 500Kb$. (e) $D = 1Mb$. (f) $D = 5Mb$.

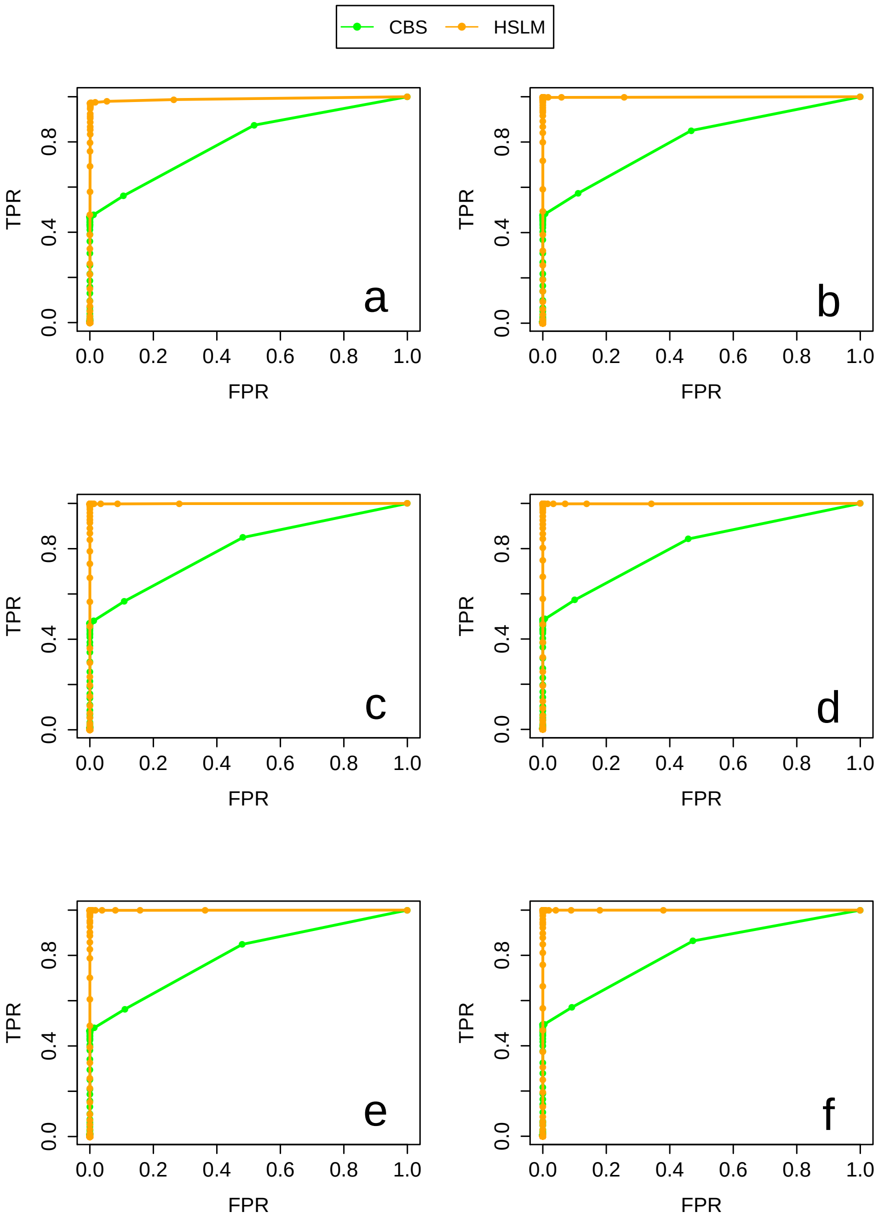

Supplemental Figure 19: Comparison between CBS and HSLM algorithms in the detection of 1-copy alterations made of $N = 20$ exons. The comparison analysis was performed for different values of the distance between adjacent genes ($D = 10Kb$, $D = 50Kb$, $D = 100Kb$, $D = 500Kb$, $D = 1Mb$, $D = 5Mb$). For the HSLM algorith the D_{Norm} was set equal to 10^4. The receiver operating characteristic (ROC) curve was calculated as in Lai *et al.* (2010) and for each of the two algorithms the ROC curve is averaged across 5000 simulations (1000 synthetic chromosome for each g=[1,2,3,4,5]). (a) $D = 10Kb$. (b) $D = 50Kb$. (c) $D = 100Kb$. (d) $D = 500Kb$. (e) $D = 1Mb$. (f) $D = 5Mb$.

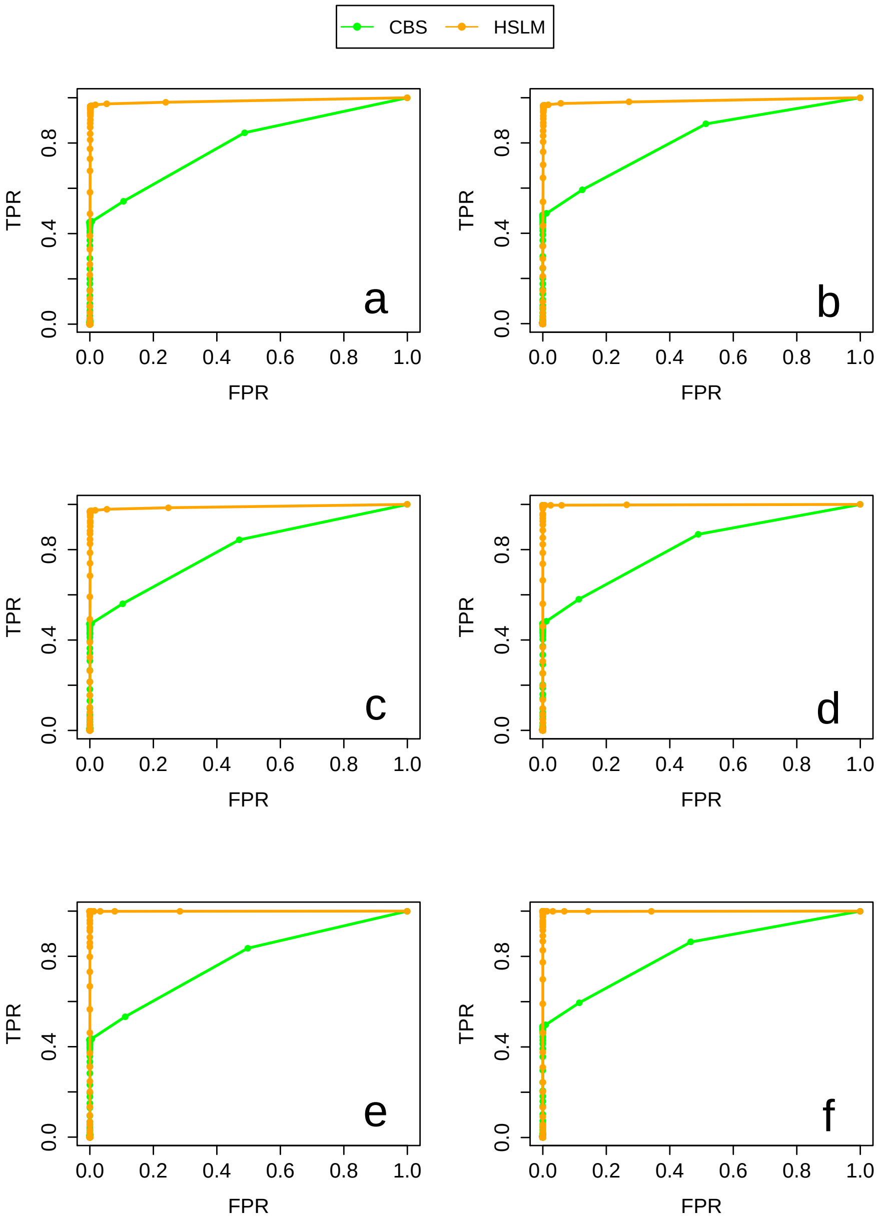

Supplemental Figure 20: Comparison between CBS and HSLM algorithms in the detection of 1-copy alterations made of $N = 20$ exons. The comparison analysis was performed for different values of the distance between adjacent genes ($D = 10Kb$, $D = 50Kb$, $D = 100Kb$, $D = 500Kb$, $D = 1Mb$, $D = 5Mb$). For the HSLM algorith the D_{Norm} was set equal to 10^5. The receiver operating characteristic (ROC) curve was calculated as in Lai *et al.* (2010) and for each of the two algorithms the ROC curve is averaged across 5000 simulations (1000 synthetic chromosome for each g=[1,2,3,4,5]). (a) $D = 10Kb$. (b) $D = 50Kb$. (c) $D = 100Kb$. (d) $D = 500Kb$. (e) $D = 1Mb$. (f) $D = 5Mb$.

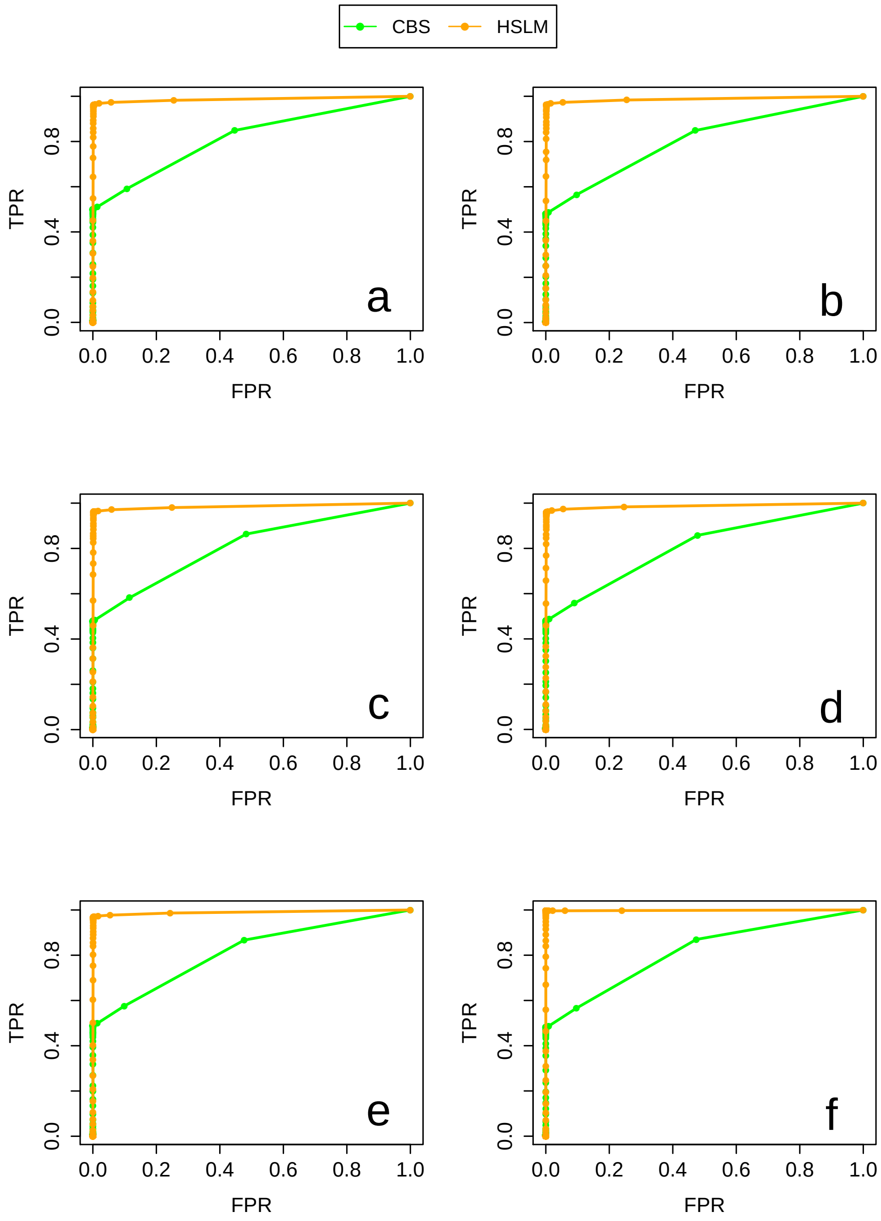

Supplemental Figure 21: Comparison between CBS and HSLM algorithms in the detection of 1-copy alterations made of $N = 20$ exons. The comparison analysis was performed for different values of the distance between adjacent genes ($D = 10Kb$, $D = 50Kb$, $D = 100Kb$, $D = 500Kb$, $D = 1Mb$, $D = 5Mb$). For the HSLM algorith the D_{Norm} was set equal to 10^6. The receiver operating characteristic (ROC) curve was calculated as in Lai *et al.* (2010) and for each of the two algorithms the ROC curve is averaged across 5000 simulations (1000 synthetic chromosome for each g=[1,2,3,4,5]). (a) $D = 10Kb$. (b) $D = 50Kb$. (c) $D = 100Kb$. (d) $D = 500Kb$. (e) $D = 1Mb$. (f) $D = 5Mb$.

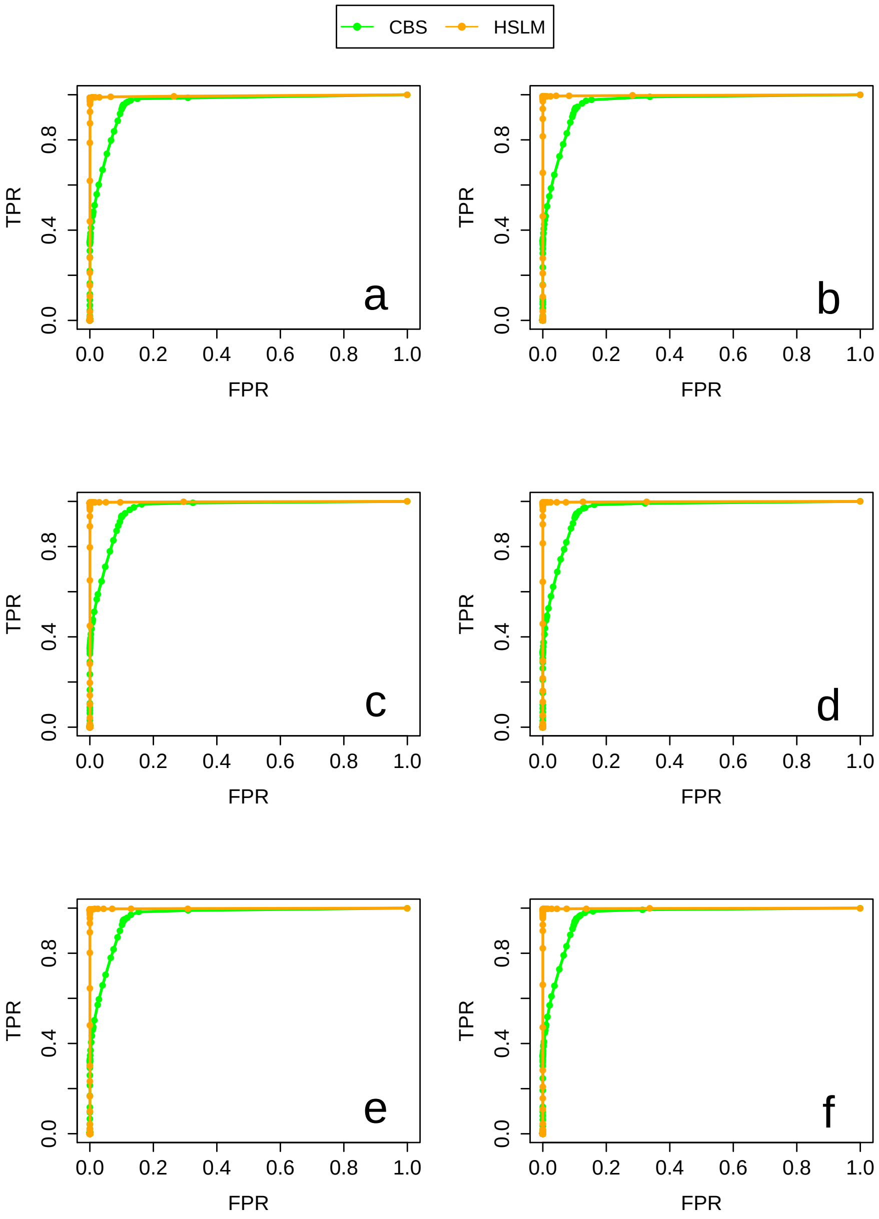

Supplemental Figure 22: Comparison between CBS and HSLM algorithms in the detection of 1-copy alterations made of $N = 50$ exons. The comparison analysis was performed for different values of the distance between adjacent genes ($D = 10Kb$, $D = 50Kb$, $D = 100Kb$, $D = 500Kb$, $D = 1Mb$, $D = 5Mb$). For the HSLM algorith the D_{Norm} was set equal to 10^3. The receiver operating characteristic (ROC) curve was calculated as in Lai *et al.* (2010) and for each of the two algorithms the ROC curve is averaged across 5000 simulations (1000 synthetic chromosome for each g=[1,2,3,4,5]). (a) $D = 10Kb$. (b) $D = 50Kb$. (c) $D = 100Kb$. (d) $D = 500Kb$. (e) $D = 1Mb$. (f) $D = 5Mb$.

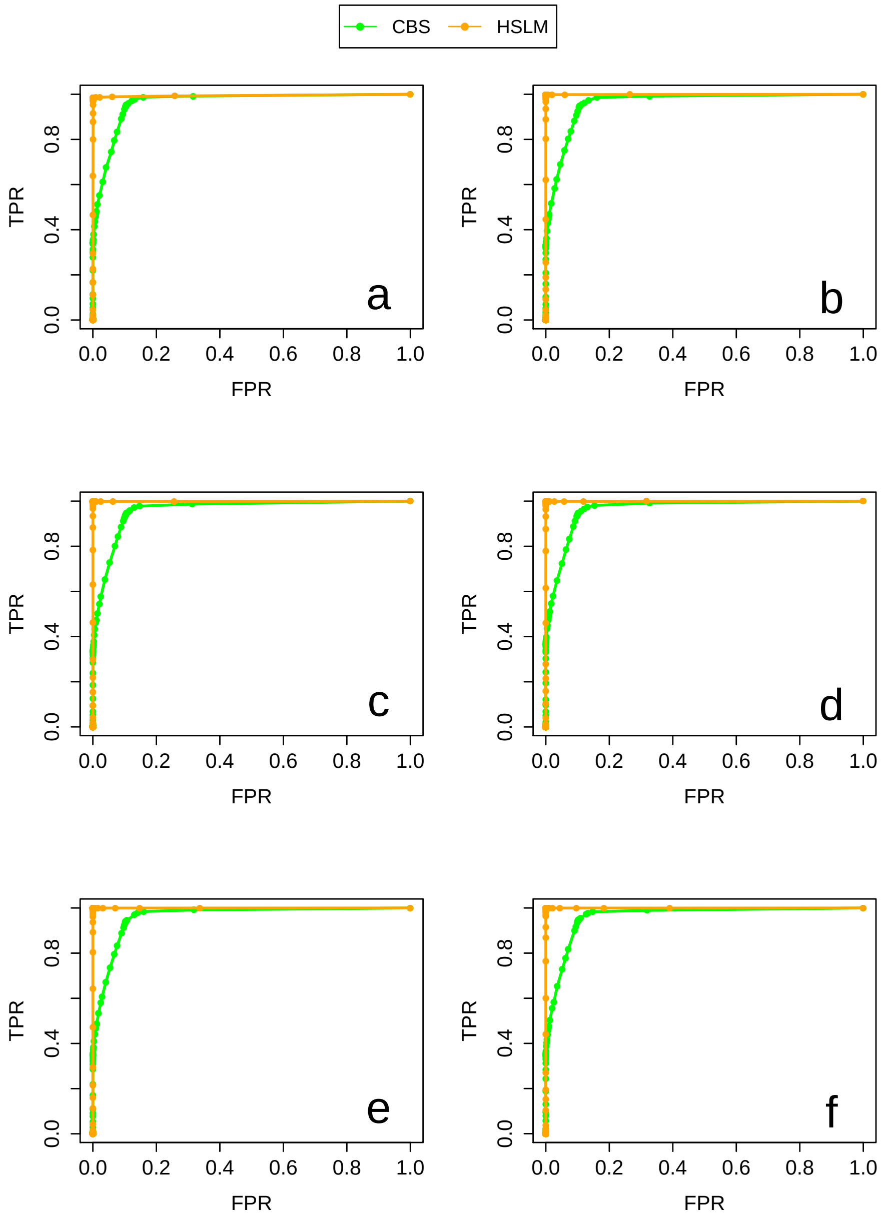

Supplemental Figure 23: Comparison between CBS and HSLM algorithms in the detection of 1-copy alterations made of $N = 50$ exons. The comparison analysis was performed for different values of the distance between adjacent genes ($D = 10Kb$, $D = 50Kb$, $D = 100Kb$, $D = 500Kb$, $D = 1Mb$, $D = 5Mb$). For the HSLM algorith the D_{Norm} was set equal to 10^4. The receiver operating characteristic (ROC) curve was calculated as in Lai *et al.* (2010) and for each of the two algorithms the ROC curve is averaged across 5000 simulations (1000 synthetic chromosome for each g=[1,2,3,4,5]). (a) $D = 10Kb$. (b) $D = 50Kb$. (c) $D = 100Kb$. (d) $D = 500Kb$. (e) $D = 1Mb$. (f) $D = 5Mb$.

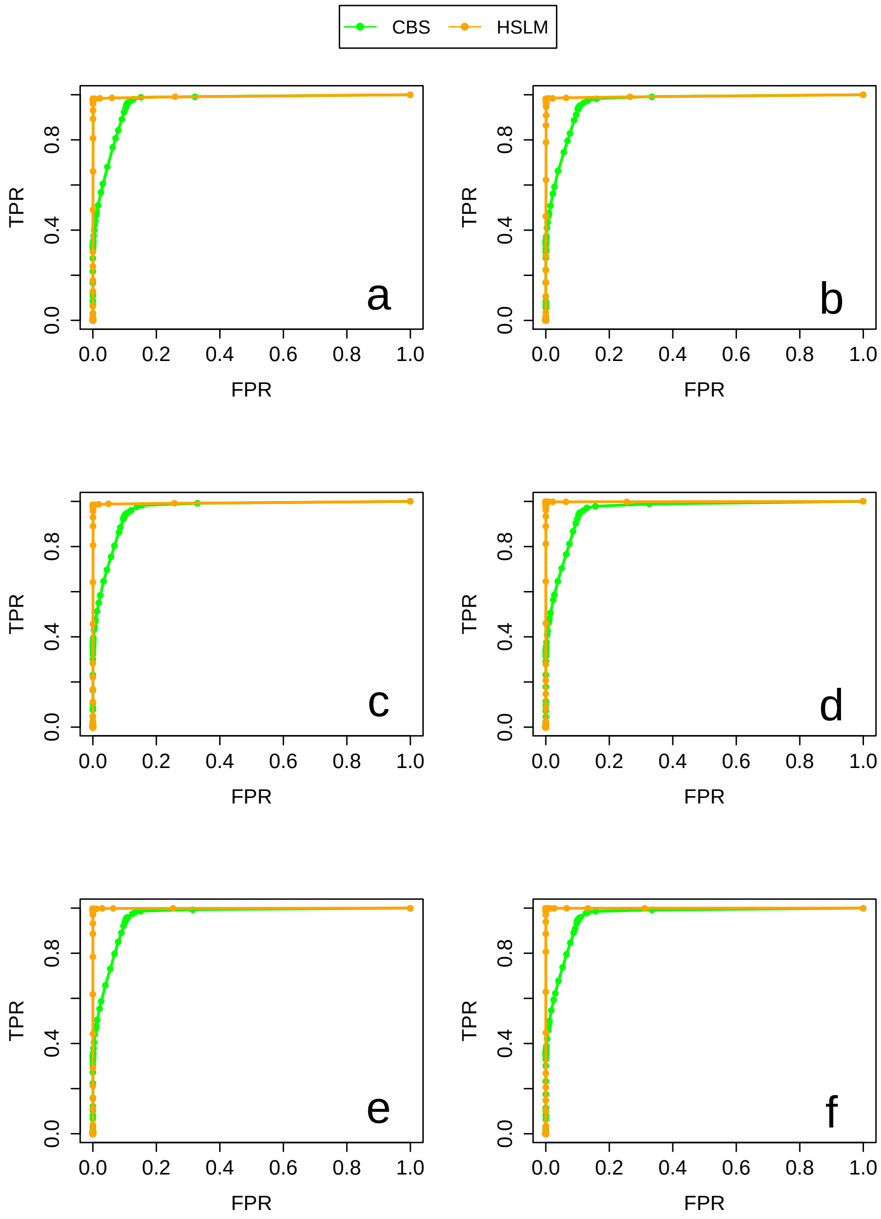

Supplemental Figure 24: Comparison between CBS and HSLM algorithms in the detection of 1-copy alterations made of $N = 50$ exons. The comparison analysis was performed for different values of the distance between adjacent genes ($D = 10Kb$, $D = 50Kb$, $D = 100Kb$, $D = 500Kb$, $D = 1Mb$, $D = 5Mb$). For the HSLM algorith the D_{Norm} was set equal to 10^5. The receiver operating characteristic (ROC) curve was calculated as in Lai *et al.* (2010) and for each of the two algorithms the ROC curve is averaged across 5000 simulations (1000 synthetic chromosome for each g=[1,2,3,4,5]). (a) $D = 10Kb$. (b) $D = 50Kb$. (c) $D = 100Kb$. (d) $D = 500Kb$. (e) $D = 1Mb$. (f) $D = 5Mb$.

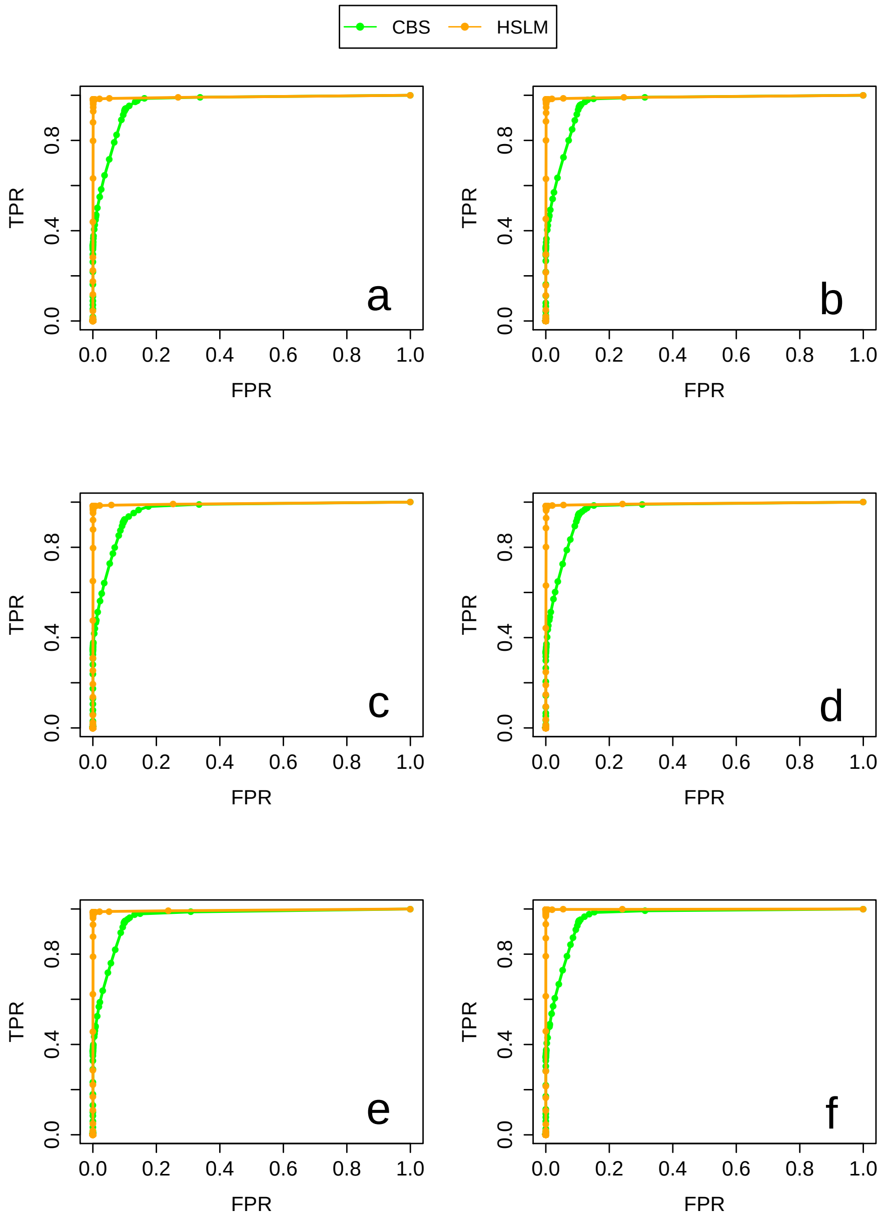

Supplemental Figure 25: Comparison between CBS and HSLM algorithms in the detection of 1-copy alterations made of $N = 50$ exons. The comparison analysis was performed for different values of the distance between adjacent genes ($D = 10Kb$, $D = 50Kb$, $D = 100Kb$, $D = 500Kb$, $D = 1Mb$, $D = 5Mb$). For the HSLM algorith the D_{Norm} was set equal to 10^6. The receiver operating characteristic (ROC) curve was calculated as in Lai *et al.* (2010) and for each of the two algorithms the ROC curve is averaged across 5000 simulations (1000 synthetic chromosome for each g=[1,2,3,4,5]). (a) $D = 10Kb$. (b) $D = 50Kb$. (c) $D = 100Kb$. (d) $D = 500Kb$. (e) $D = 1Mb$. (f) $D = 5Mb$.

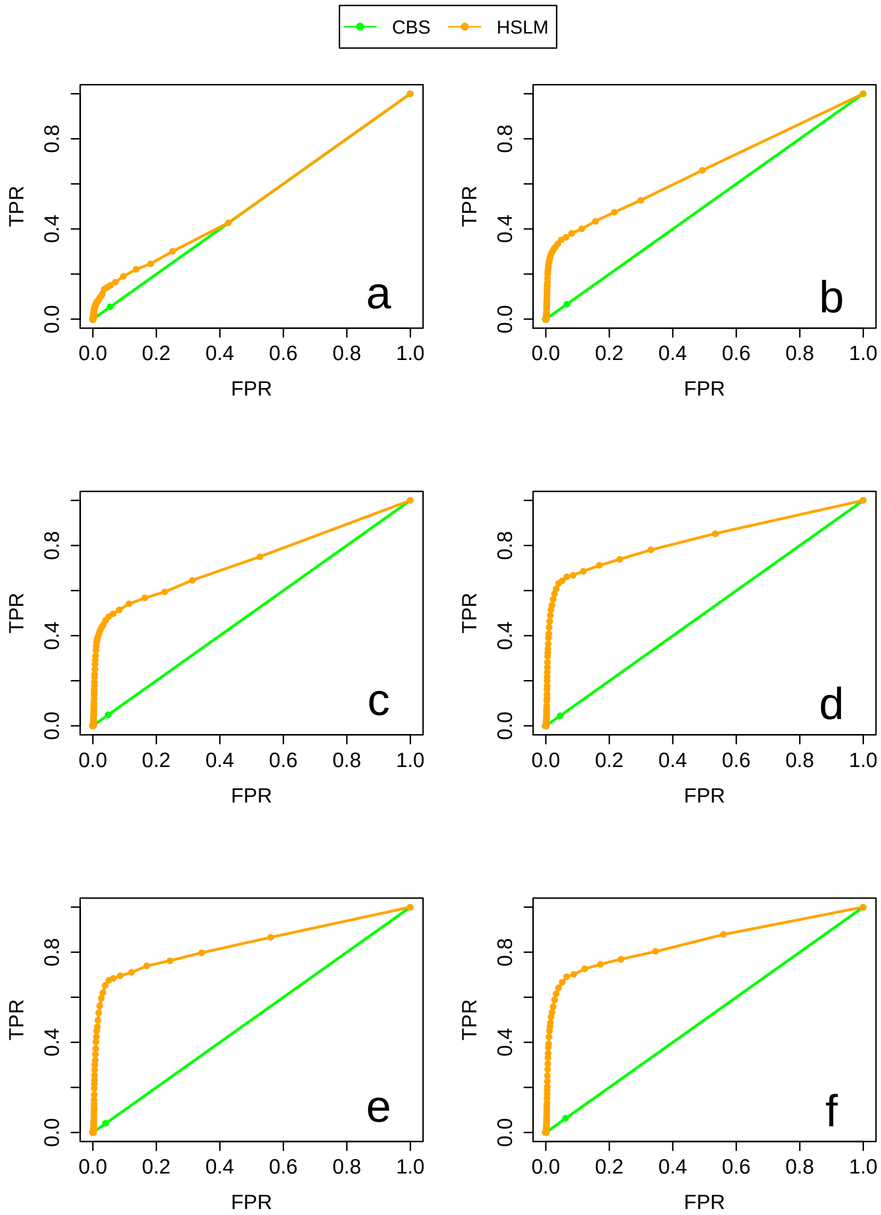

Supplemental Figure 26: Comparison between CBS and HSLM algorithms in the detection of 3-copy alterations made of $N = 2$ exons. The comparison analysis was performed for different values of the distance between adjacent genes ($D = 10Kb$, $D = 50Kb$, $D = 100Kb$, $D = 500Kb$, $D = 1Mb$, $D = 5Mb$). For the HSLM algorith the D_{Norm} was set equal to 10^3. The receiver operating characteristic (ROC) curve was calculated as in Lai *et al.* (2010) and for each of the two algorithms the ROC curve is averaged across 5000 simulations (1000 synthetic chromosome for each g=[1,2,3,4,5]). (a) $D = 10Kb$. (b) $D = 50Kb$. (c) $D = 100Kb$. (d) $D = 500Kb$. (e) $D = 1Mb$. (f) $D = 5Mb$.

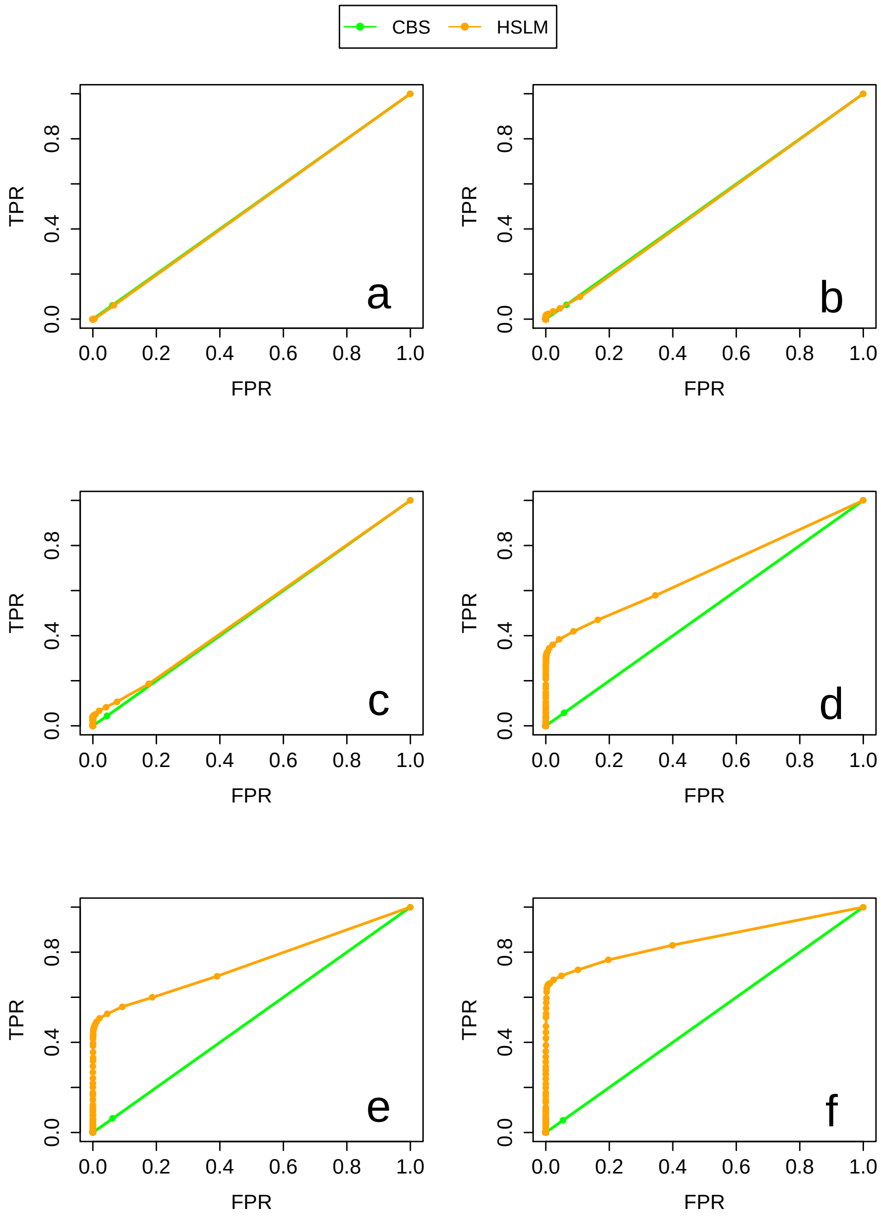

Supplemental Figure 27: Comparison between CBS and HSLM algorithms in the detection of 3-copy alterations made of $N = 2$ exons. The comparison analysis was performed for different values of the distance between adjacent genes ($D = 10Kb$, $D = 50Kb$, $D = 100Kb$, $D = 500Kb$, $D = 1Mb$, $D = 5Mb$). For the HSLM algorith the D_{Norm} was set equal to 10^4. The receiver operating characteristic (ROC) curve was calculated as in Lai *et al.* (2010) and for each of the two algorithms the ROC curve is averaged across 5000 simulations (1000 synthetic chromosome for each g=[1,2,3,4,5]). (a) $D = 10Kb$. (b) $D = 50Kb$. (c) $D = 100Kb$. (d) $D = 500Kb$. (e) $D = 1Mb$. (f) $D = 5Mb$.

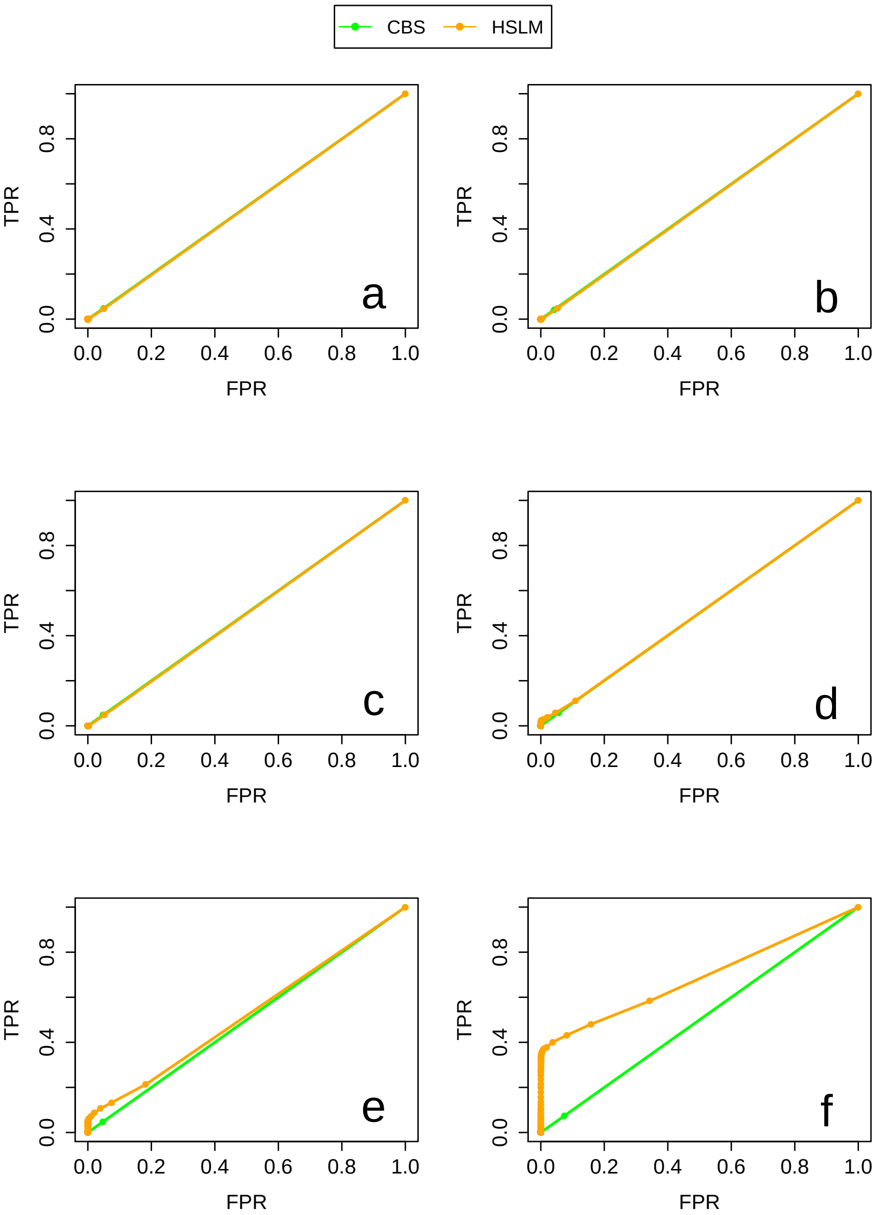

Supplemental Figure 28: Comparison between CBS and HSLM algorithms in the detection of 3-copy alterations made of $N = 2$ exons. The comparison analysis was performed for different values of the distance between adjacent genes ($D = 10Kb$, $D = 50Kb$, $D = 100Kb$, $D = 500Kb$, $D = 1Mb$, $D = 5Mb$). For the HSLM algorith the D_{Norm} was set equal to 10^5. The receiver operating characteristic (ROC) curve was calculated as in Lai *et al.* (2010) and for each of the two algorithms the ROC curve is averaged across 5000 simulations (1000 synthetic chromosome for each g=[1,2,3,4,5]). (a) $D = 10Kb$. (b) $D = 50Kb$. (c) $D = 100Kb$. (d) $D = 500Kb$. (e) $D = 1Mb$. (f) $D = 5Mb$.

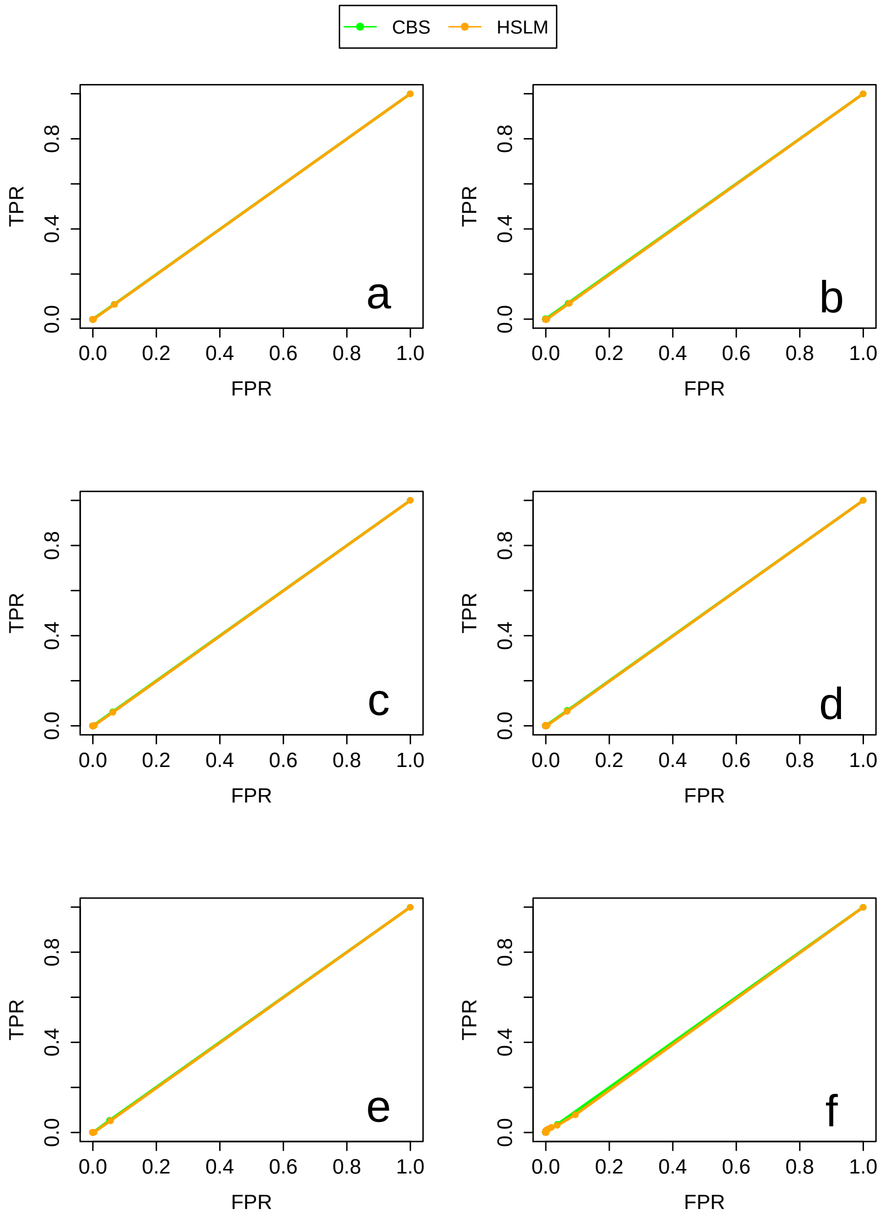

Supplemental Figure 29: Comparison between CBS and HSLM algorithms in the detection of 3-copy alterations made of $N = 2$ exons. The comparison analysis was performed for different values of the distance between adjacent genes ($D = 10Kb$, $D = 50Kb$, $D = 100Kb$, $D = 500Kb$, $D = 1Mb$, $D = 5Mb$). For the HSLM algorith the D_{Norm} was set equal to 10^6. The receiver operating characteristic (ROC) curve was calculated as in Lai *et al.* (2010) and for each of the two algorithms the ROC curve is averaged across 5000 simulations (1000 synthetic chromosome for each g=[1,2,3,4,5]). (a) $D = 10Kb$. (b) $D = 50Kb$. (c) $D = 100Kb$. (d) $D = 500Kb$. (e) $D = 1Mb$. (f) $D = 5Mb$.

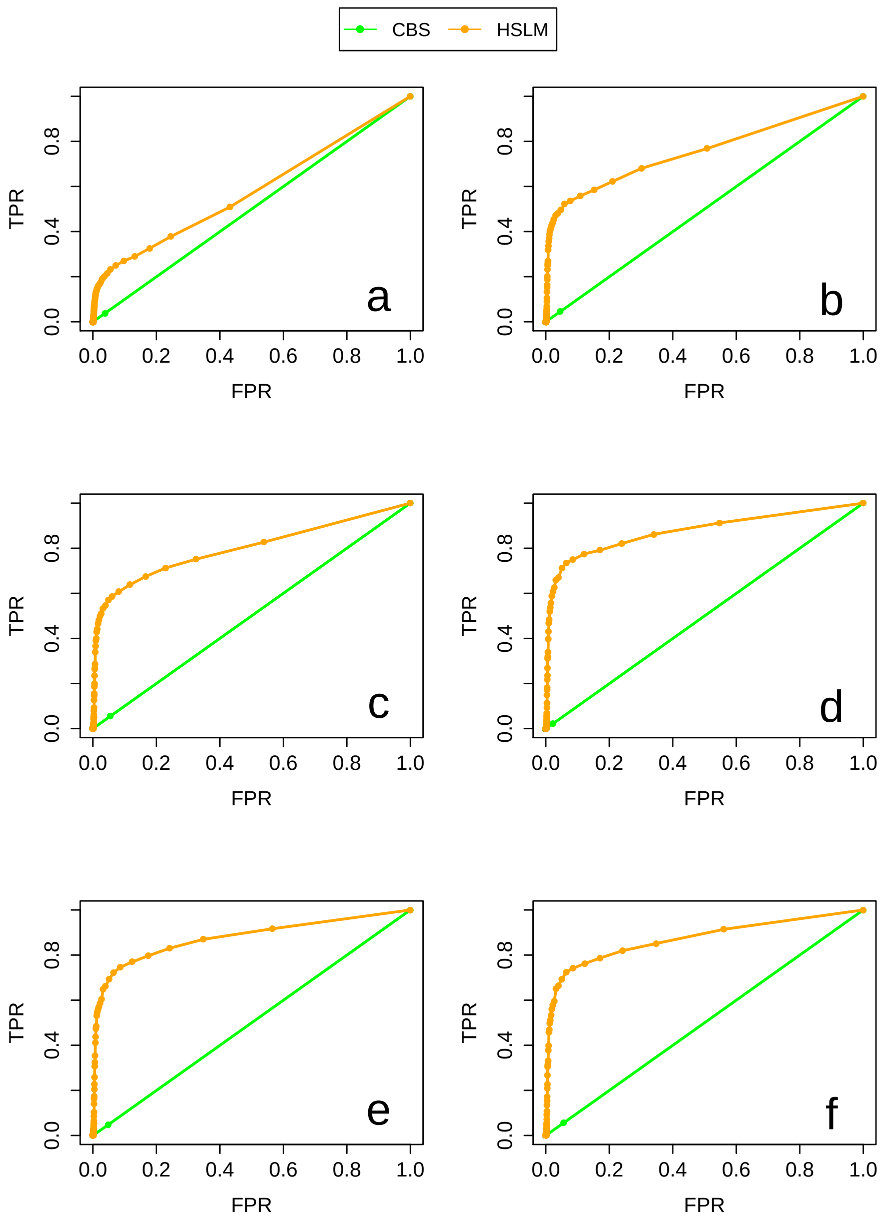

Supplemental Figure 30: Comparison between CBS and HSLM algorithms in the detection of 3-copy alterations made of $N = 3$ exons. The comparison analysis was performed for different values of the distance between adjacent genes ($D = 10Kb$, $D = 50Kb$, $D = 100Kb$, $D = 500Kb$, $D = 1Mb$, $D = 5Mb$). For the HSLM algorith the D_{Norm} was set equal to 10^3. The receiver operating characteristic (ROC) curve was calculated as in Lai *et al.* (2010) and for each of the two algorithms the ROC curve is averaged across 5000 simulations (1000 synthetic chromosome for each g=[1,2,3,4,5]). (a) $D = 10Kb$. (b) $D = 50Kb$. (c) $D = 100Kb$. (d) $D = 500Kb$. (e) $D = 1Mb$. (f) $D = 5Mb$.

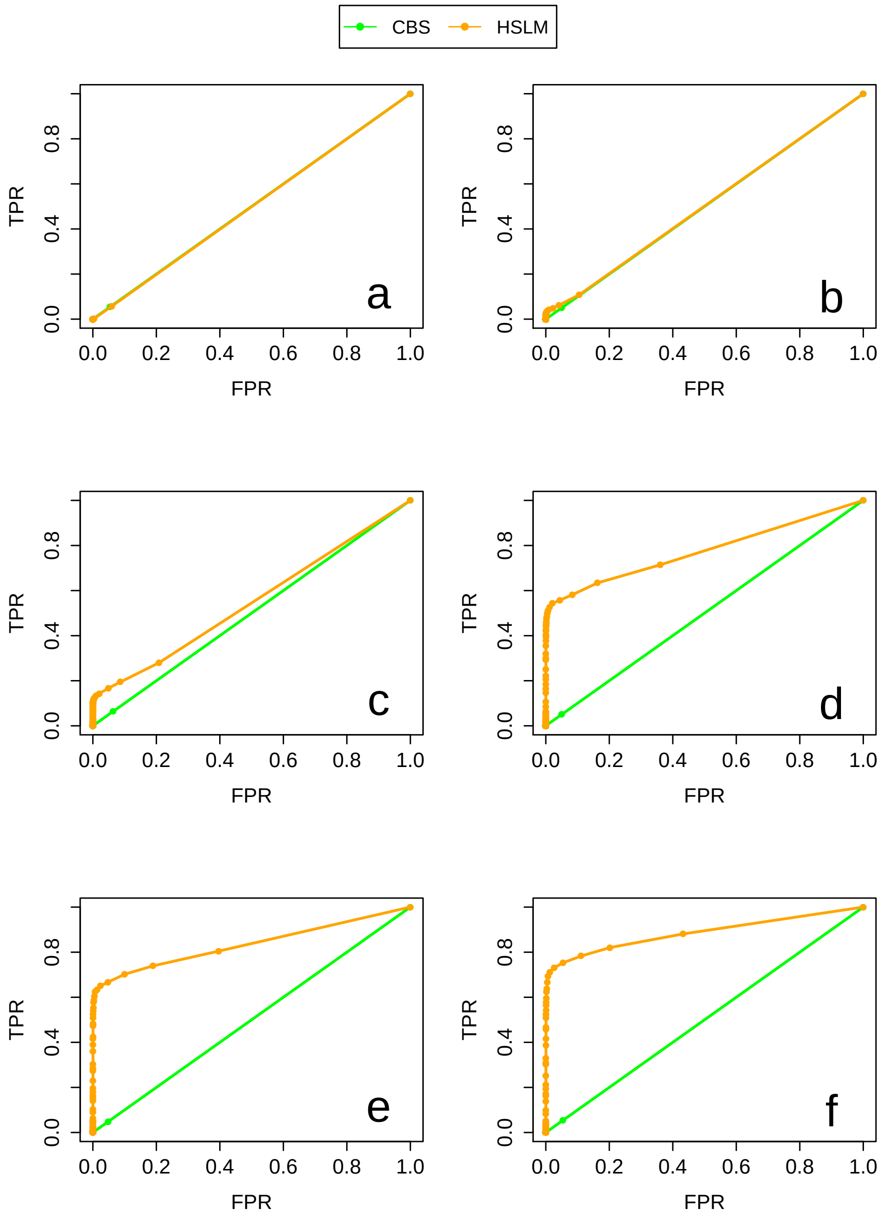

Supplemental Figure 31: Comparison between CBS and HSLM algorithms in the detection of 3-copy alterations made of $N = 3$ exons. The comparison analysis was performed for different values of the distance between adjacent genes ($D = 10Kb$, $D = 50Kb$, $D = 100Kb$, $D = 500Kb$, $D = 1Mb$, $D = 5Mb$). For the HSLM algorith the D_{Norm} was set equal to 10^4. The receiver operating characteristic (ROC) curve was calculated as in Lai *et al.* (2010) and for each of the two algorithms the ROC curve is averaged across 5000 simulations (1000 synthetic chromosome for each g=[1,2,3,4,5]). (a) $D = 10Kb$. (b) $D = 50Kb$. (c) $D = 100Kb$. (d) $D = 500Kb$. (e) $D = 1Mb$. (f) $D = 5Mb$.

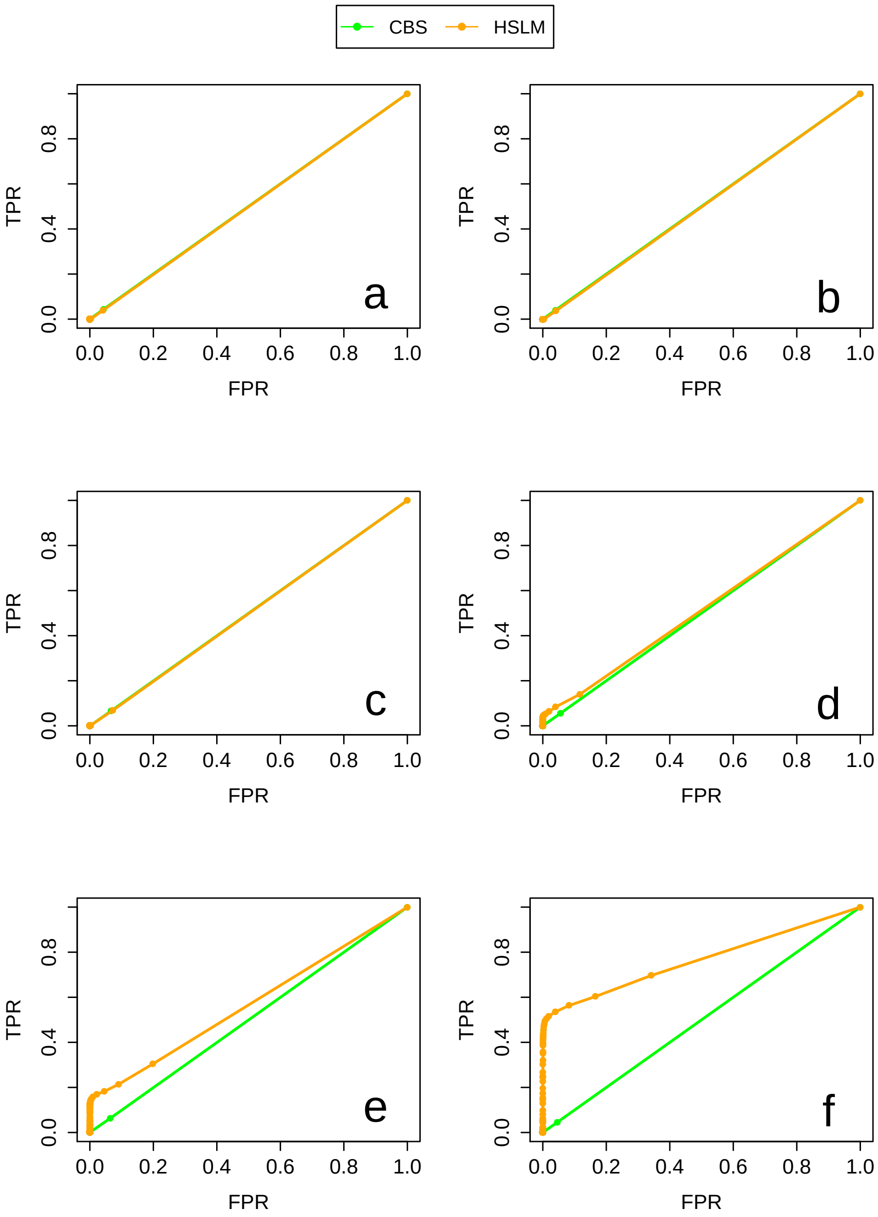

Supplemental Figure 32: Comparison between CBS and HSLM algorithms in the detection of 3-copy alterations made of $N = 3$ exons. The comparison analysis was performed for different values of the distance between adjacent genes ($D = 10Kb$, $D = 50Kb$, $D = 100Kb$, $D = 500Kb$, $D = 1Mb$, $D = 5Mb$). For the HSLM algorith the D_{Norm} was set equal to 10^5. The receiver operating characteristic (ROC) curve was calculated as in Lai *et al.* (2010) and for each of the two algorithms the ROC curve is averaged across 5000 simulations (1000 synthetic chromosome for each g=[1,2,3,4,5]). (a) $D = 10Kb$. (b) $D = 50Kb$. (c) $D = 100Kb$. (d) $D = 500Kb$. (e) $D = 1Mb$. (f) $D = 5Mb$.

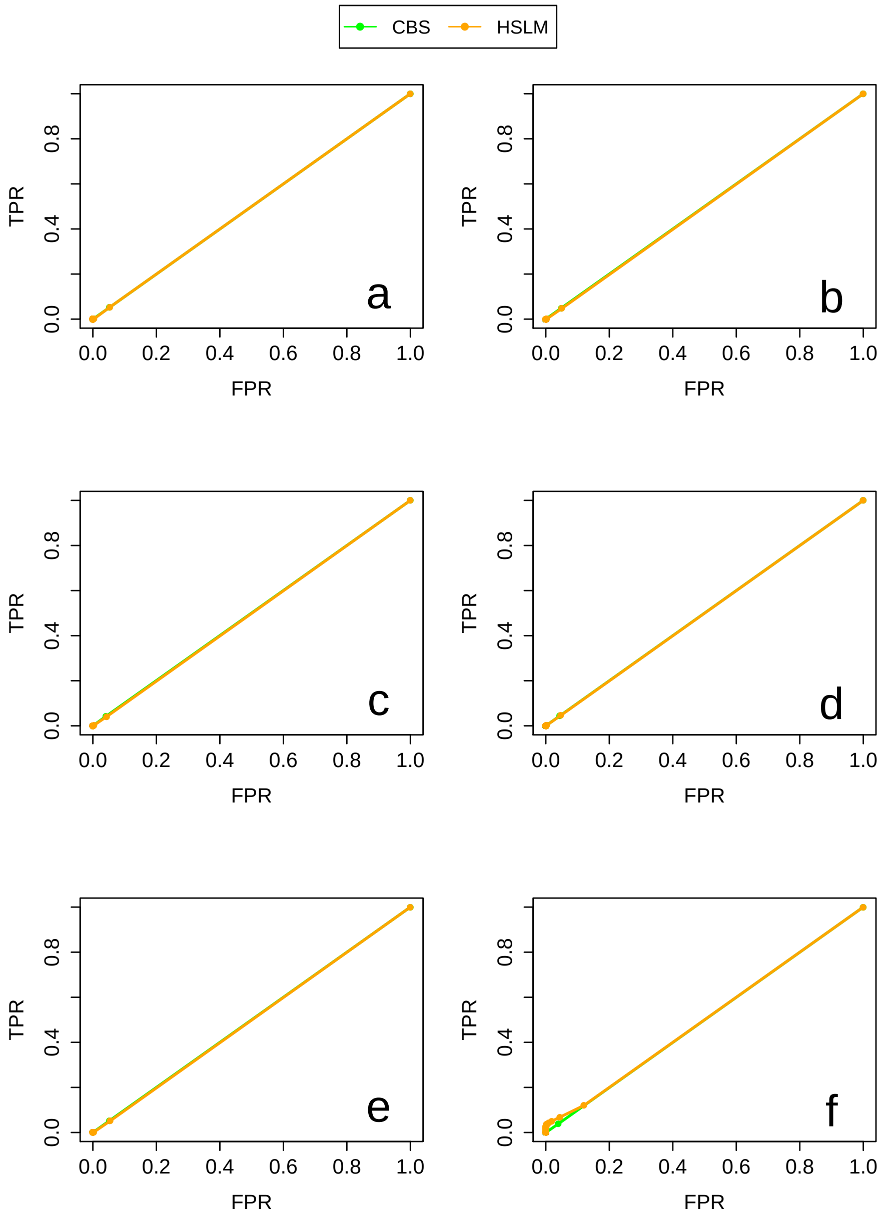

Supplemental Figure 33: Comparison between CBS and HSLM algorithms in the detection of 3-copy alterations made of $N = 3$ exons. The comparison analysis was performed for different values of the distance between adjacent genes ($D = 10Kb$, $D = 50Kb$, $D = 100Kb$, $D = 500Kb$, $D = 1Mb$, $D = 5Mb$). For the HSLM algorith the D_{Norm} was set equal to 10^6. The receiver operating characteristic (ROC) curve was calculated as in Lai *et al.* (2010) and for each of the two algorithms the ROC curve is averaged across 5000 simulations (1000 synthetic chromosome for each g=[1,2,3,4,5]). (a) $D = 10Kb$. (b) $D = 50Kb$. (c) $D = 100Kb$. (d) $D = 500Kb$. (e) $D = 1Mb$. (f) $D = 5Mb$.

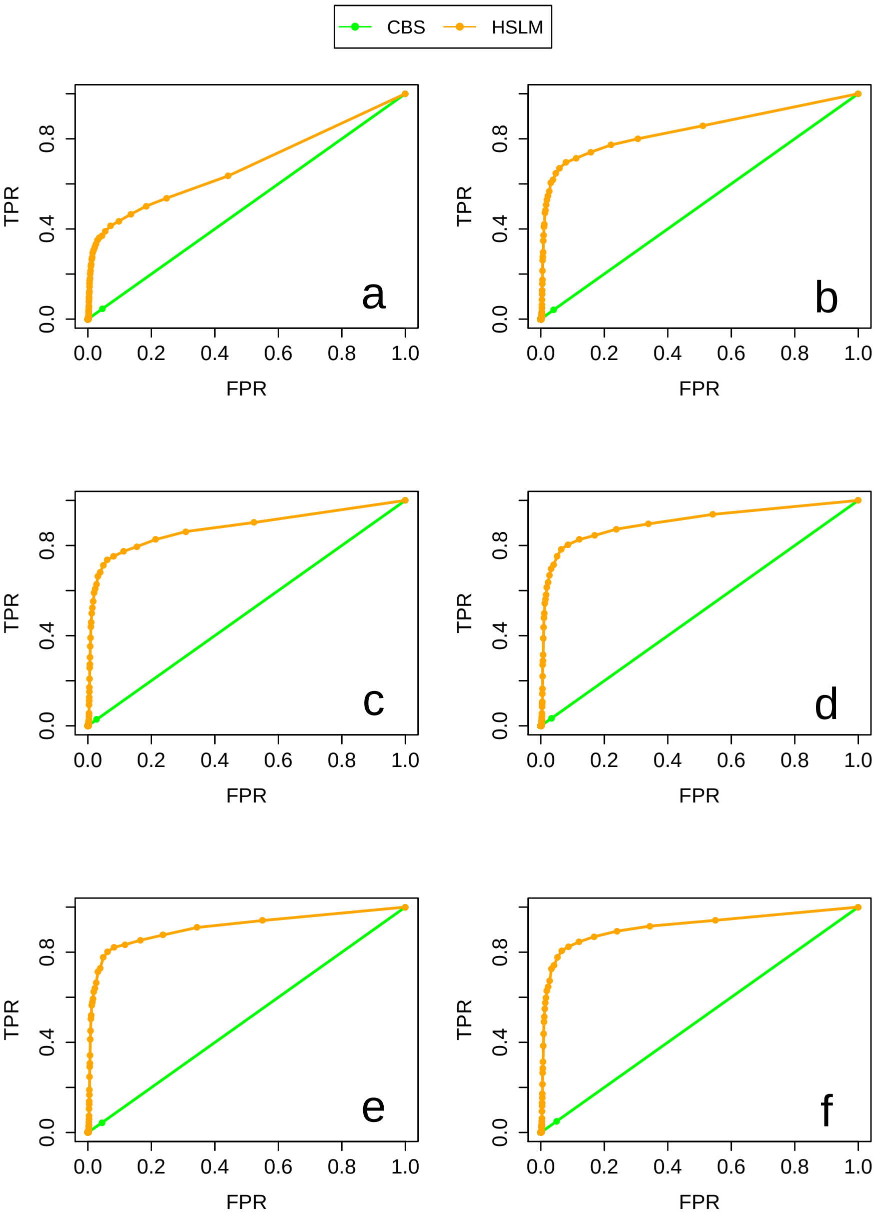

Supplemental Figure 34: Comparison between CBS and HSLM algorithms in the detection of 3-copy alterations made of $N = 5$ exons. The comparison analysis was performed for different values of the distance between adjacent genes ($D = 10Kb$, $D = 50Kb$, $D = 100Kb$, $D = 500Kb$, $D = 1Mb$, $D = 5Mb$). For the HSLM algorith the D_{Norm} was set equal to 10^3. The receiver operating characteristic (ROC) curve was calculated as in Lai *et al.* (2010) and for each of the two algorithms the ROC curve is averaged across 5000 simulations (1000 synthetic chromosome for each g=[1,2,3,4,5]). (a) $D = 10Kb$. (b) $D = 50Kb$. (c) $D = 100Kb$. (d) $D = 500Kb$. (e) $D = 1Mb$. (f) $D = 5Mb$.

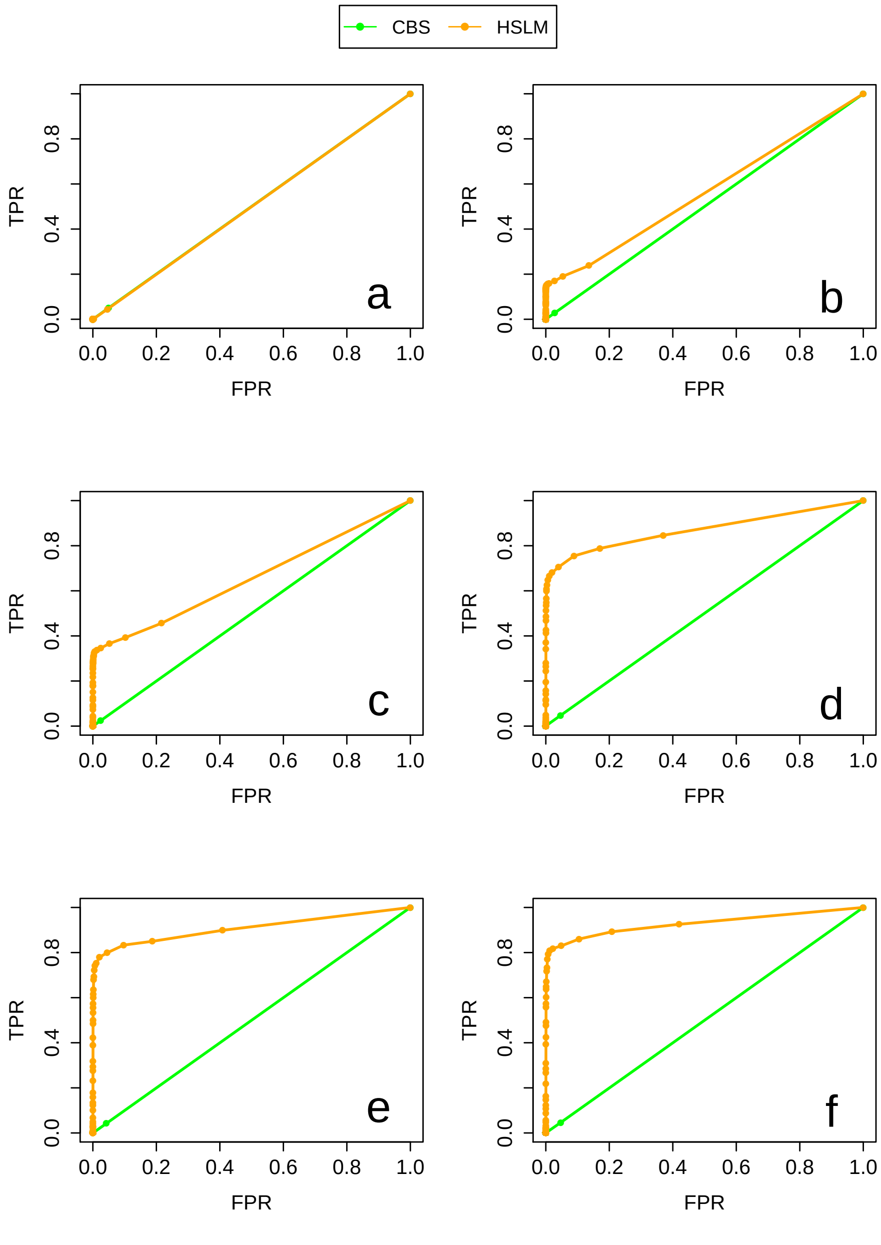

Supplemental Figure 35: Comparison between CBS and HSLM algorithms in the detection of 3-copy alterations made of $N = 5$ exons. The comparison analysis was performed for different values of the distance between adjacent genes ($D = 10Kb$, $D = 50Kb$, $D = 100Kb$, $D = 500Kb$, $D = 1Mb$, $D = 5Mb$). For the HSLM algorith the D_{Norm} was set equal to 10^4. The receiver operating characteristic (ROC) curve was calculated as in Lai *et al.* (2010) and for each of the two algorithms the ROC curve is averaged across 5000 simulations (1000 synthetic chromosome for each g=[1,2,3,4,5]). (a) $D = 10Kb$. (b) $D = 50Kb$. (c) $D = 100Kb$. (d) $D = 500Kb$. (e) $D = 1Mb$. (f) $D = 5Mb$.

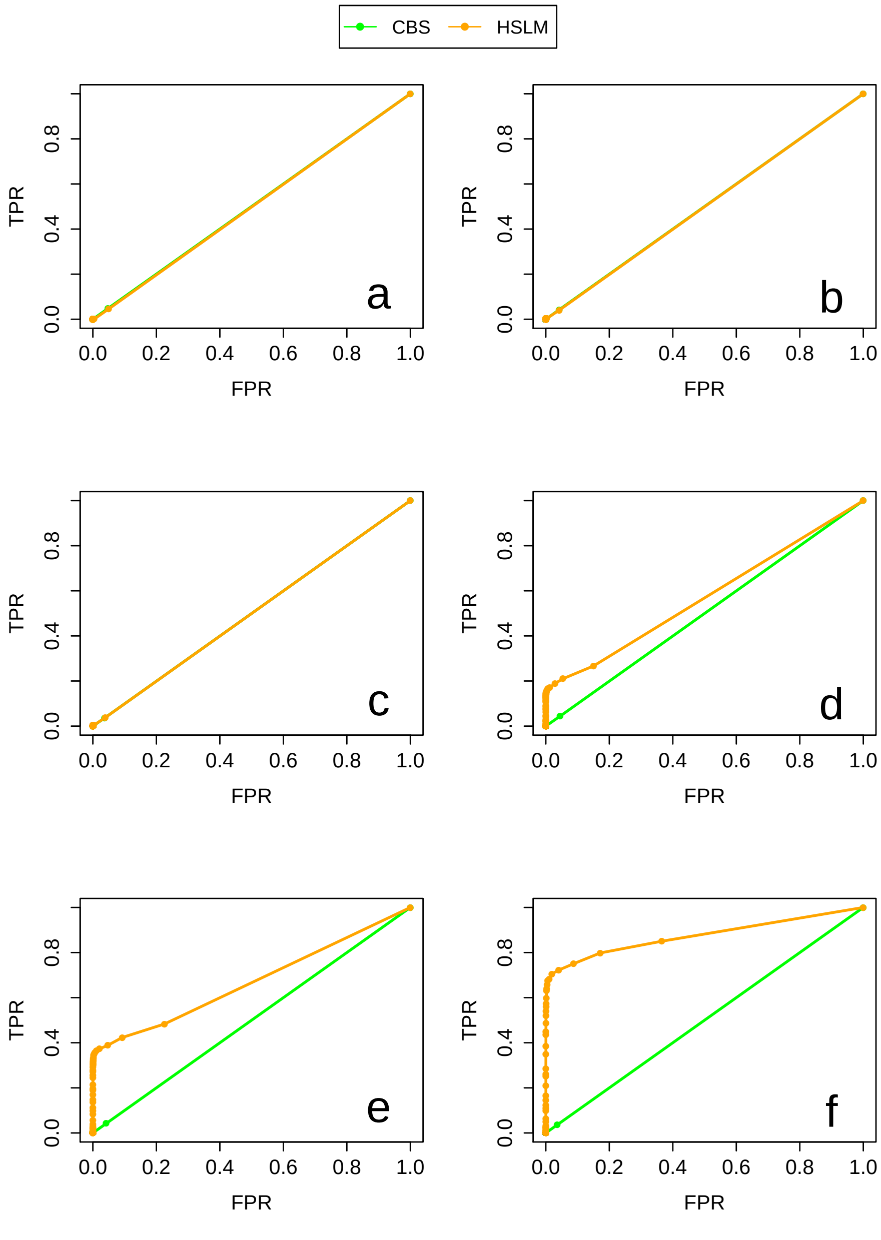

Supplemental Figure 36: Comparison between CBS and HSLM algorithms in the detection of 3-copy alterations made of $N = 5$ exons. The comparison analysis was performed for different values of the distance between adjacent genes ($D = 10Kb$, $D = 50Kb$, $D = 100Kb$, $D = 500Kb$, $D = 1Mb$, $D = 5Mb$). For the HSLM algorith the D_{Norm} was set equal to 10^5. The receiver operating characteristic (ROC) curve was calculated as in Lai *et al.* (2010) and for each of the two algorithms the ROC curve is averaged across 5000 simulations (1000 synthetic chromosome for each g=[1,2,3,4,5]). (a) $D = 10Kb$. (b) $D = 50Kb$. (c) $D = 100Kb$. (d) $D = 500Kb$. (e) $D = 1Mb$. (f) $D = 5Mb$.

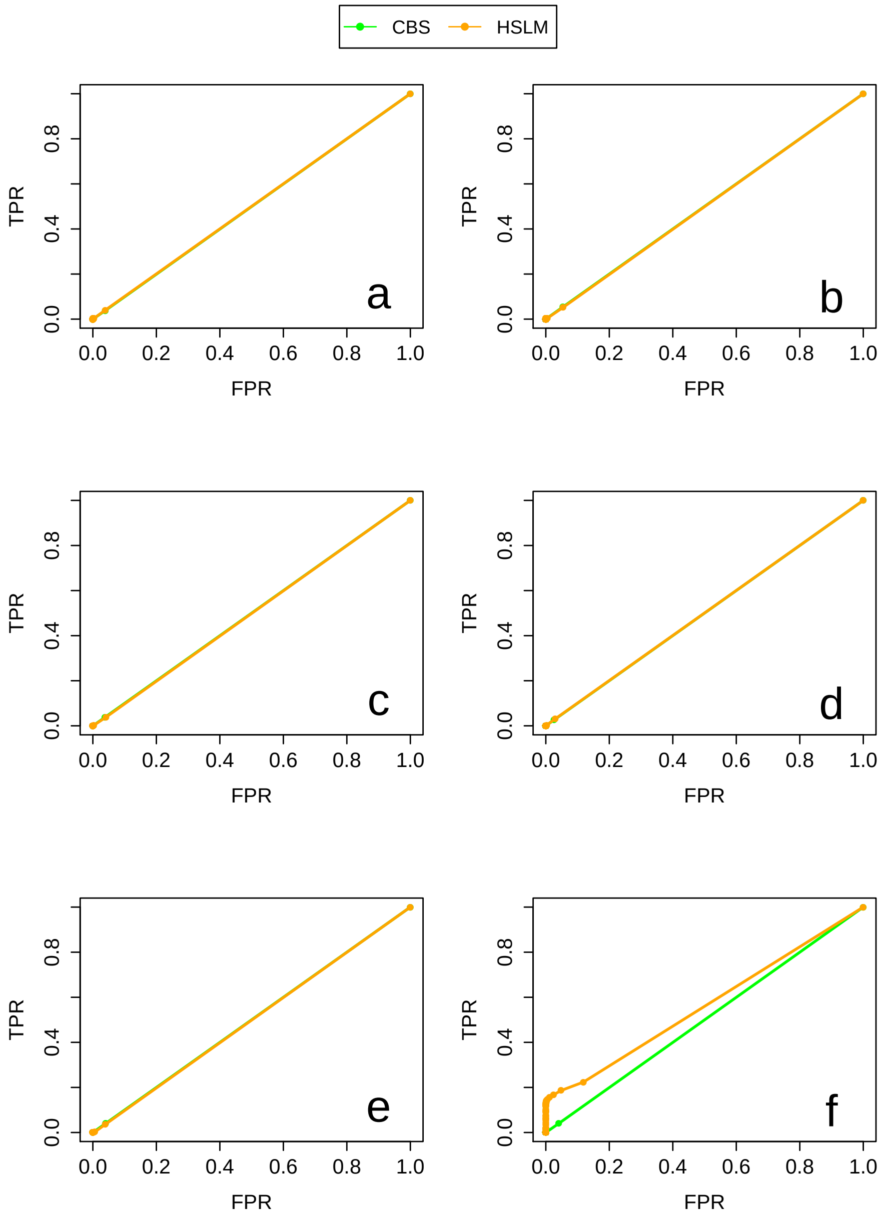

Supplemental Figure 37: Comparison between CBS and HSLM algorithms in the detection of 3-copy alterations made of $N = 5$ exons. The comparison analysis was performed for different values of the distance between adjacent genes ($D = 10Kb$, $D = 50Kb$, $D = 100Kb$, $D = 500Kb$, $D = 1Mb$, $D = 5Mb$). For the HSLM algorith the D_{Norm} was set equal to 10^6. The receiver operating characteristic (ROC) curve was calculated as in Lai *et al.* (2010) and for each of the two algorithms the ROC curve is averaged across 5000 simulations (1000 synthetic chromosome for each g=[1,2,3,4,5]). (a) $D = 10Kb$. (b) $D = 50Kb$. (c) $D = 100Kb$. (d) $D = 500Kb$. (e) $D = 1Mb$. (f) $D = 5Mb$.

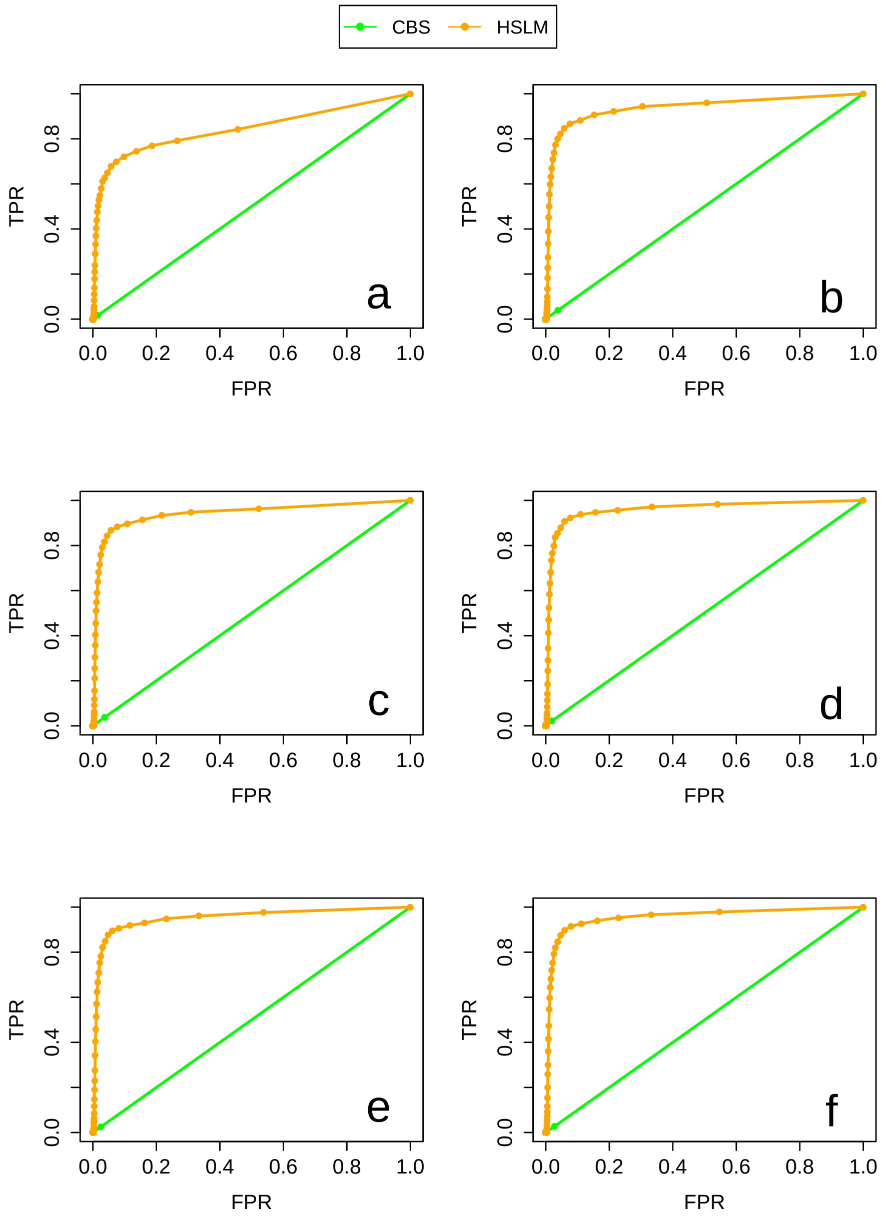

Supplemental Figure 38: Comparison between CBS and HSLM algorithms in the detection of 3-copy alterations made of $N = 10$ exons. The comparison analysis was performed for different values of the distance between adjacent genes ($D = 10Kb$, $D = 50Kb$, $D = 100Kb$, $D = 500Kb$, $D = 1Mb$, $D = 5Mb$). For the HSLM algorith the D_{Norm} was set equal to 10^3. The receiver operating characteristic (ROC) curve was calculated as in Lai *et al.* (2010) and for each of the two algorithms the ROC curve is averaged across 5000 simulations (1000 synthetic chromosome for each g=[1,2,3,4,5]). (a) $D = 10Kb$. (b) $D = 50Kb$. (c) $D = 100Kb$. (d) $D = 500Kb$. (e) $D = 1Mb$. (f) $D = 5Mb$.

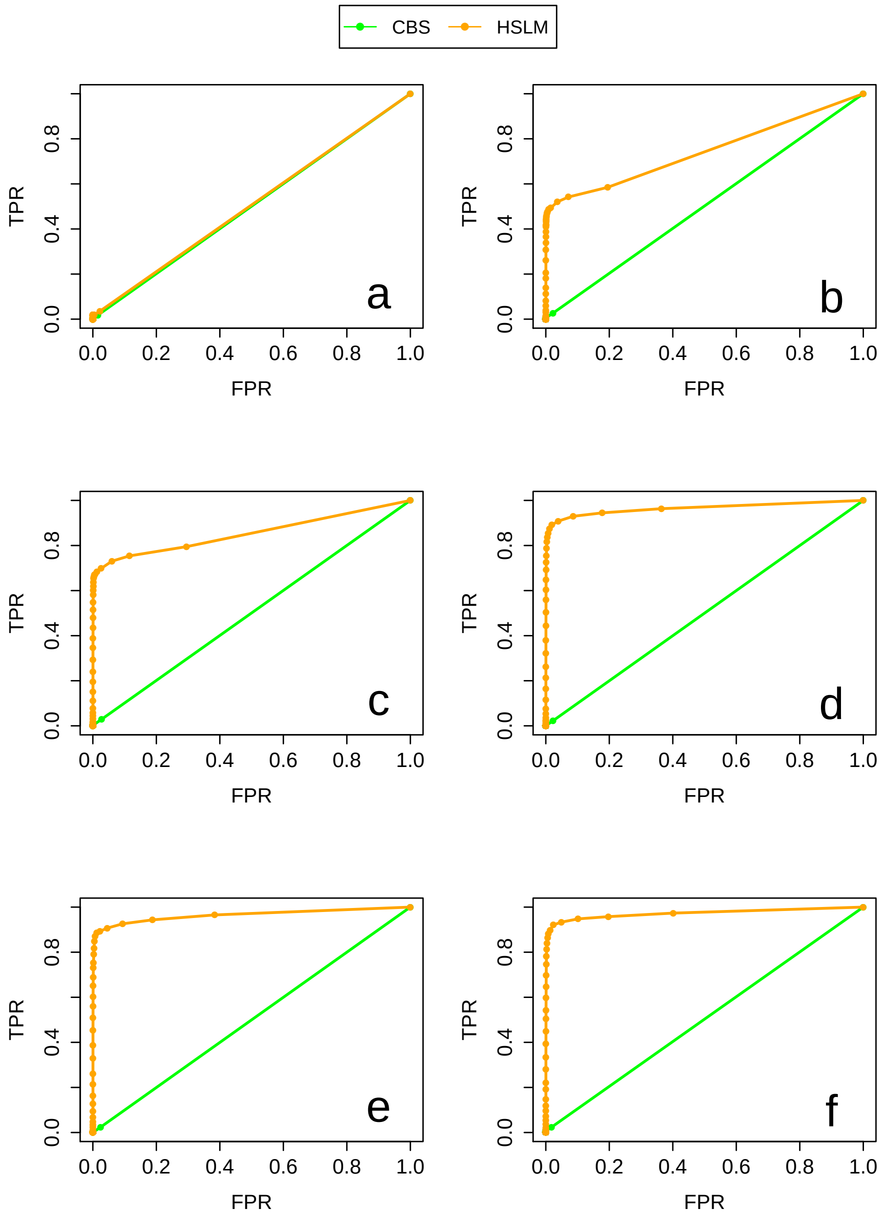

Supplemental Figure 39: Comparison between CBS and HSLM algorithms in the detection of 3-copy alterations made of $N = 10$ exons. The comparison analysis was performed for different values of the distance between adjacent genes ($D = 10Kb$, $D = 50Kb$, $D = 100Kb$, $D = 500Kb$, $D = 1Mb$, $D = 5Mb$). For the HSLM algorith the D_{Norm} was set equal to 10^4. The receiver operating characteristic (ROC) curve was calculated as in Lai *et al.* (2010) and for each of the two algorithms the ROC curve is averaged across 5000 simulations (1000 synthetic chromosome for each g=[1,2,3,4,5]). (a) $D = 10Kb$. (b) $D = 50Kb$. (c) $D = 100Kb$. (d) $D = 500Kb$. (e) $D = 1Mb$. (f) $D = 5Mb$.

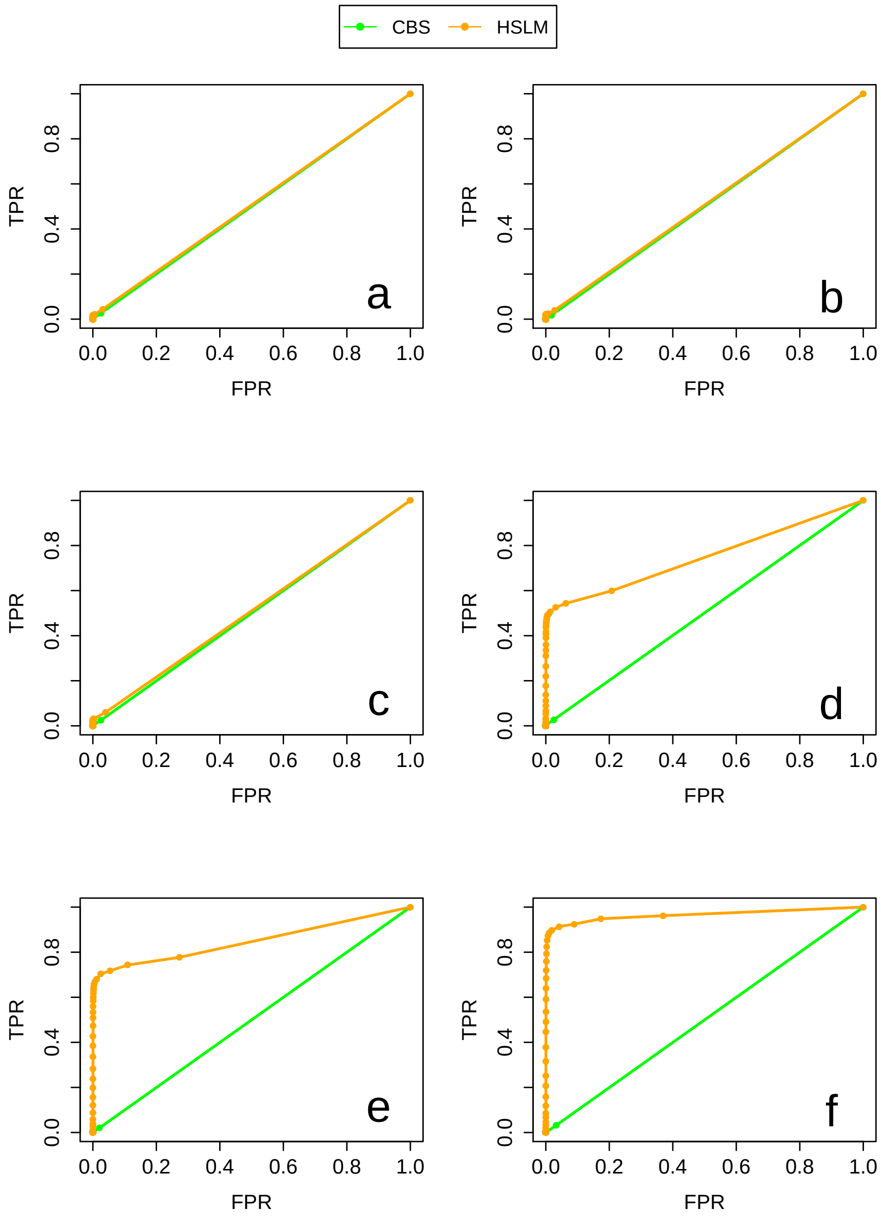

Supplemental Figure 40: Comparison between CBS and HSLM algorithms in the detection of 3-copy alterations made of $N = 10$ exons. The comparison analysis was performed for different values of the distance between adjacent genes ($D = 10Kb$, $D = 50Kb$, $D = 100Kb$, $D = 500Kb$, $D = 1Mb$, $D = 5Mb$). For the HSLM algorith the D_{Norm} was set equal to 10^5. The receiver operating characteristic (ROC) curve was calculated as in Lai *et al.* (2010) and for each of the two algorithms the ROC curve is averaged across 5000 simulations (1000 synthetic chromosome for each g=[1,2,3,4,5]). (a) $D = 10Kb$. (b) $D = 50Kb$. (c) $D = 100Kb$. (d) $D = 500Kb$. (e) $D = 1Mb$. (f) $D = 5Mb$.

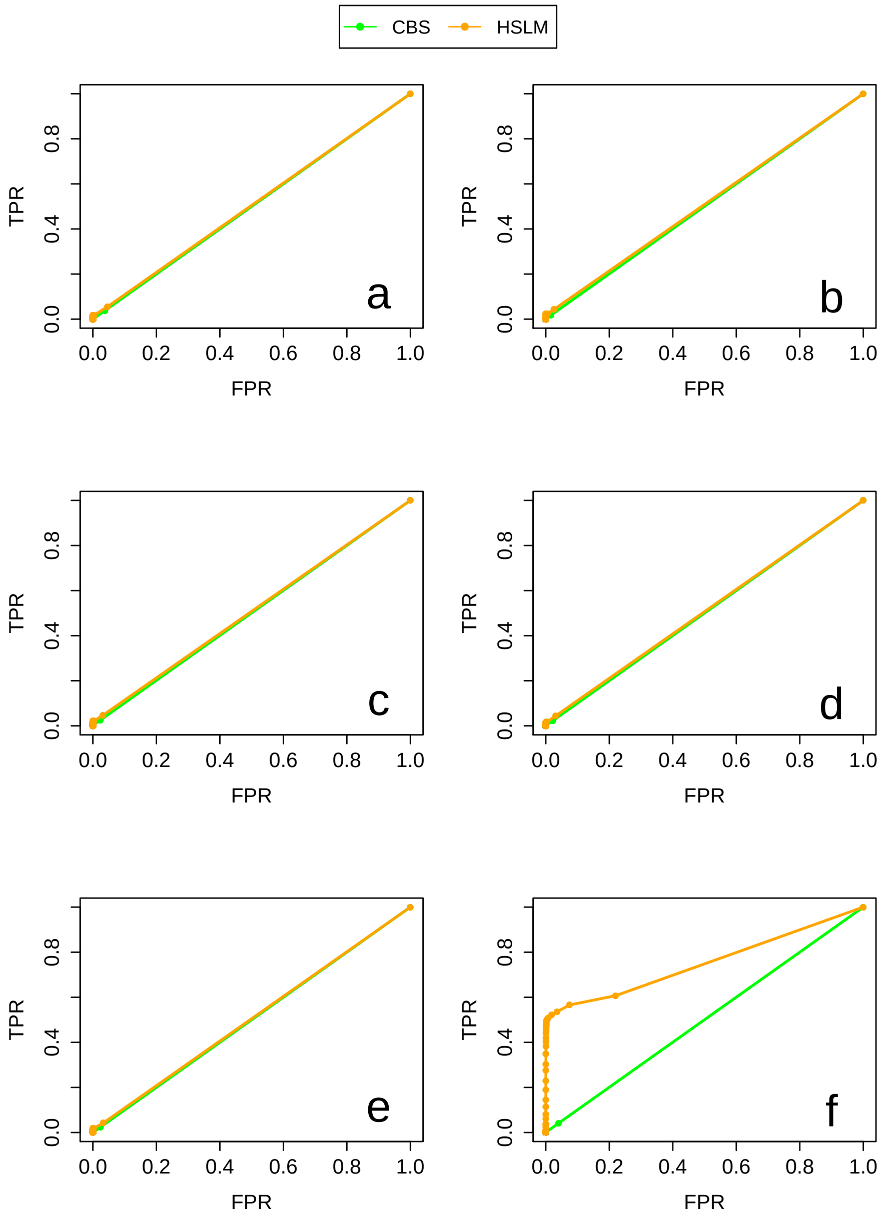

Supplemental Figure 41: Comparison between CBS and HSLM algorithms in the detection of 3-copy alterations made of $N = 10$ exons. The comparison analysis was performed for different values of the distance between adjacent genes ($D = 10Kb$, $D = 50Kb$, $D = 100Kb$, $D = 500Kb$, $D = 1Mb$, $D = 5Mb$). For the HSLM algorith the D_{Norm} was set equal to 10^6. The receiver operating characteristic (ROC) curve was calculated as in Lai *et al.* (2010) and for each of the two algorithms the ROC curve is averaged across 5000 simulations (1000 synthetic chromosome for each g=[1,2,3,4,5]). (a) $D = 10Kb$. (b) $D = 50Kb$. (c) $D = 100Kb$. (d) $D = 500Kb$. (e) $D = 1Mb$. (f) $D = 5Mb$.

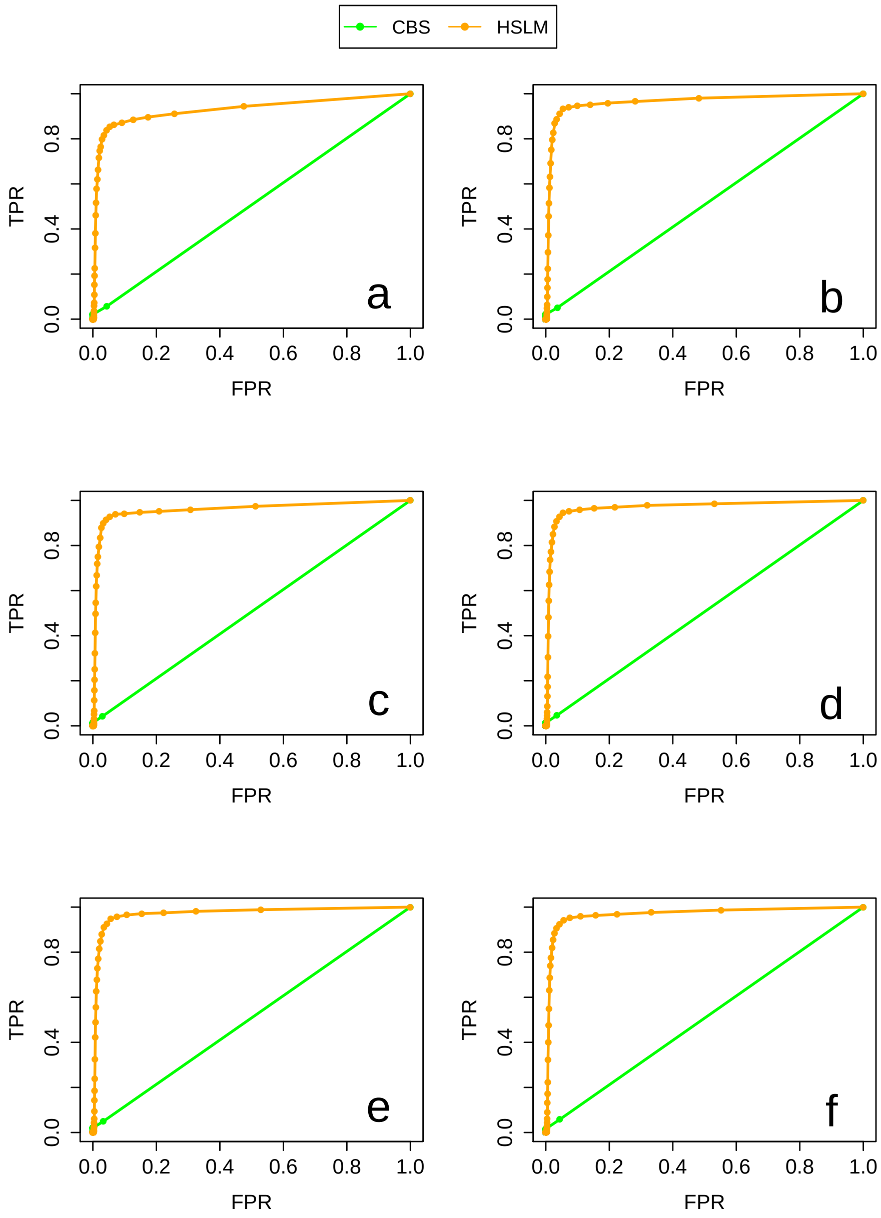

Supplemental Figure 42: Comparison between CBS and HSLM algorithms in the detection of 3-copy alterations made of $N = 20$ exons. The comparison analysis was performed for different values of the distance between adjacent genes ($D = 10Kb$, $D = 50Kb$, $D = 100Kb$, $D = 500Kb$, $D = 1Mb$, $D = 5Mb$). For the HSLM algorith the D_{Norm} was set equal to 10^3. The receiver operating characteristic (ROC) curve was calculated as in Lai *et al.* (2010) and for each of the two algorithms the ROC curve is averaged across 5000 simulations (1000 synthetic chromosome for each g=[1,2,3,4,5]). (a) $D = 10Kb$. (b) $D = 50Kb$. (c) $D = 100Kb$. (d) $D = 500Kb$. (e) $D = 1Mb$. (f) $D = 5Mb$.

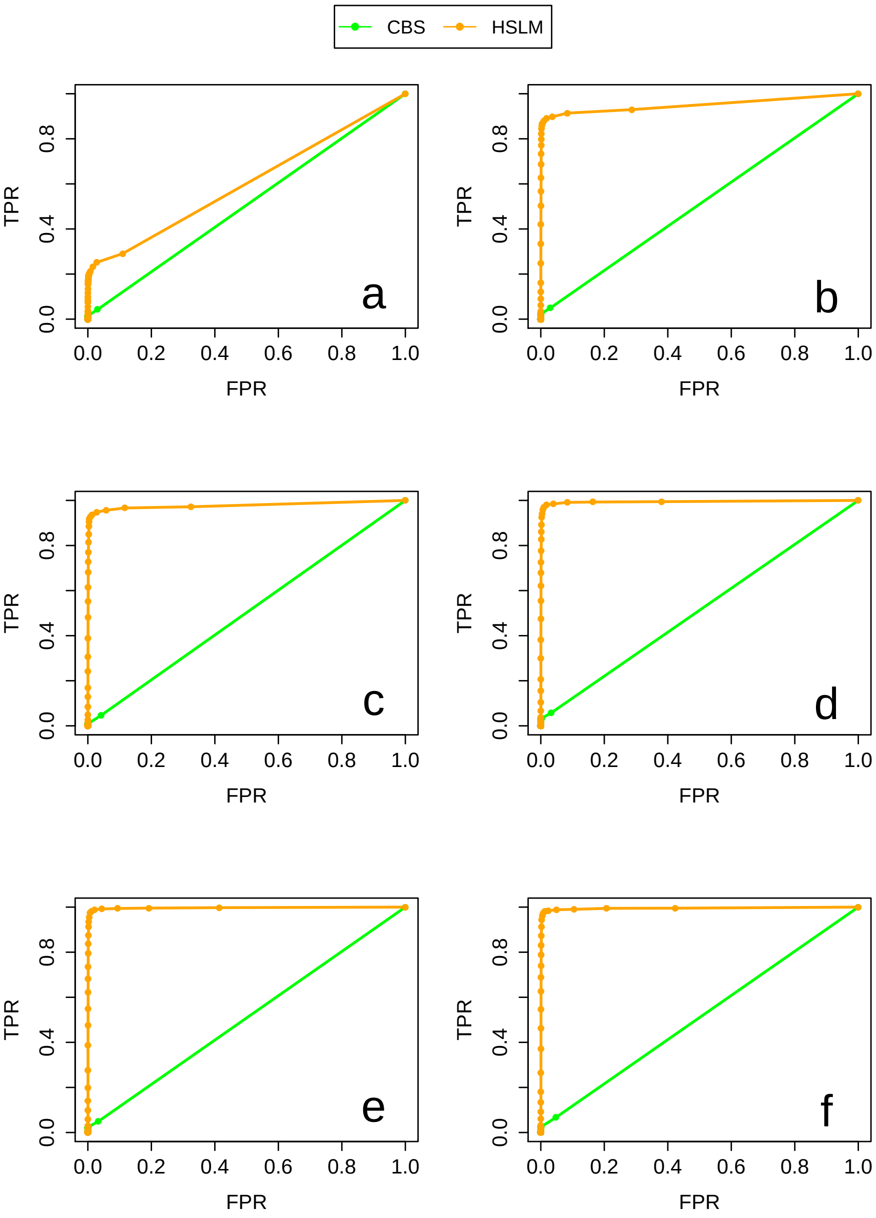

Supplemental Figure 43: Comparison between CBS and HSLM algorithms in the detection of 3-copy alterations made of $N = 20$ exons. The comparison analysis was performed for different values of the distance between adjacent genes ($D = 10Kb$, $D = 50Kb$, $D = 100Kb$, $D = 500Kb$, $D = 1Mb$, $D = 5Mb$). For the HSLM algorith the D_{Norm} was set equal to 10^4. The receiver operating characteristic (ROC) curve was calculated as in Lai *et al.* (2010) and for each of the two algorithms the ROC curve is averaged across 5000 simulations (1000 synthetic chromosome for each g=[1,2,3,4,5]). (a) $D = 10Kb$. (b) $D = 50Kb$. (c) $D = 100Kb$. (d) $D = 500Kb$. (e) $D = 1Mb$. (f) $D = 5Mb$.

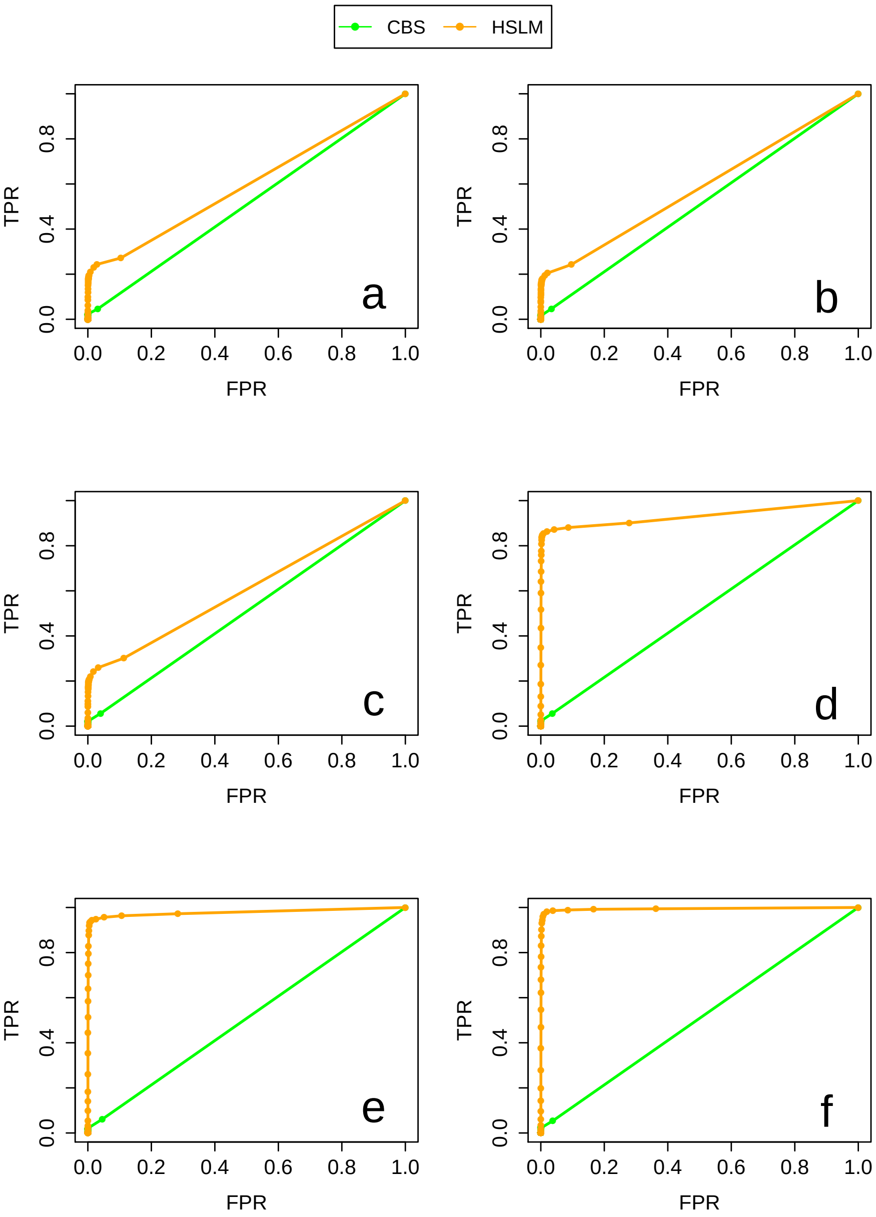

Supplemental Figure 44: Comparison between CBS and HSLM algorithms in the detection of 3-copy alterations made of $N = 20$ exons. The comparison analysis was performed for different values of the distance between adjacent genes ($D = 10Kb$, $D = 50Kb$, $D = 100Kb$, $D = 500Kb$, $D = 1Mb$, $D = 5Mb$). For the HSLM algorith the D_{Norm} was set equal to 10^5. The receiver operating characteristic (ROC) curve was calculated as in Lai *et al.* (2010) and for each of the two algorithms the ROC curve is averaged across 5000 simulations (1000 synthetic chromosome for each g=[1,2,3,4,5]). (a) $D = 10Kb$. (b) $D = 50Kb$. (c) $D = 100Kb$. (d) $D = 500Kb$. (e) $D = 1Mb$. (f) $D = 5Mb$.

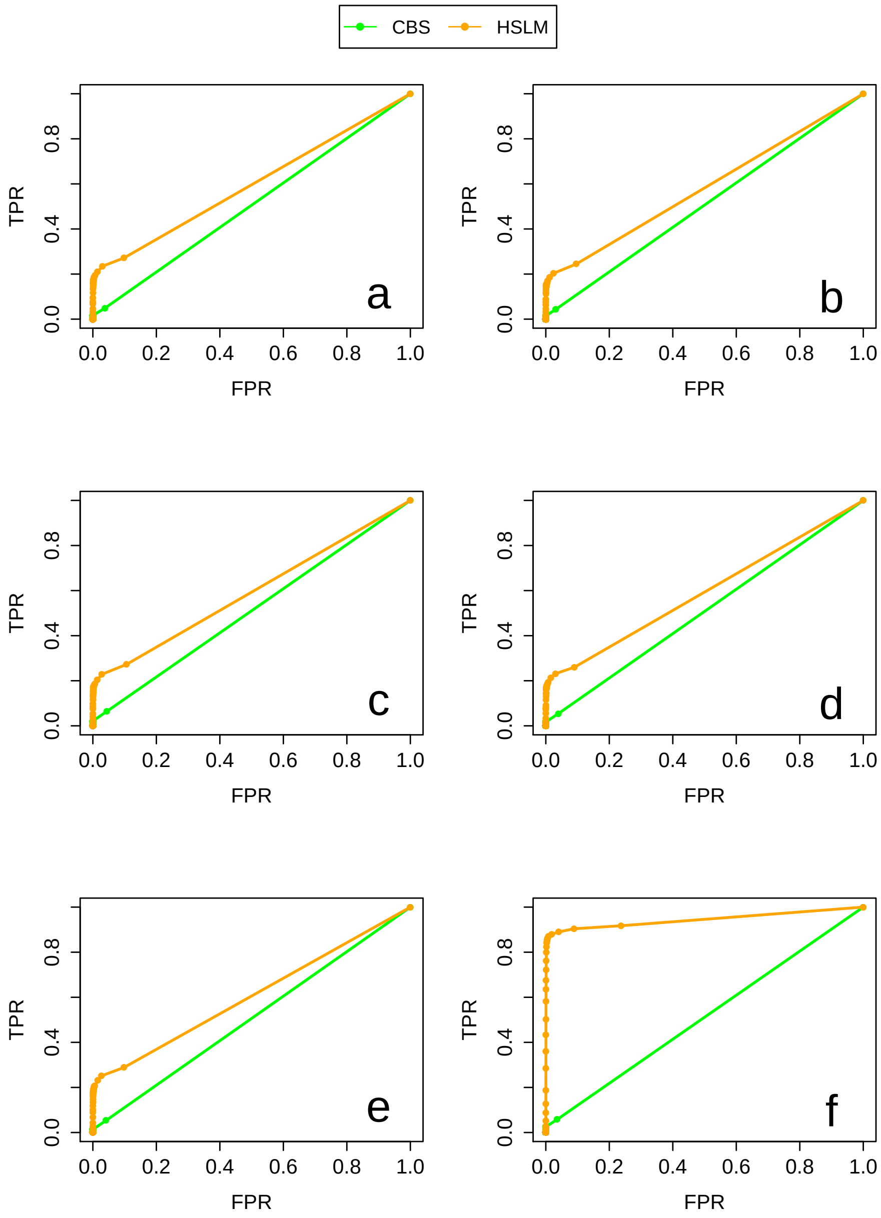

Supplemental Figure 45: Comparison between CBS and HSLM algorithms in the detection of 3-copy alterations made of $N = 20$ exons. The comparison analysis was performed for different values of the distance between adjacent genes ($D = 10Kb$, $D = 50Kb$, $D = 100Kb$, $D = 500Kb$, $D = 1Mb$, $D = 5Mb$). For the HSLM algorith the D_{Norm} was set equal to 10^6. The receiver operating characteristic (ROC) curve was calculated as in Lai *et al.* (2010) and for each of the two algorithms the ROC curve is averaged across 5000 simulations (1000 synthetic chromosome for each g=[1,2,3,4,5]). (a) $D = 10Kb$. (b) $D = 50Kb$. (c) $D = 100Kb$. (d) $D = 500Kb$. (e) $D = 1Mb$. (f) $D = 5Mb$.

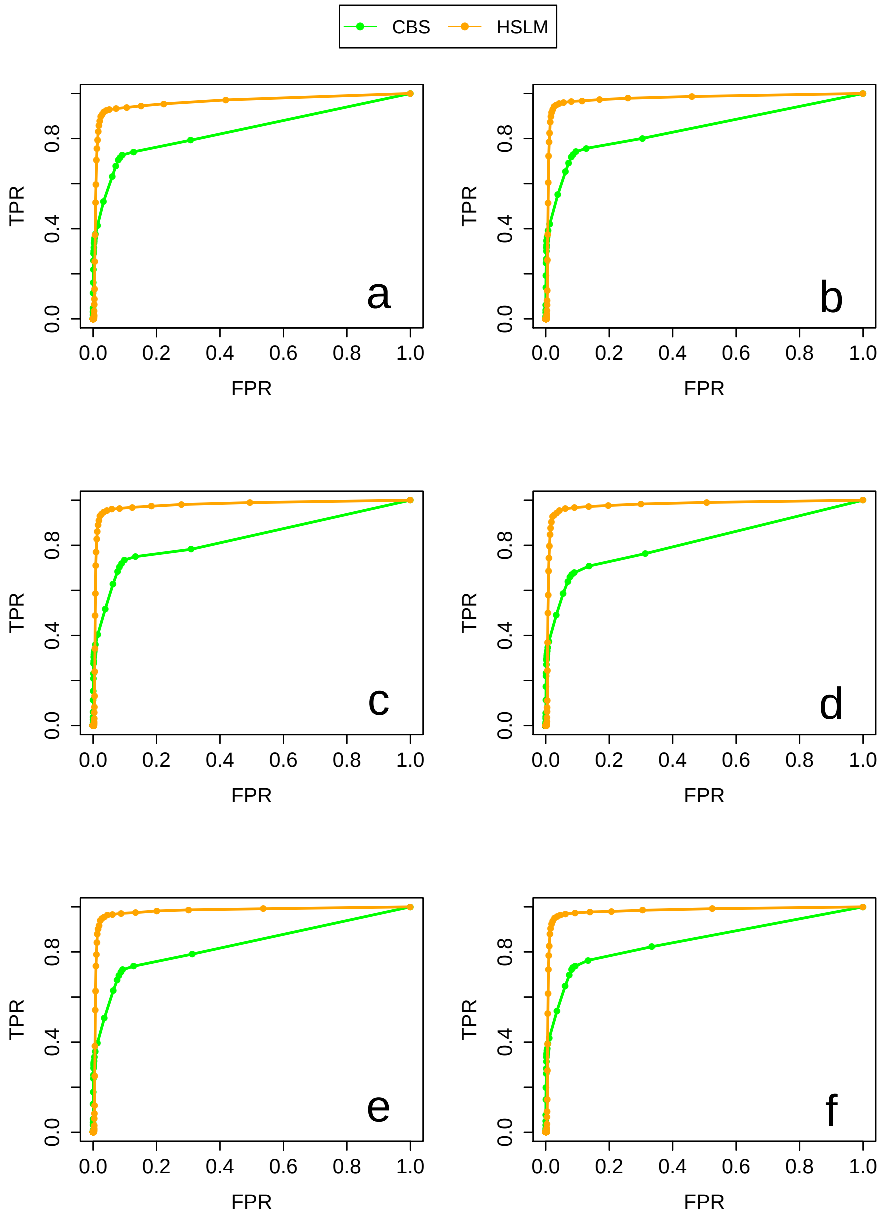

Supplemental Figure 46: Comparison between CBS and HSLM algorithms in the detection of 3-copy alterations made of $N = 50$ exons. The comparison analysis was performed for different values of the distance between adjacent genes ($D = 10Kb$, $D = 50Kb$, $D = 100Kb$, $D = 500Kb$, $D = 1Mb$, $D = 5Mb$). For the HSLM algorith the D_{Norm} was set equal to 10^3. The receiver operating characteristic (ROC) curve was calculated as in Lai *et al.* (2010) and for each of the two algorithms the ROC curve is averaged across 5000 simulations (1000 synthetic chromosome for each g=[1,2,3,4,5]). (a) $D = 10Kb$. (b) $D = 50Kb$. (c) $D = 100Kb$. (d) $D = 500Kb$. (e) $D = 1Mb$. (f) $D = 5Mb$.

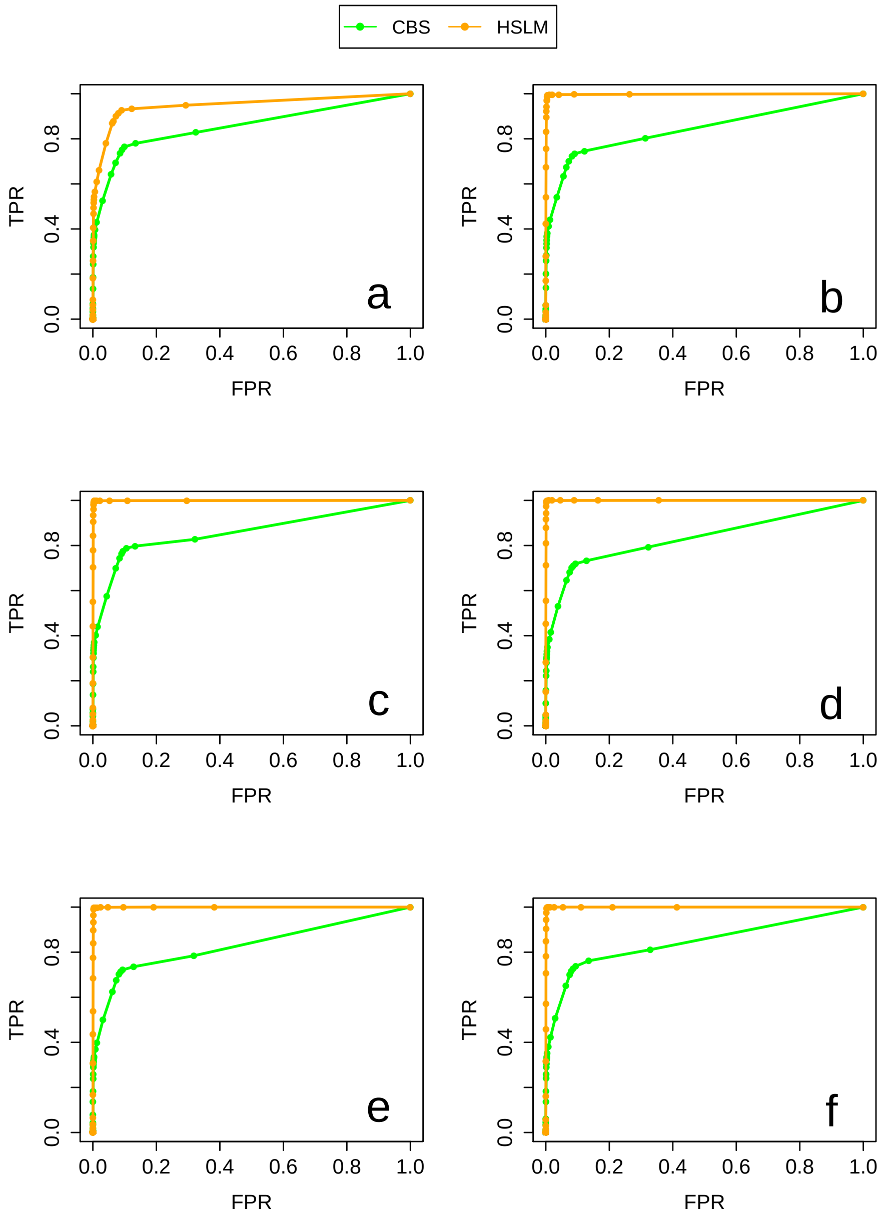

Supplemental Figure 47: Comparison between CBS and HSLM algorithms in the detection of 3-copy alterations made of $N = 50$ exons. The comparison analysis was performed for different values of the distance between adjacent genes ($D = 10Kb$, $D = 50Kb$, $D = 100Kb$, $D = 500Kb$, $D = 1Mb$, $D = 5Mb$). For the HSLM algorith the D_{Norm} was set equal to 10^4. The receiver operating characteristic (ROC) curve was calculated as in Lai *et al.* (2010) and for each of the two algorithms the ROC curve is averaged across 5000 simulations (1000 synthetic chromosome for each g=[1,2,3,4,5]). (a) $D = 10Kb$. (b) $D = 50Kb$. (c) $D = 100Kb$. (d) $D = 500Kb$. (e) $D = 1Mb$. (f) $D = 5Mb$.

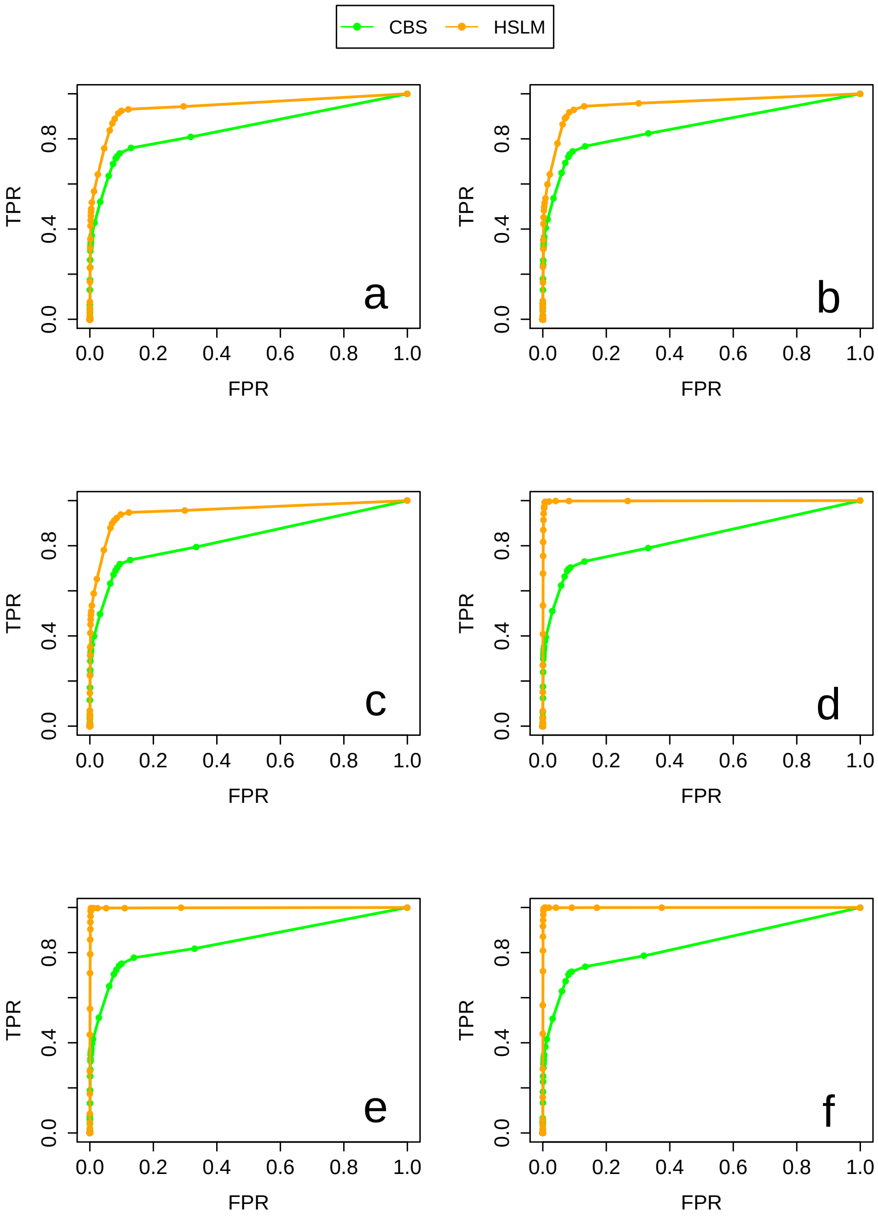

Supplemental Figure 48: Comparison between CBS and HSLM algorithms in the detection of 3-copy alterations made of $N = 50$ exons. The comparison analysis was performed for different values of the distance between adjacent genes ($D = 10Kb$, $D = 50Kb$, $D = 100Kb$, $D = 500Kb$, $D = 1Mb$, $D = 5Mb$). For the HSLM algorith the D_{Norm} was set equal to 10^5. The receiver operating characteristic (ROC) curve was calculated as in Lai *et al.* (2010) and for each of the two algorithms the ROC curve is averaged across 5000 simulations (1000 synthetic chromosome for each g=[1,2,3,4,5]). (a) $D = 10Kb$. (b) $D = 50Kb$. (c) $D = 100Kb$. (d) $D = 500Kb$. (e) $D = 1Mb$. (f) $D = 5Mb$.

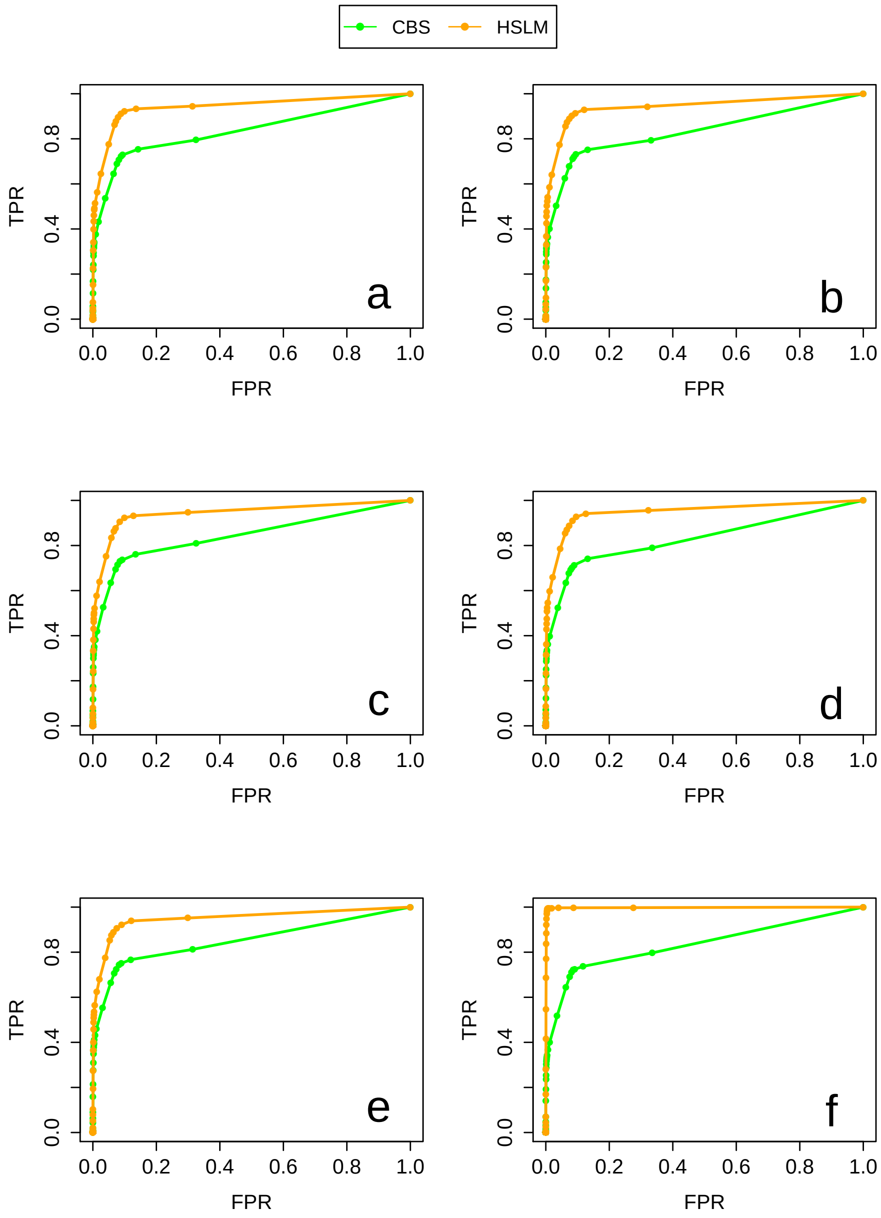

Supplemental Figure 49: Comparison between CBS and HSLM algorithms in the detection of 3-copy alterations made of $N = 50$ exons. The comparison analysis was performed for different values of the distance between adjacent genes ($D = 10Kb$, $D = 50Kb$, $D = 100Kb$, $D = 500Kb$, $D = 1Mb$, $D = 5Mb$). For the HSLM algorith the D_{Norm} was set equal to 10^6. The receiver operating characteristic (ROC) curve was calculated as in Lai *et al.* (2010) and for each of the two algorithms the ROC curve is averaged across 5000 simulations (1000 synthetic chromosome for each g=[1,2,3,4,5]). (a) $D = 10Kb$. (b) $D = 50Kb$. (c) $D = 100Kb$. (d) $D = 500Kb$. (e) $D = 1Mb$. (f) $D = 5Mb$.

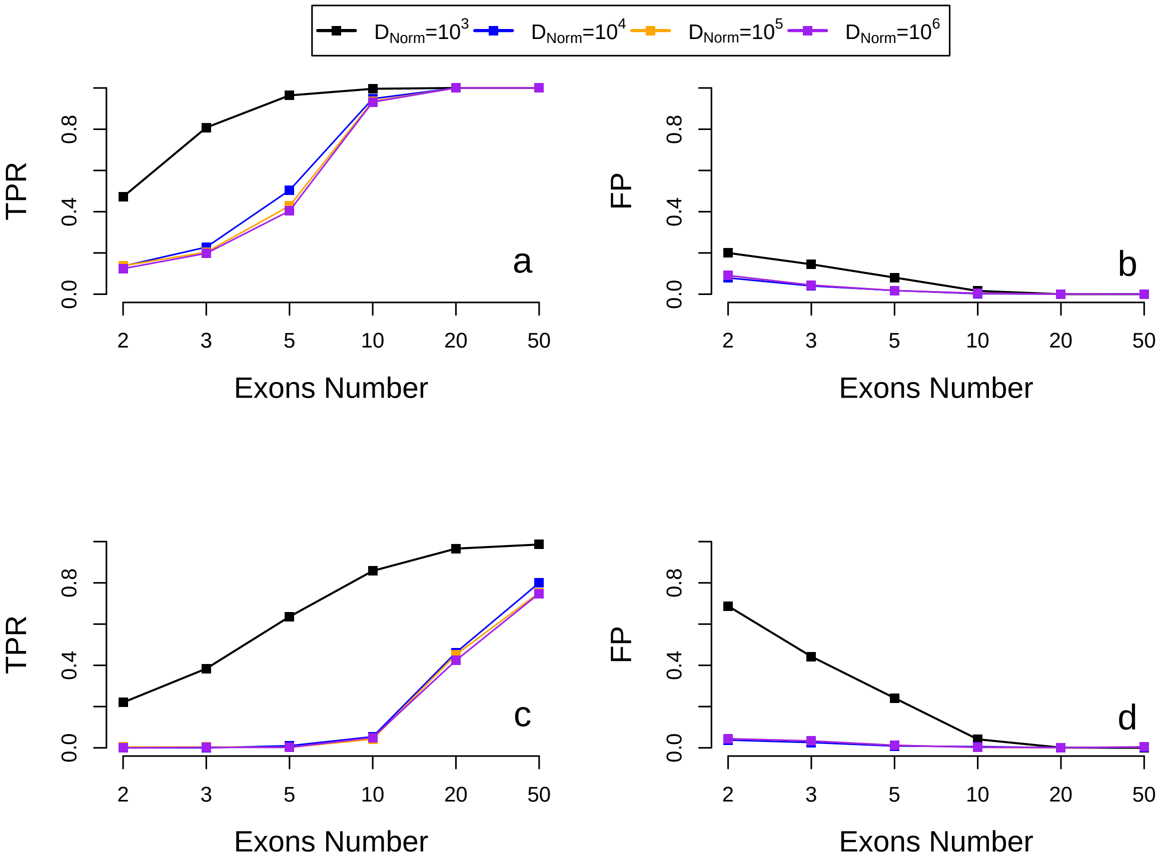

Supplemental Figure 50: Evaluation of the performance of HSLM and FastCallSeq algorithms in the detection of 1-copy and 3-copies alterations in synthetic chromosome with distance between adjacent genes D=10 Kb. The analysis was performed with different values of the D_{Norm} parameter ($D_{Norm} = 10^3$, $D_{Norm} = 10^4$, $D_{Norm} = 10^5$, $D_{Norm} = 10^6$). A detected segment is considered a true positive (TP) if there is at least a 50% overlap between the detected segment and the synthetic altered region, and is considered a false positive (FP) if there is no overlap with a synthetic altered region. In the x axis is reported the number of exons of the altered gene. In the y axis is reported the TPR and the number of false positive (FP) events detected. In panels a and b are reported the TPR and FP for 1-copy regions analysis. In panels c and d are reported the TPR and FP for 3-copies regions analysis. Each point of the curves reported in figure is obtained by averaging TPR and FP across 5000 simulations (1000 synthetic chromosome for each g=[1,2,3,4,5]).

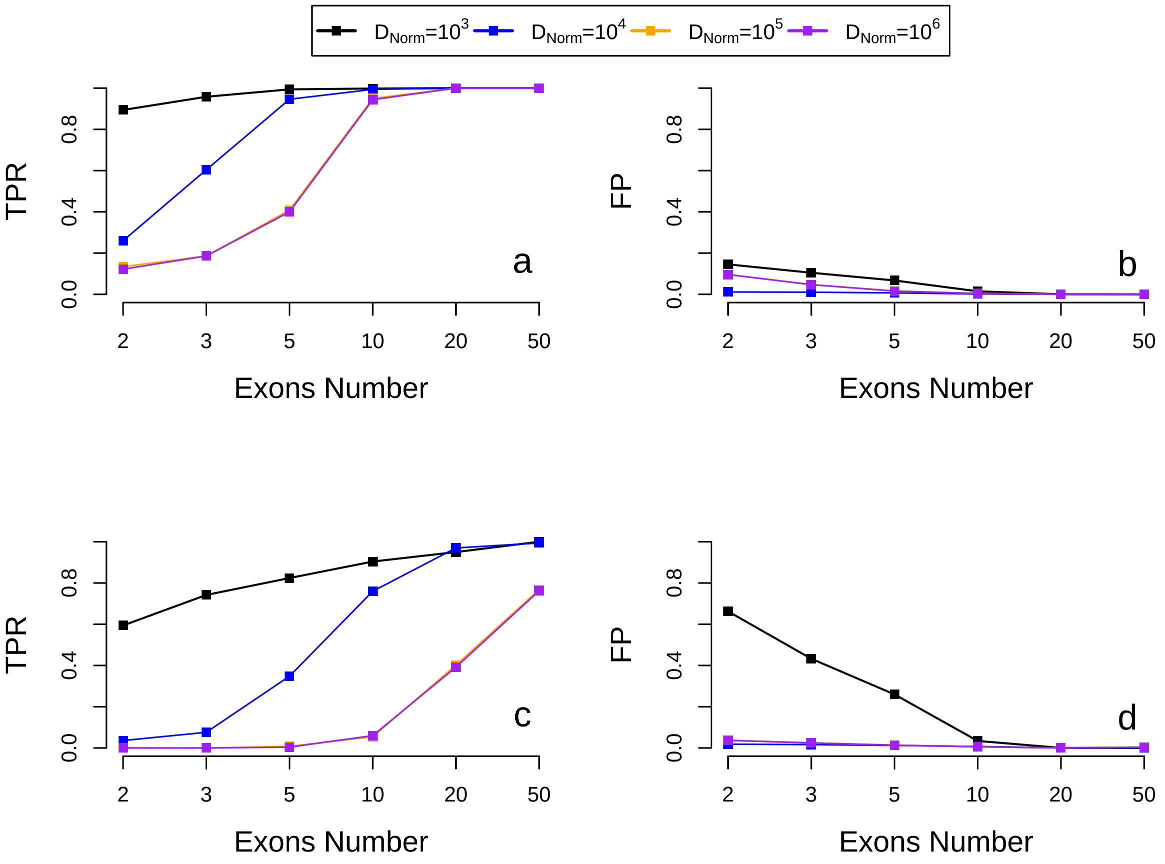

Supplemental Figure 51: Evaluation of the performance of HSLM and FastCallSeq algorithms in the detection of 1-copy and 3-copies alterations in synthetic chromosome with distance between adjacent genes D=50 Kb. The analysis was performed with different values of the D_{Norm} parameter ($D_{Norm} = 10^3$, $D_{Norm} = 10^4$, $D_{Norm} = 10^5$, $D_{Norm} = 10^6$). A detected segment is considered a true positive (TP) if there is at least a 50% overlap between the detected segment and the synthetic altered region, and is considered a false positive (FP) if there is no overlap with a synthetic altered region. In the x axis is reported the number of exons of the altered gene. In the y axis is reported the TPR and the number of false positive (FP) events detected. In panels a and b are reported the TPR and FP for 1-copy regions analysis. In panels c and d are reported the TPR and FP for 3-copies regions analysis. Each point of the curves reported in figure is obtained by averaging TPR and FP across 5000 simulations (1000 synthetic chromosome for each g=[1,2,3,4,5]).

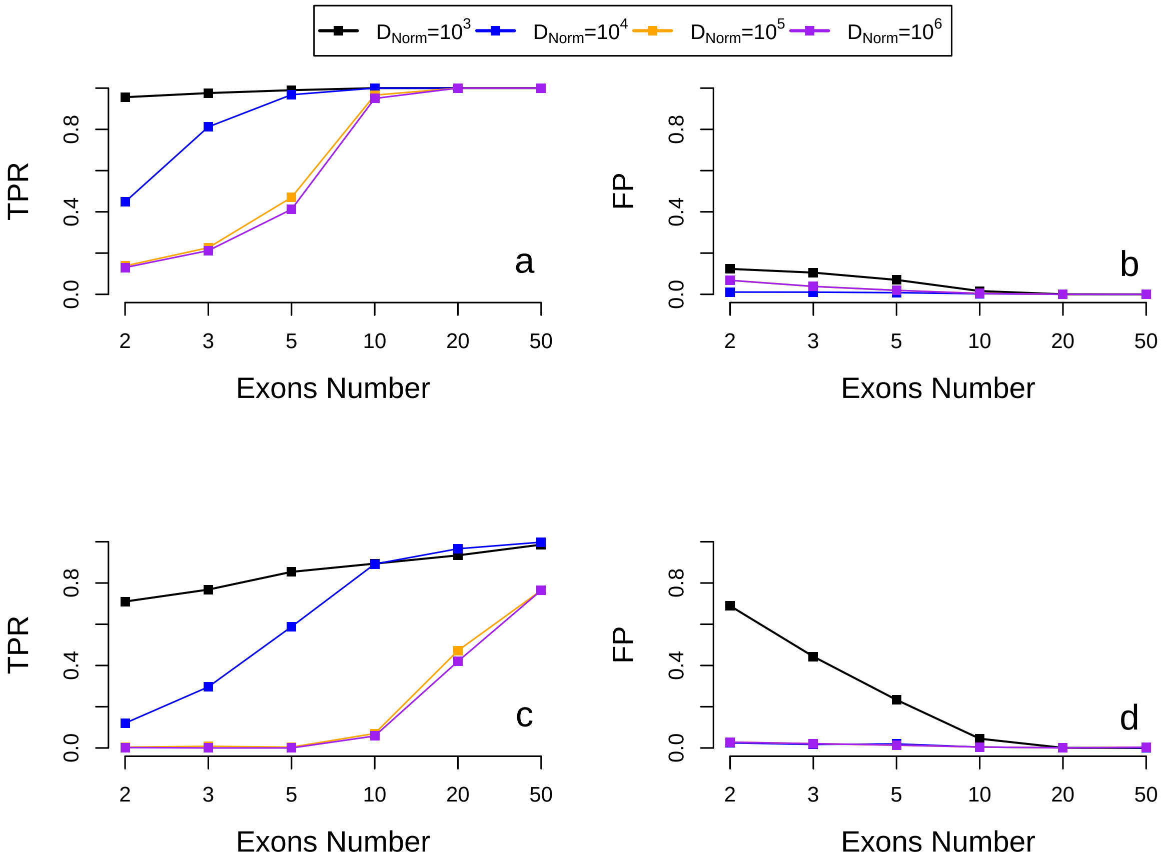

Supplemental Figure 52: Evaluation of the performance of HSLM and FastCallSeq algorithms in the detection of 1-copy and 3-copies alterations in synthetic chromosome with distance between adjacent genes D=100 Kb. The analysis was performed with different values of the D_{Norm} parameter ($D_{Norm} = 10^3$, $D_{Norm} = 10^4$, $D_{Norm} = 10^5$, $D_{Norm} = 10^6$). A detected segment is considered a true positive (TP) if there is at least a 50% overlap between the detected segment and the synthetic altered region, and is considered a false positive (FP) if there is no overlap with a synthetic altered region. In the x axis is reported the number of exons of the altered gene. In the y axis is reported the TPR and the number of false positive (FP) events detected. In panels a and b are reported the TPR and FP for 1-copy regions analysis. In panels c and d are reported the TPR and FP for 3-copies regions analysis. Each point of the curves reported in figure is obtained by averaging TPR and FP across 5000 simulations (1000 synthetic chromosome for each g=[1,2,3,4,5]).

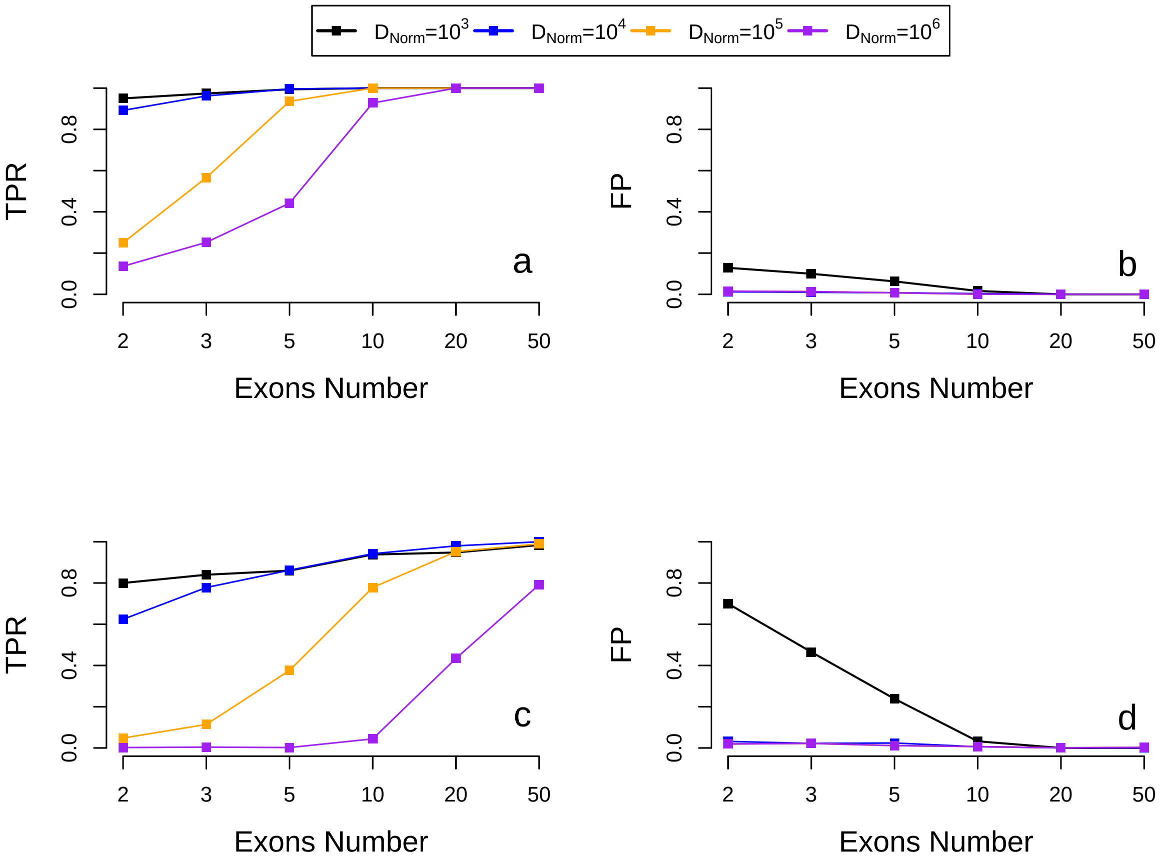

Supplemental Figure 53: Evaluation of the performance of HSLM and FastCallSeq algorithms in the detection of 1-copy and 3-copies alterations in synthetic chromosome with distance between adjacent genes D=500 Kb. The analysis was performed with different values of the D_{Norm} parameter ($D_{Norm} = 10^3$, $D_{Norm} = 10^4$, $D_{Norm} = 10^5$, $D_{Norm} = 10^6$). A detected segment is considered a true positive (TP) if there is at least a 50% overlap between the detected segment and the synthetic altered region, and is considered a false positive (FP) if there is no overlap with a synthetic altered region. In the x axis is reported the number of exons of the altered gene. In the y axis is reported the TPR and the number of false positive (FP) events detected. In panels a and b are reported the TPR and FP for 1-copy regions analysis. In panels c and d are reported the TPR and FP for 3-copies regions analysis. Each point of the curves reported in figure is obtained by averaging TPR and FP across 5000 simulations (1000 synthetic chromosome for each g=[1,2,3,4,5]).

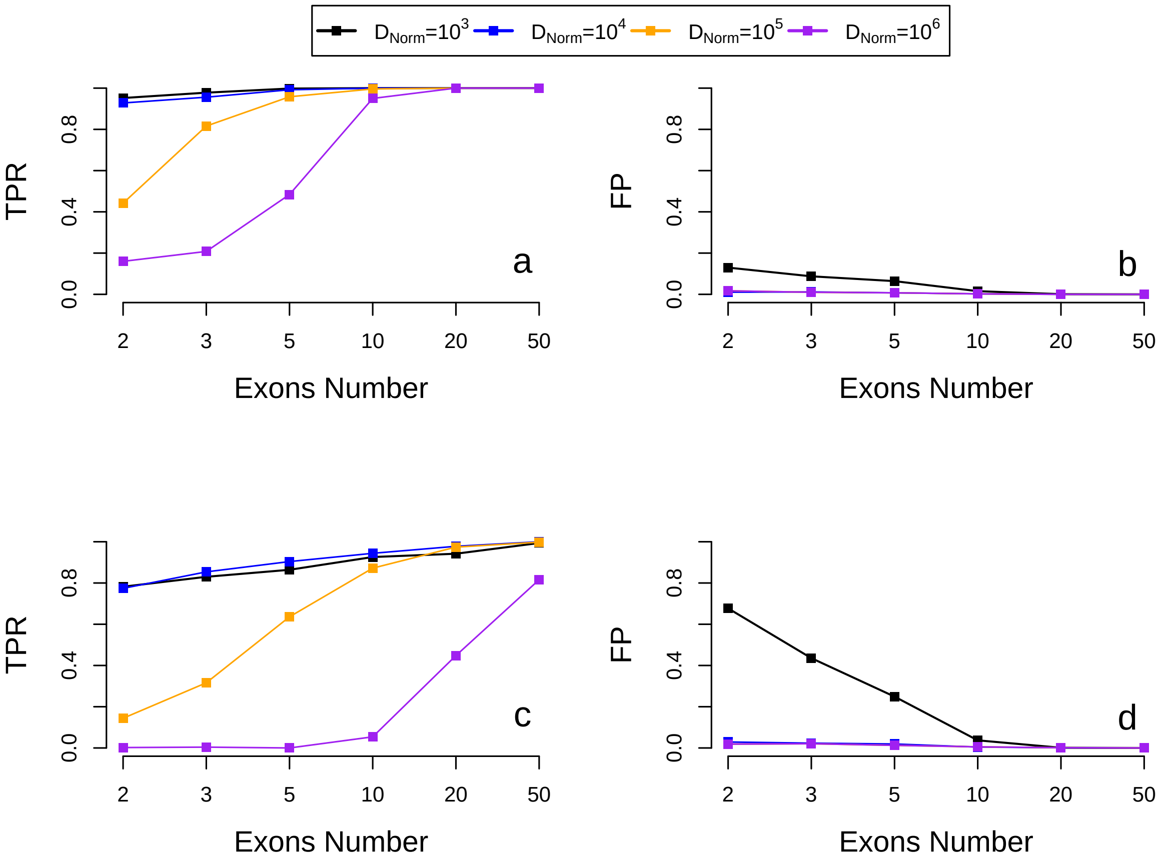

Supplemental Figure 54: Evaluation of the performance of HSLM and FastCallSeq algorithms in the detection of 1-copy and 3-copies alterations in synthetic chromosome with distance between adjacent genes D=1 Mb. The analysis was performed with different values of the D_{Norm} parameter ($D_{Norm} = 10^3$, $D_{Norm} = 10^4$, $D_{Norm} = 10^5$, $D_{Norm} = 10^6$). A detected segment is considered a true positive (TP) if there is at least a 50% overlap between the detected segment and the synthetic altered region, and is considered a false positive (FP) if there is no overlap with a synthetic altered region. In the x axis is reported the number of exons of the altered gene. In the y axis is reported the TPR and the number of false positive (FP) events detected. In panels a and b are reported the TPR and FP for 1-copy regions analysis. In panels c and d are reported the TPR and FP for 3-copies regions analysis. Each point of the curves reported in figure is obtained by averaging TPR and FP across 5000 simulations (1000 synthetic chromosome for each g=[1,2,3,4,5]).

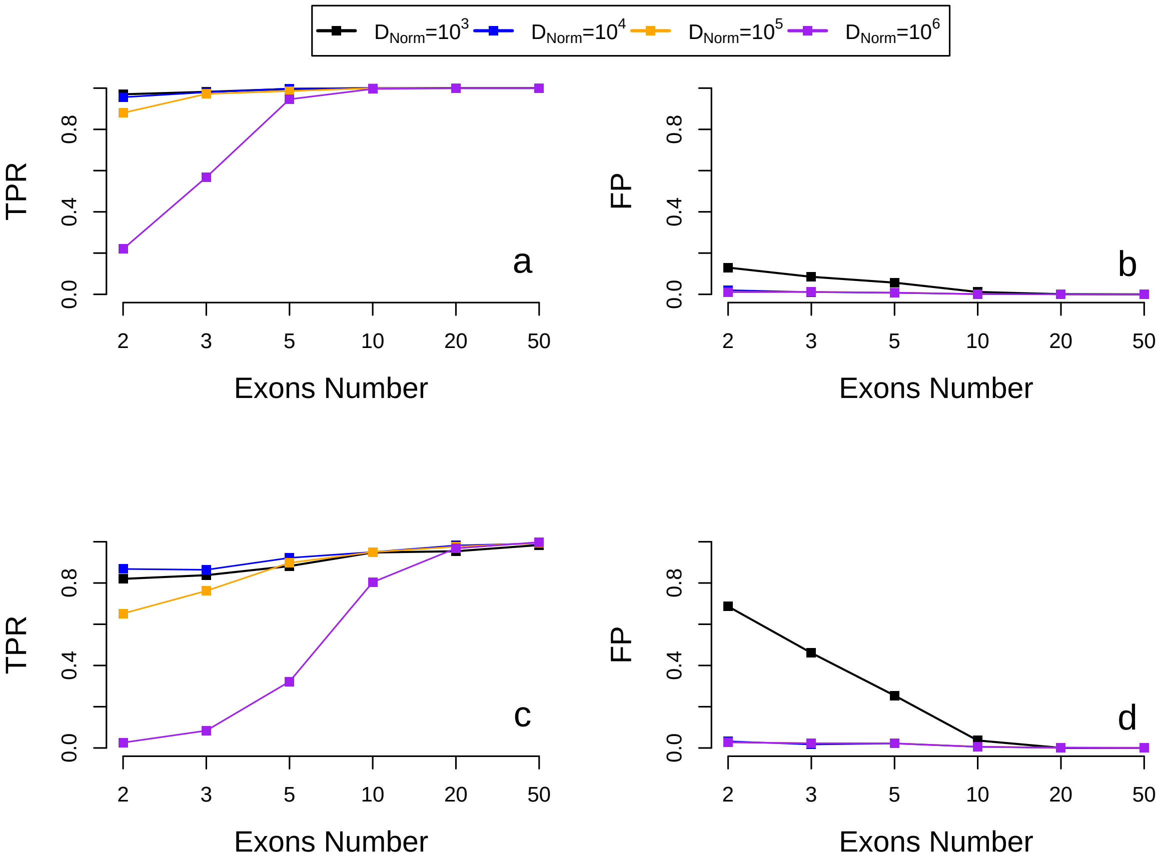

Supplemental Figure 55: Evaluation of the performance of HSLM and FastCallSeq algorithms in the detection of 1-copy and 3-copies alterations in synthetic chromosome with distance between adjacent genes D=5 Mb. The analysis was performed with different values of the D_{Norm} parameter ($D_{Norm} = 10^3$, $D_{Norm} = 10^4$, $D_{Norm} = 10^5$, $D_{Norm} = 10^6$). A detected segment is considered a true positive (TP) if there is at least a 50% overlap between the detected segment and the synthetic altered region, and is considered a false positive (FP) if there is no overlap with a synthetic altered region. In the x axis is reported the number of exons of the altered gene. In the y axis is reported the TPR and the number of false positive (FP) events detected. In panels a and b are reported the TPR and FP for 1-copy regions analysis. In panels c and d are reported the TPR and FP for 3-copies regions analysis. Each point of the curves reported in figure is obtained by averaging TPR and FP across 5000 simulations (1000 synthetic chromosome for each g=[1,2,3,4,5]).

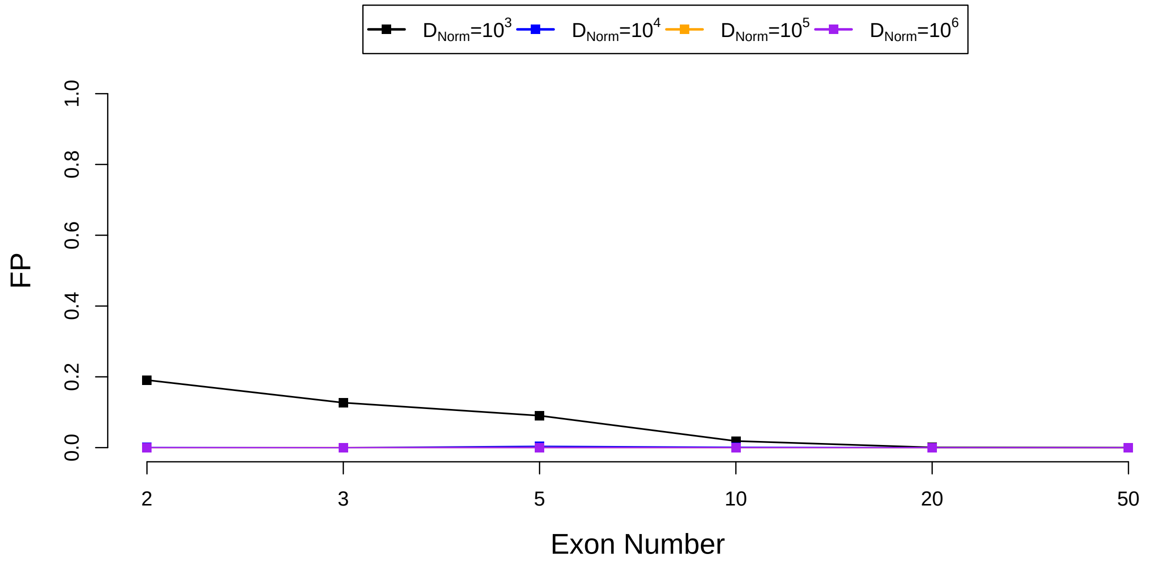

Supplemental Figure 56: Evaluation of the performance of HSLM and FastCallSeq algorithms in the detection of false positive events in synthetic chromosome with no altered regions (g=0). The analysis was performed with different values of the D_{Norm} parameter ($D_{Norm} = 10^3$, $D_{Norm} = 10^4$, $D_{Norm} = 10^5$, $D_{Norm} = 10^6$). Each point of the curves reported in figure is obtained by averaging the number of FP events across 1000 simulations.

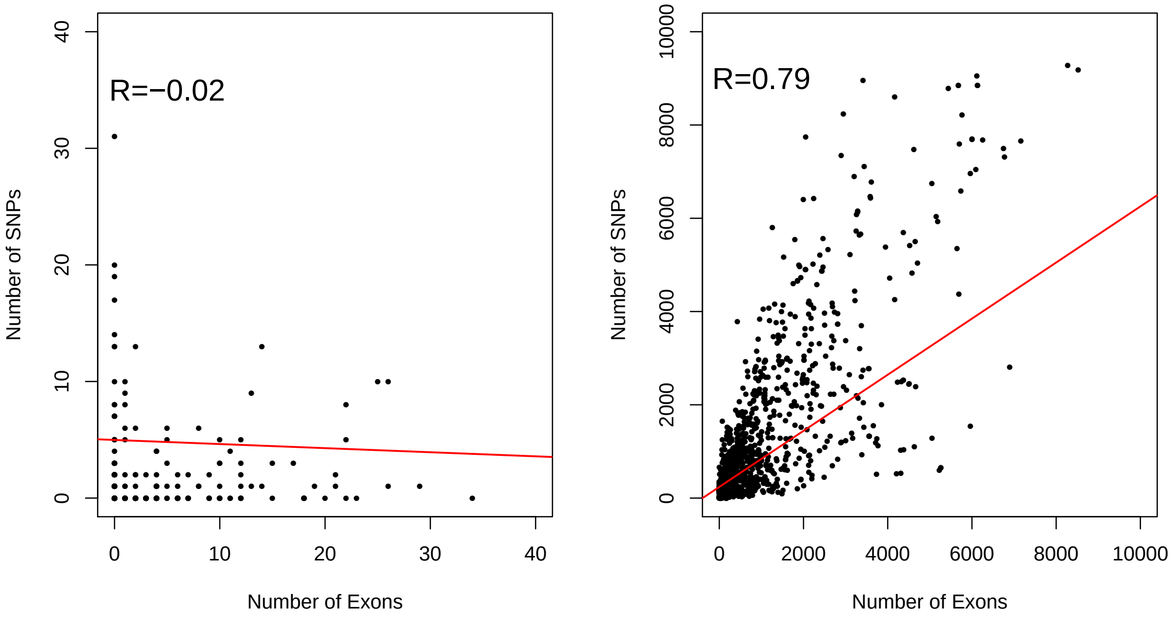

Supplemental Figure 57: Comparison between the number of Affymetrix SNP probes and the number of exons that cover each segmented region. On the left panel, segmented regions smaller than 100 Kb do not have a comparable number of SNP probes and exons (R=-0.02). Differently, on the right panel, segmented regions larger than 1 Mb have a comparable number of SNP probes and exons (R=0.8).

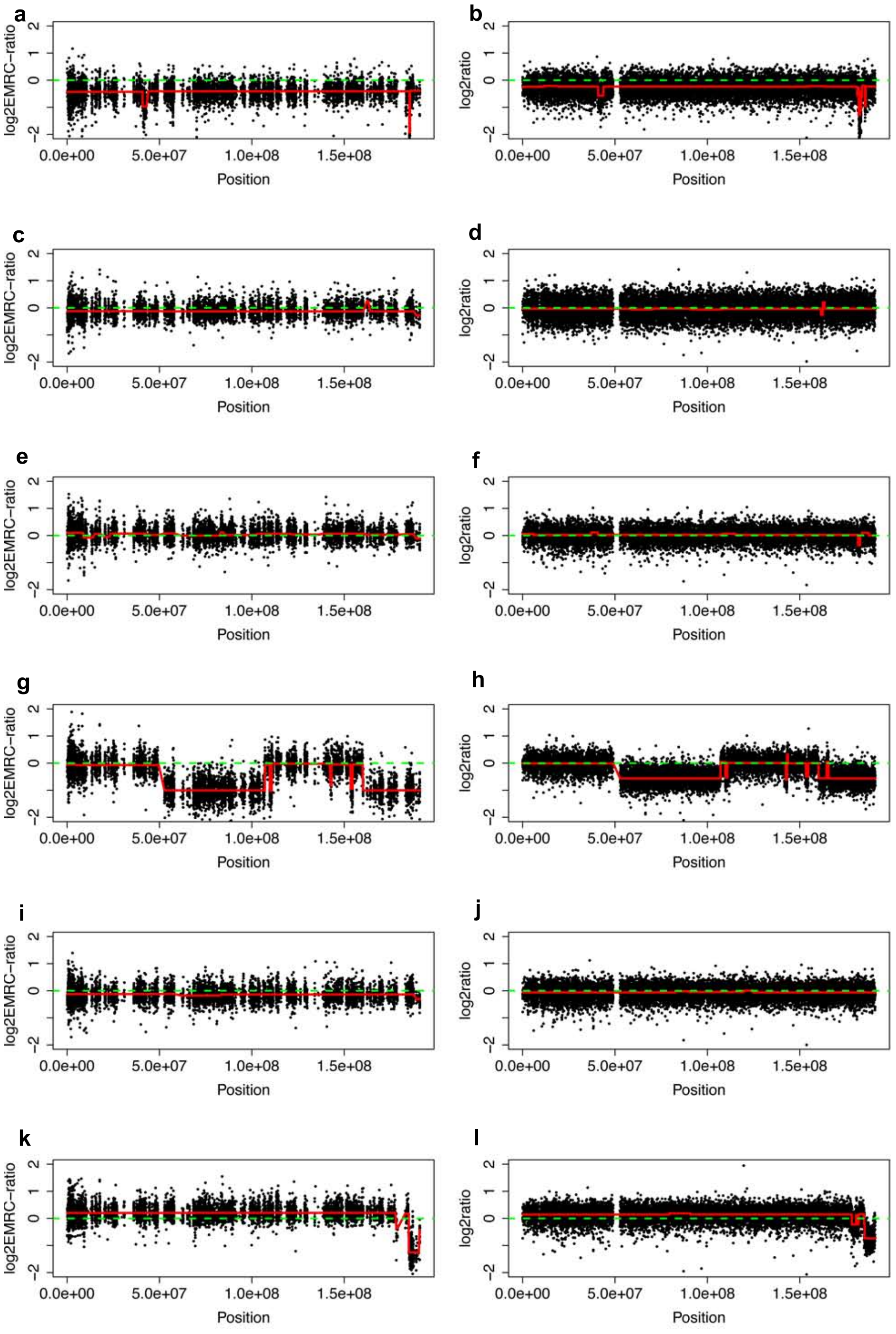

Supplemental Figure 58: Comparison of the segmentation results on WES data (left) and SNP array data (right) for the six melanoma samples on chromosome 4. Samples are vertically ordered: Me01 (a, b), Me02 (c, d), Me04 (e, f), Me05 (g, h), Me08 (i, j), Me12 (k, l). Continuos red lines represent segmentation results generated by applying HSLM (on WES data) or SLM (on SNP array data) algorithms. Dashed green lines indicate the diploid baseline.

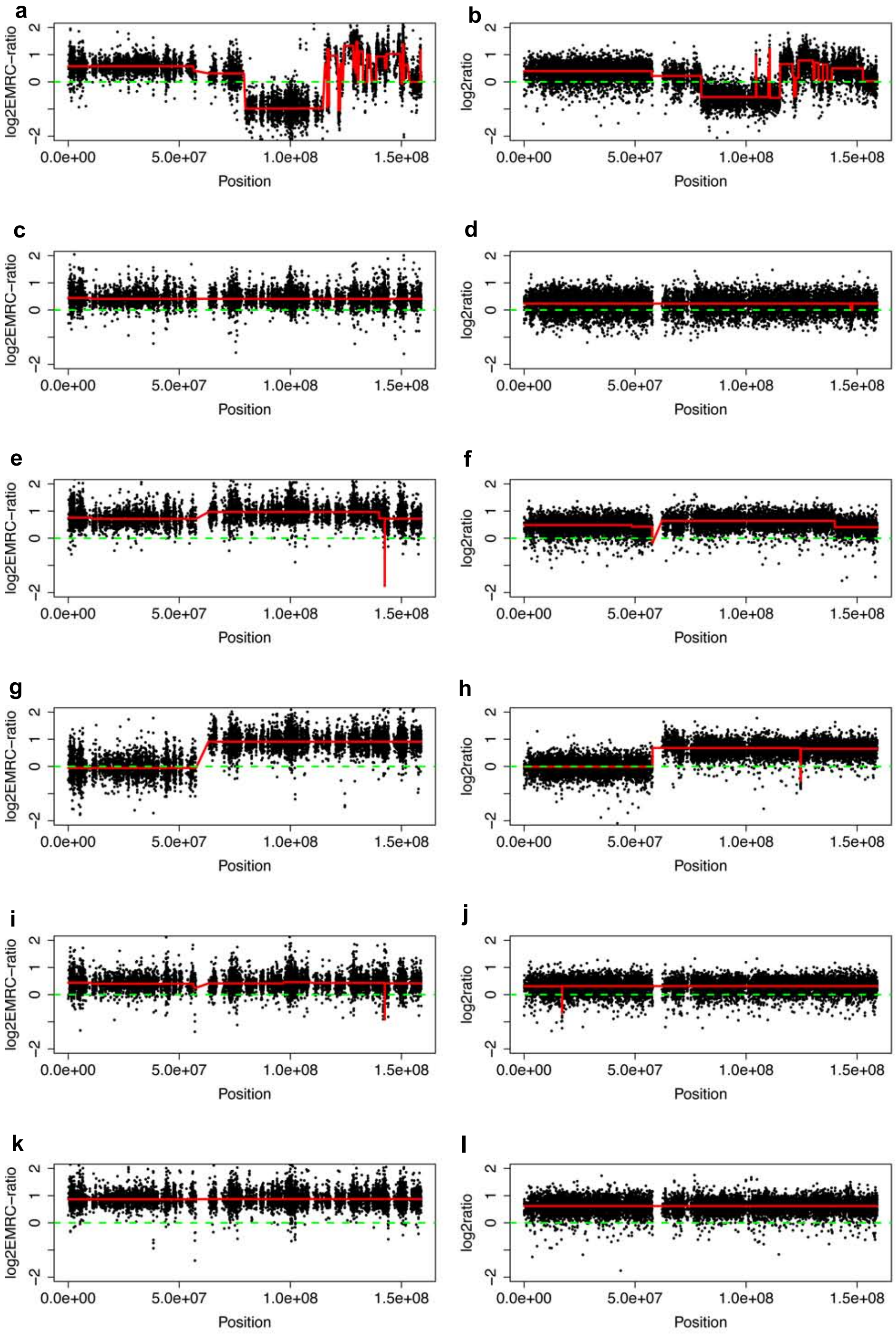

Supplemental Figure 59: Comparison of the segmentation results on WES data (left) and SNP array data (right) for the six melanoma samples on chromosome 7. Samples are vertically ordered: Me01 (a, b), Me02 (c, d), Me04 (e, f), Me05 (g, h), Me08 (i, j), Me12 (k, l). Continuos red lines represent segmentation results generated by applying HSLM (on WES data) or SLM (on SNP array data) algorithms. Dashed green lines indicate the diploid baseline.

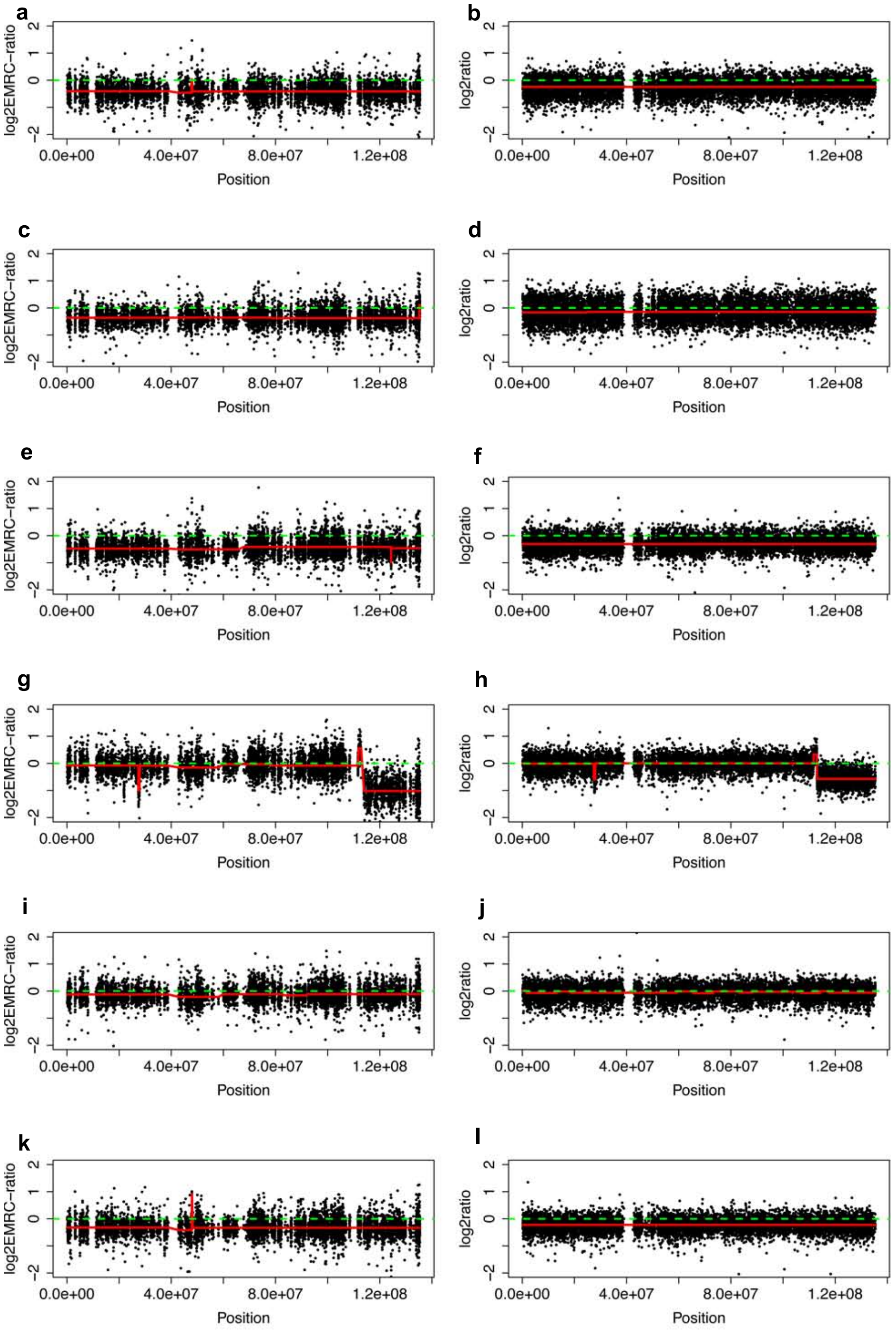

Supplemental Figure 60: Comparison of the segmentation results on WES data (left) and SNP array data (right) for the six melanoma samples on chromosome 10. Samples are vertically ordered: Me01 (a, b), Me02 (c, d), Me04 (e, f), Me05 (g, h), Me08 (i, j), Me12 (k, l). Continuos red lines represent segmentation results generated by applying HSLM (on WES data) or SLM (on SNP array data) algorithms. Dashed green lines indicate the diploid baseline.

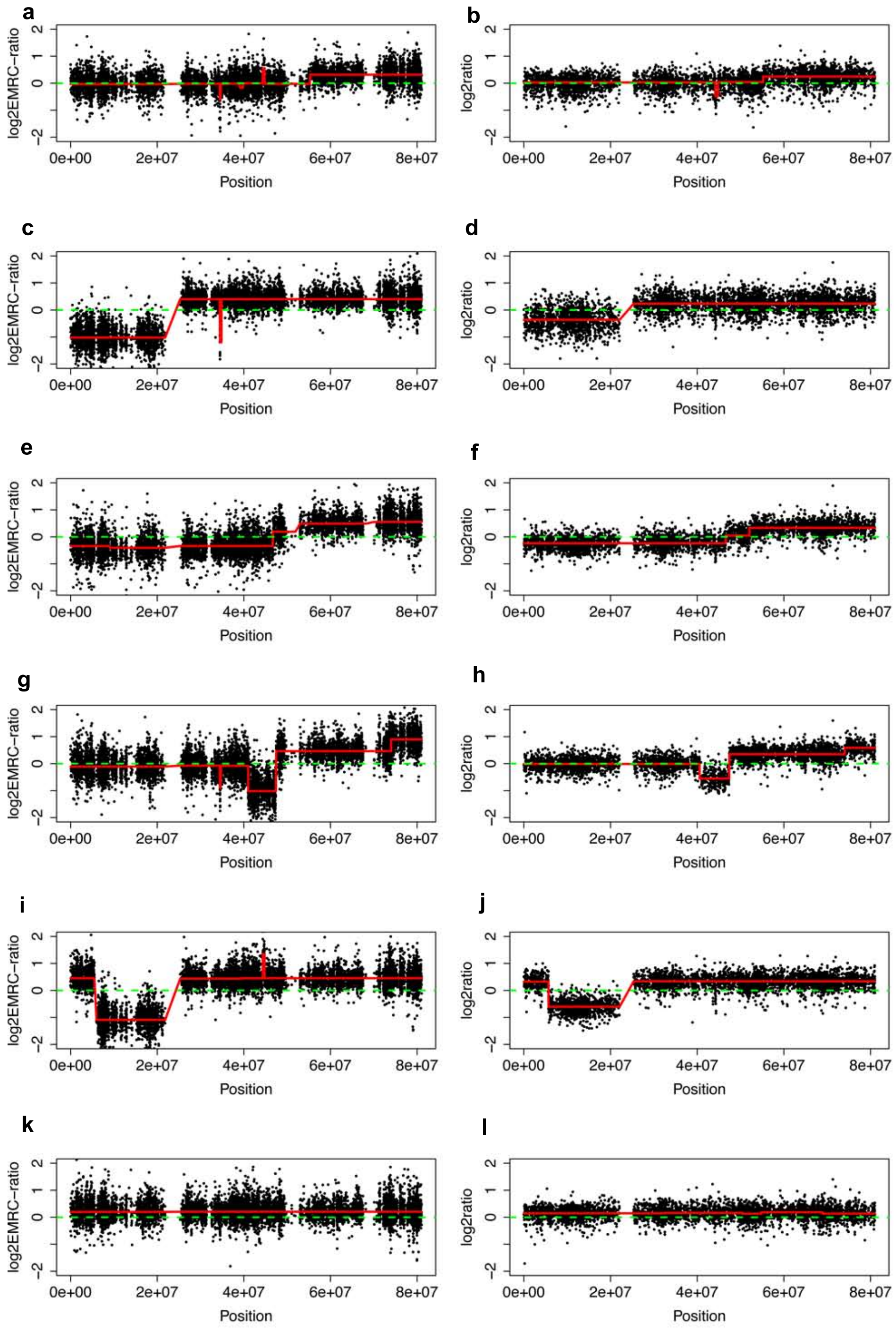

Supplemental Figure 61: Comparison of the segmentation results on WES data (left) and SNP array data (right) for the six melanoma samples on chromosome 17. Samples are vertically ordered: Me01 (a, b), Me02 (c, d), Me04 (e, f), Me05 (g, h), Me08 (i, j), Me12 (k, l). Continuos red lines represent segmentation results generated by applying HSLM (on WES data) or SLM (on SNP array data) algorithms. Dashed green lines indicate the diploid baseline.

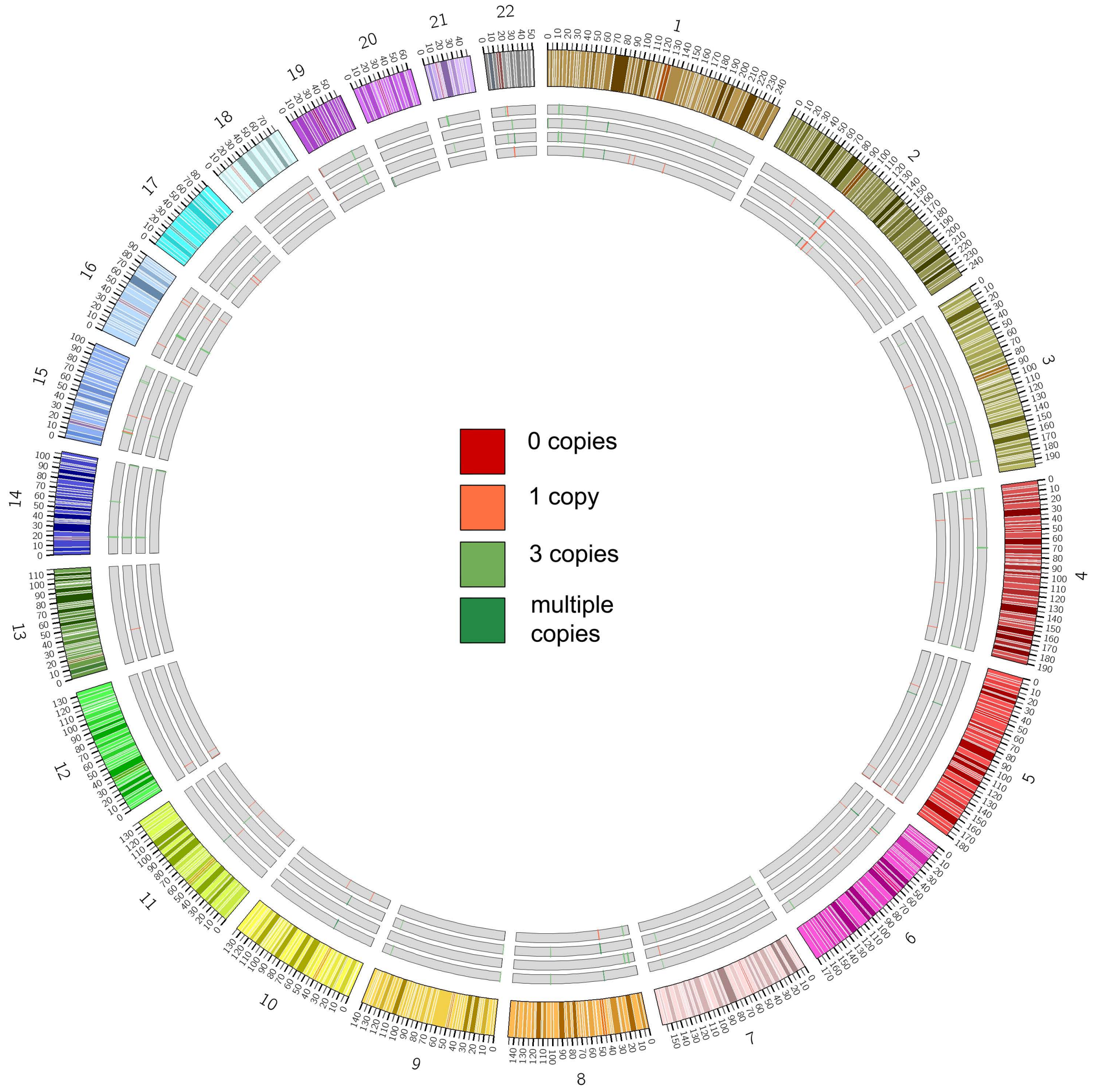

Supplemental Figure 62: Summary of the results obtained by EXCAVATOR in the analysis on the intellectual disability dataset. The Circos plot summarizes all the CNV regions detected in each of the two siblings by both exome-seq and SNP array analysis. On each chromosome, ID1 and ID2 samples are represented with two tracks (WES and SNP array, respectively) for each. CNV regions are distinguished by color-code into two-copy deletion (red), one-copy deletion (orange), one-copy amplification (light green) and multiple-copy amplification (dark green).

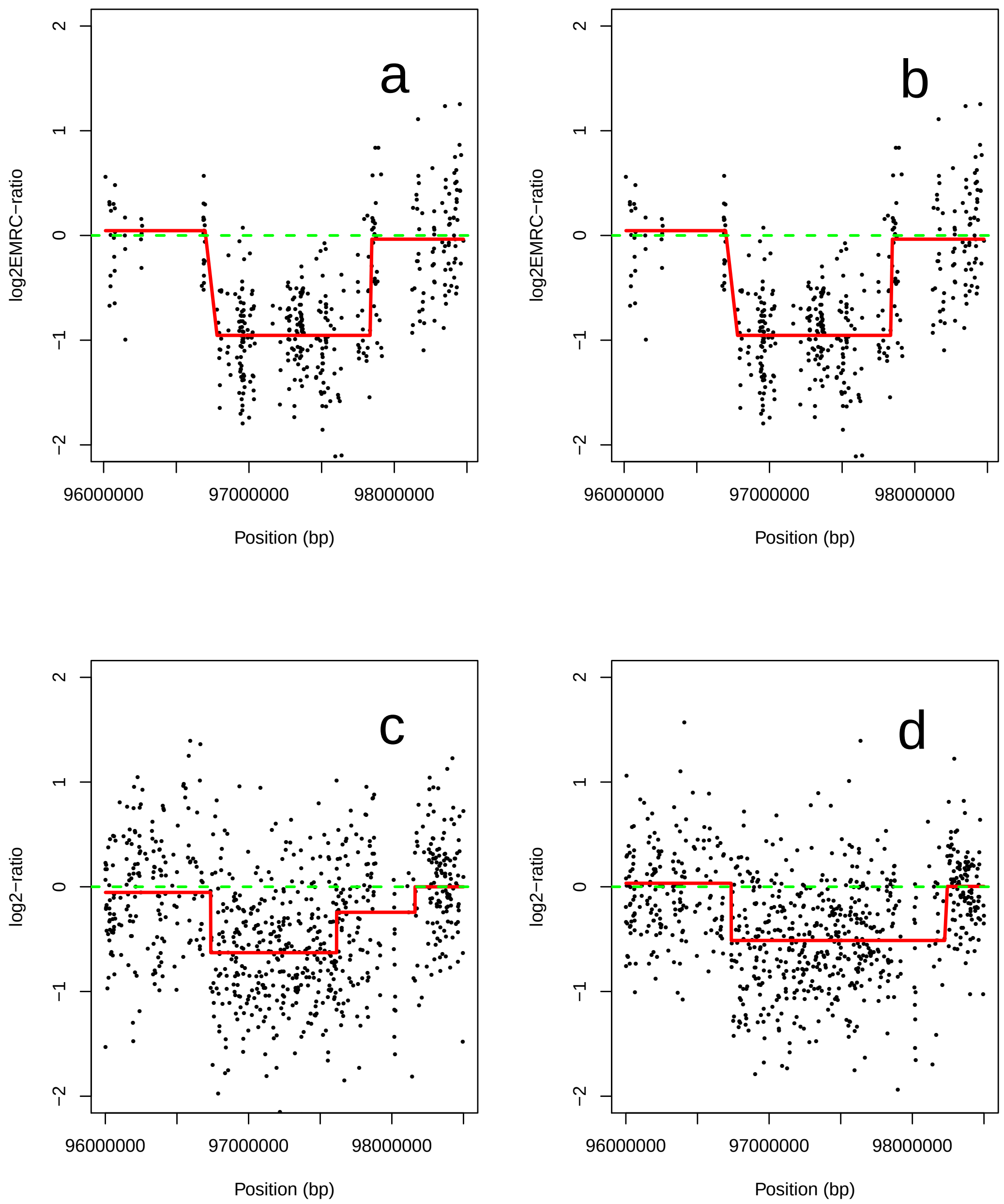

Supplemental Figure 63: Comparison between WES (panels a and b) and SNP array (panels c and d) data on chromosome 2 of the two siblings (panels a and c for ID1 and panels b and d for ID2) affected by intellectual disability. Continuos red lines represent segmentation results generated by applying HSLM (on WES data) or SLM (on SNP array data) algorithms. Dashed green lines indicate diploid baseline.

	BWA 100	BWA 75	BWA 50	Bowtie2 100	Bowtie2 75	Bowtie 50	SOAP2 100	SOAP2 75	SOAP2 50
BWA 100	1	0.9996	0.9988	0.9996	0.9995	0.9988	0.9873	0.9947	0.9971
BWA 75	0.9996	1	0.9996	0.9995	0.9999	0.9996	0.9848	0.9936	0.9971
BWA 50	0.9988	0.9996	1	0.9988	0.9996	0.9999	0.983	0.9925	0.997
Bowtie2 100	0.9996	0.9995	0.9988	1	0.9997	0.9988	0.9846	0.9926	0.9958
Bowtie2 75	0.9995	0.9999	0.9996	0.9997	1	0.9996	0.9841	0.9931	0.9968
Bowtie 50	0.9988	0.9996	0.9999	0.9988	0.9996	1	0.9838	0.9933	0.9976
SOAP2 100	0.9873	0.9848	0.983	0.9846	0.9841	0.9838	1	0.9963	0.9903
SOAP2 75	0.9947	0.9936	0.9925	0.9926	0.9931	0.9933	0.9963	1	0.9982
SOAP2 50	0.9971	0.9971	0.997	0.9958	0.9968	0.9976	0.9903	0.9982	1

Supplemental Figure 64: Colorplot of the correlation coefficient between raw read count for each combination of aligner/read length. Each square of the colorplot represent the correlation coefficient between the raw read count of two different alignment and different read length.

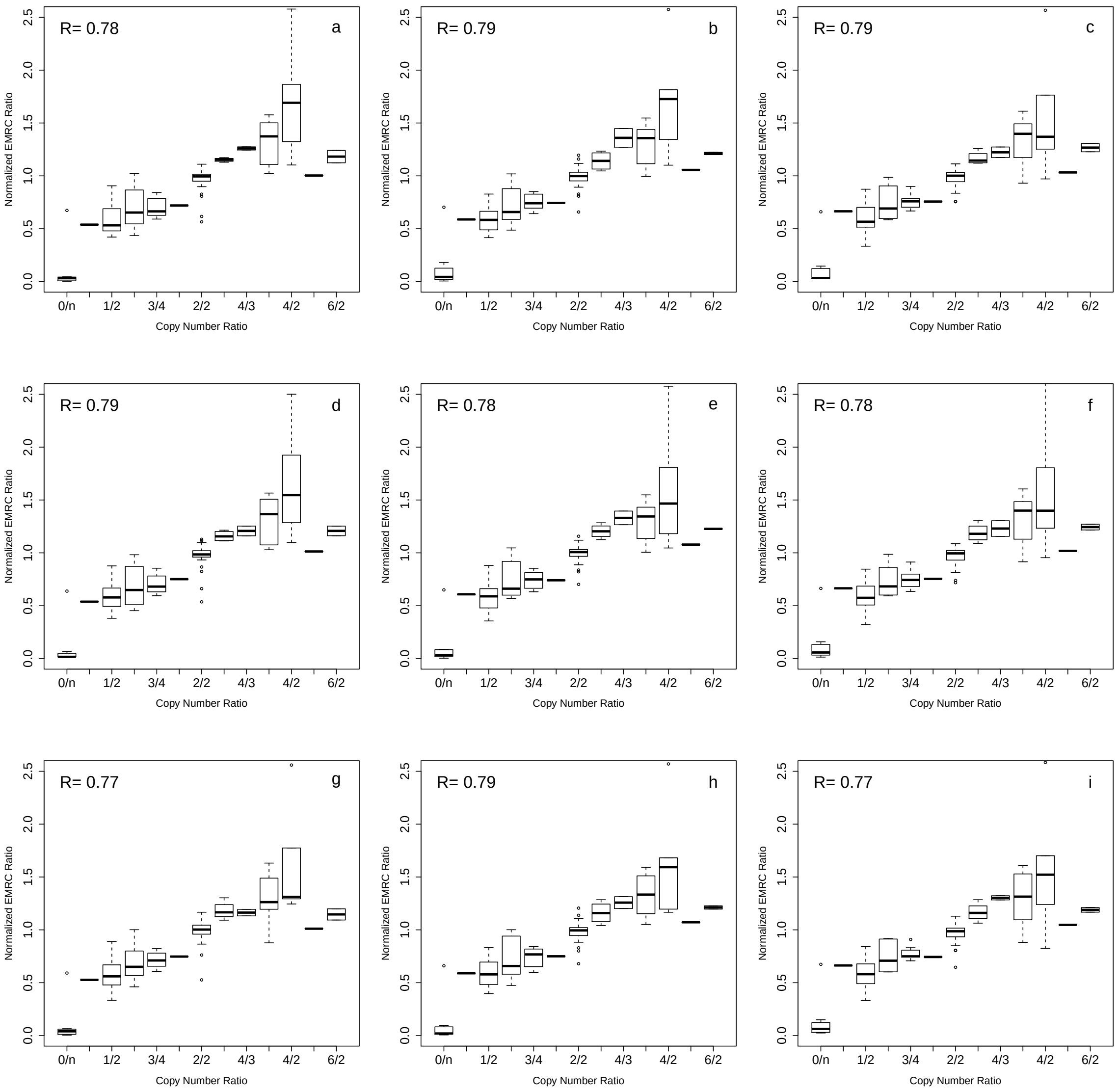

Supplemental Figure 65: The capability of EMRC ratio to predict the exact number of DNA copies of a CNV region as a function of alignment algorithm and read length. All the analyses were performed using several broad genomic regions that were previously reported to have copy numbers equal to 0, 1, 2, 3, 4,5 and 6 by McCarroll et al. (2008) in the four samples from the 1000 Genomes Project. R is the Pearsons correlation coefficient between the copy number predicted by McCarroll and the normalized EMRC ratio. The analysis were performed with BWA (a, b, c), Bowtie2 (d, e, f) and SOAP2 (g, h, i) aligners and with read length 100 (a, d, g), 75 (b, e, h) and 50 (c, f, i) bp.
